# A 75,000-y-old Scandinavian Arctic cave deposit reveals past faunal diversity and paleoenvironment

**DOI:** 10.1073/pnas.2415008122

**Published:** 2025-08-04

**Authors:** Samuel J. Walker, Aurélie Boilard, Mona Henriksen, Edana Lord, Marius Robu, Jan-Pieter Buylaert, Liselotte M. Takken Beijersbergen, Lene Synnøve Halvorsen, Adriana M. Cintrón-Santiago, Emma Katrin Onshuus, Christopher Alan Cockerill, Gabor Ujvari, László Palcsu, Marjan Temovski, Jenny Maccali, Henriette Linge, Jesper Olsen, Sverre Aksnes, Anastasia Bertheussen, Ola Lygre, Inger G. Alsos, Love Dalén, Bastiaan Star, Anne Karin Hufthammer, Thijs van Kolfschoten, Stein-Erik Lauritzen, Trond Klungseth Lødøen, Sanne Boessenkool

**Affiliations:** ^a^Centre for Ecological and Evolutionary Synthesis, Department of Biosciences, University of Oslo, Oslo N-0371, Norway; ^b^Department of Archaeology and Anthropology, Bournemouth University, Bournemouth BH12 5BB, United Kingdom; ^c^Faculty of Environmental Sciences and Natural Resource Management, Norwegian University of Life Sciences, Ås N-1432, Norway; ^d^Centre for Palaeogenetics, Stockholm SE-106 91, Sweden; ^e^Department of Zoology, Stockholm University, Stockholm SE-106 91, Sweden; ^f^Emil Racoviţă Institute of Speleology, Department of Karstology, Karst Inventory and Cave Protection, Bucharest 050711, Romania; ^g^Research Institute of the University of Bucharest, The Earth, Environmental and Life Sciences Division, Bucharest 050633, Romania; ^h^Department of Physics, Technical University of Denmark, Roskilde DK-4000, Denmark; ^i^Department of Natural History, University Museum of Bergen, University of Bergen, Bergen N-5020, Norway; ^j^Institute for Geological and Geochemical Research, HUN-REN Research Centre for Astronomy and Earth Sciences, Budapest H-1112, Hungary; ^k^Isotope Climatology and Environmental Research Centre, HUN-REN Institute for Nuclear Research, Debrecen H-4026, Hungary; ^l^Department of Earth Sciences and Bjerknes Centre for Climate Research, University of Bergen, Bergen N-5020, Norway; ^m^Centre for Early Sapiens Behaviour, University of Bergen, Bergen N-5020, Norway; ^n^Aarhus AMS Centre, Department of Physics and Astronomy, Aarhus University, Aarhus DK-8000, Denmark; ^o^Department of Cultural History, University Museum of Bergen, University of Bergen, Bergen N-5020, Norway; ^p^The Arctic University Museum of Norway, The Arctic University of Norway, Tromsø, Norway; ^q^Department of Bioinformatics and Genetics, Swedish Museum of Natural History, Stockholm SE-104 05 N-9006, Sweden; ^r^Faculty of Archaeology, Leiden University, Leiden 2333 CC, Netherlands; ^s^Joint International Research Laboratory of Environment and Social Archaeology, Shandong University, Qingdao 266237, China

**Keywords:** Weichselian, paleozoology, bulk-bone metabarcoding, ancient DNA, Europe

## Abstract

During the last glacial period (~118 to 11.7 ka), the Arctic has been characterized by a major redistribution of flora and fauna as a consequence of extreme climatic fluctuations, with associated glacial advances and retreats, sea-level changes, and shifting sea ice extent. In the high-latitude regions of Northern Europe that are currently subject to rapid climate warming, we lack a comprehensive understanding of faunal biodiversity in the last glacial period due to the extreme rarity of preserved organic remains. Here, we present a stratified sediment deposit with a diverse faunal composition preserved in a bone-bearing layer in Arne Qvamgrotta, part of the Storsteinhola cave system (68.10° N 16.38° E) in Northern Norway. Chronological analyses of sediments and bones including radiocarbon, optically stimulated luminescence, uranium–thorium, and phylogenetic dating place the faunal assemblage in Marine Isotope Stage 5a (MIS 5a, Odderade interstadial, ~85 to 71 ka). Combining comparative osteology and bulk-bone metabarcoding, we identify 46 taxa, including mammals, birds, and fish, with several species not previously found in Fennoscandia. The fauna implies a nonanalogous cold-adapted coastal community, with close proximity to sea ice and nearby freshwater bodies. Mitogenome analyses of a subset of taxa identify extinct lineages which attest to a lack of habitat tracking and the absence of a local refugium during the subsequent fully glaciated periods. This faunal record demonstrates long-term faunal dynamics and coastal environmental conditions during MIS 5a in the European Arctic.

## Significance

Paleoarchives provide an opportunity to understand long-term biological responses to climate fluctuations, yet sedimentary records going back more than 10,000 y are extremely rare in previously glaciated areas such as the Arctic. Combining osteology and ancient DNA, we present an ~75,000-y-old faunal assemblage preserved in a cave in Northern Norway, revealing a coastal Arctic ecosystem differing from the characteristic Glacial megafauna. The cold-adapted faunal community with marine and terrestrial mammals, birds, and fish represents the oldest preserved faunal assemblage in the European Arctic, filling a significant void in our understanding of biodiversity and the environment during a period of dramatic climate change.

The Arctic is highly susceptible to rapid climate change and currently the fastest warming region on the globe (1, 2), thereby placing fauna and flora under severe pressure (3). In northernmost Europe, the region has faced dramatic alterations in the last glacial period (4, 5), including extreme climatic fluctuations and changes in sea level during the Eemian interglacial [Marine Isotope Stage (MIS) 5e; 130 to 118 thousand years ago (ka)] and the following Weichselian glacial [MIS 5d–2; ~118 to 11.7 ka (6, 7)]. The Eemian interglacial is one of the periods with the highest annual temperatures during the last 800 ka [1 to 2 °C higher than present (8–10)]. In contrast, thick ice sheets covered much of the high latitude areas in the Early Weichselian stadials (Herning; MIS 5d and Rederstall; MIS 5b), which retreated during the intervening warmer interstadials [Brørup; MIS 5c; ~104 to 90 ka and Odderade; MIS 5a; ~85 to 71 ka (11, 12)]. Reconstructing the paleoecological history of this region before the major ice sheet advances of MIS 4 would provide a unique understanding of the expansion and contraction of taxa during times when regions repeatedly became inhabitable. However, our understanding of high-latitude paleoecological history of glaciated areas predating the Last Glacial Maximum [LGM; 26 to 19 ka (13)] is extremely poor due to the rarity of deposits with organic remains (11, 14). Sediments were removed from much of the terrestrial landscape by glacial erosion and flushing of glacial meltwater (15, 16), and the barren rock that was left behind in the erosion and scouring zones of the ice sheet has severely limited pre-LGM paleoenvironmental and biodiversity reconstruction in the glaciated regions of the Arctic.

Knowledge of the high-latitude faunal history of the last interglacial-glacial cycle in Europe primarily stems from spot-finds of large mammals and faunal assemblages concentrated around the edge of the glacial zones such as in Poland and Britain (e.g., refs. 17–22). During the Eemian, taxa such as *Hippopotamus amphibius* (hippopotamus) and *Panthera leo* (lion) were found as far north as Britain (23), while the Early Weichselian Eurasian mammoth steppe fauna was dominated by *Mammuthus primigenius* (woolly mammoth)*, Equus ferus* (wild horse), and *Coelodonta antiquitatis* (woolly rhinoceros) (e.g., refs. 17, 18, 24). Toward the west, a handful of British Early Weichselian assemblages lack the characteristic megafaunal component of mainland Europe and show the occurrence of temperate species [e.g., *Dama dama* (fallow deer) and *H. amphibius*] that only survived into MIS 5c, being replaced by colder adapted species such as *Vulpes lagopus* (Arctic fox) and *Rangifer tarandus* (reindeer) directly after MIS 5b (19–22, 25). Also, in central Fennoscandia—the region including the Scandinavian and Kola peninsulas, spot-finds of cold-adapted species, overwhelmingly *M. primigenius*, indicate a mammoth steppe fauna during the Middle-Late Weichselian (26–33), and a modern coastal Arctic community on the south-west coast of Norway during MIS 3 [Skjonghelleren and Hamnsundhelleren (30, 34, 35)]. Together, these finds support the prevalence of well-known cold-adapted mammoth steppe taxa at high latitudes during the Middle to Late Weichselian interstadials. However, in this region, we lack faunal assemblages dating to the Early Weichselian interstadials with important environmental indicators such as small mammals, birds, and fish.

**Fig. 1.**
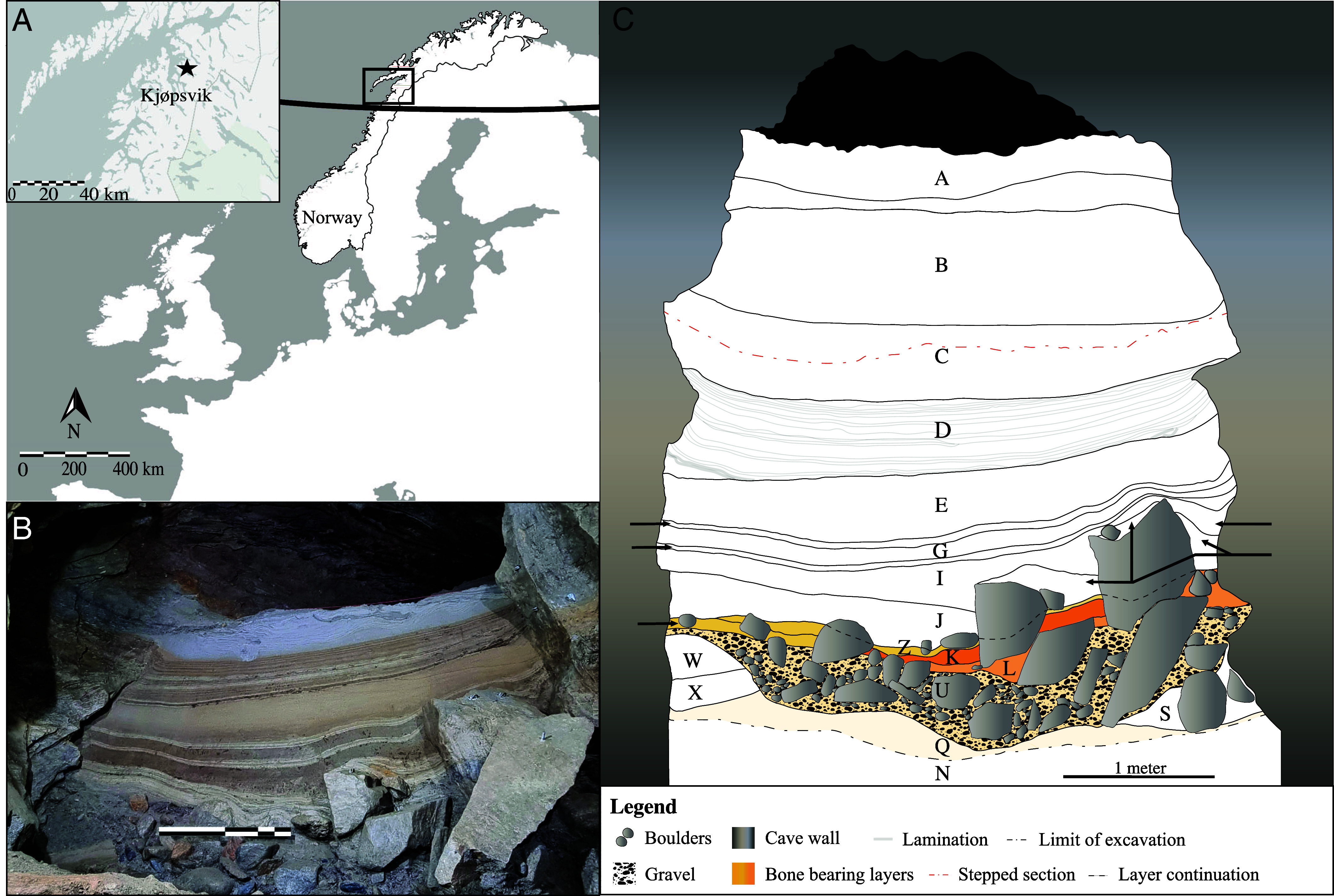
(*A*) The location of the Storsteinhola cave system in Kjøpsvik village in the municipality of Narvik, Nordland, Norway, shown on a map of Fennoscandia and surrounding countries. The black line represents the Arctic Circle. (*B*) Photo of the profile in Arne Qvamgrotta postexcavation, with a one-meter scale. Layers A, B, and half of Layer C are not visible as the upper section had to be set back to prevent collapse of the profile. (*C*) Drawing of the full profile in Arne Qvamgrotta. Bone-bearing layers are colored in yellow and orange, where the darker coloration indicates higher bone frequency. Thin gray lines in Layer D represent laminations. Large boulders are shown in gray and gravel in black. The red line in Layer C shows where the section was set back for safety purposes, as seen in (*B*).

Caves are often the sole time capsules of past fauna, yet almost all caves at high latitudes have been subject to flushing by glacial meltwater and rarely contain pre-LGM sediments. A unique exception is Arne Qvamgrotta (formerly Norcemgrotta), a karst conduit in Kjøpsvik, municipality of Narvik, Nordland, Northern Norway (Fig. 1*A*), located at 47 m a.s.l. well below the Late Glacial marine limit of ~90 m a.s.l., and covered by a thick ice sheet during the stadials (*SI Appendix*, Fig. S1). This conduit is part of the Storsteinhola karst system and connected to the surface through Nygrotta (57 m a.s.l.) by a sediment-filled passage [(36–39); *SI Appendix*, Text S1 and Fig. S2]. In 1991, the construction of a tunnel revealed a ~13 m thick sediment deposit in Arne Qvamgrotta, containing stratified layers of sands, gravels, boulders, and diamictic sediments with subfossil bone remains preserved in some of the layers. A set of geomorphological features and processes have helped preserve the sediment sequence in Arne Qvamgrotta despite being glaciated several times with subsequent flushing by glacial meltwater. Notably, the passage is located at a higher elevation relative to the entire Storsteinhola cave system (37) and can only be hydrologically active under full glaciation or deglaciation floods. In an ice-free setting, the cave system therefore has ample capacity to drain floodwaters elsewhere, leaving the deposited sediments at the higher levels untouched. Moreover, high fluvial activity currently occurs well below the level of Arne Qvamgrotta [(40); *SI Appendix*, Fig. S2], which due to the large dimensions (>5 m diameter) of the drainage routes has likely been the case since the beginning of the last glacial period.

Preliminary investigations of Arne Qvamgrotta discovered a bone assemblage dated to a minimum of 70 ka, and both MIS 5e (Eemian) and MIS 5a (Odderade) were postulated (37, 38). Here, we recover a detailed stratigraphic record and over 6,000 bone fragments from the sediment sequence. We applied a unique combination of dating methods with optically stimulated luminescence (OSL), uranium–thorium (U/Th), radiocarbon (AMS ^14^C), as well as phylogenetic dating to reconstruct the chronology of sedimentation and the bone assemblage. Using bulk-bone metabarcoding (BBM) together with the more traditional identification of vertebrate remains by comparative osteology, we recovered an exceptionally rich faunal community record representing 46 terrestrial, freshwater, and marine taxa including mammals, birds, and fish, dated to MIS 5a. These results provide paleoecological insights into the European Arctic environment and faunal diversity of the Early Weichselian interstadial MIS 5a (~85 to 71 ka) before the subsequent ice advances.

## Results

### The Stratigraphic Sequence.

**Fig. 2.**
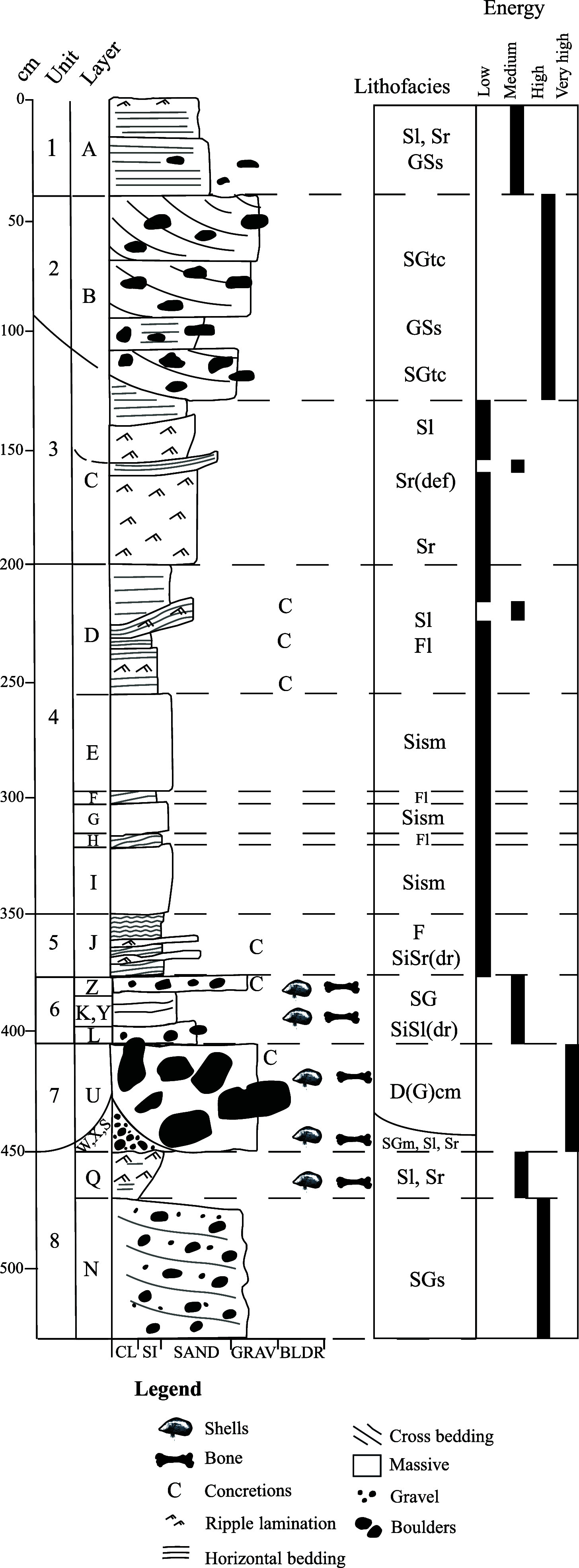
Sediment log of Arne Qvamgrotta with interpreted depositional flow energy. CL = clay, SI = silt, GRAV = gravel, BLDR = boulder. Lithofacies codes according to *SI Appendix*, Table S1.

The analyzed Arne Qvamgrotta sequence area of ~8.5 m^2^ reaches a depth of ~5 m (*SI Appendix*, Text S2 and Fig. S3), intercepting 20 stratigraphic layers that are grouped into eight larger descending units of sedimentation (Figs. 1 *B* and *C* and 2 and *SI Appendix*, Tables S1 and S2). The sediment sequence comprises mainly subhorizontal layers of glaciofluvial sandy gravel and glaciolacustrine very fine sand, silt, and clay (Figs. 1*C* and 2 and *SI Appendix*, Text S3 and Table S2), typical of a cave environment located in a previously glaciated region (41–43). The bone-bearing Unit 6 differs from the rest of the analyzed deposits, with four discontinuous layers filling the space between boulders and underlying depressions, and it was extensively sampled (*SI Appendix*, Fig. S4). In addition, we excavated at the outer end of the sediment-filled passage of the same cave system, named Nygrotta, resulting in a sequence area of ~3 m^2^ reaching a depth of 3 m, intercepting 10 stratigraphic layers grouped into five larger units (*SI Appendix*, Text S3, Fig. S5, and Table S3). Bones from Nygrotta were only recovered from the upper 50 cm, dated to the end of the Younger Dryas (~12.9 to 11.7 ka) into the Early Holocene [11.7 to 8.2 ka; see (39)]. The sediment sequence at Nygrotta is correlated to the upper Units 2 and 3 in Arne Qvamgrotta (*SI Appendix*, Fig. S6).

### Chronology.

**Fig. 3.**
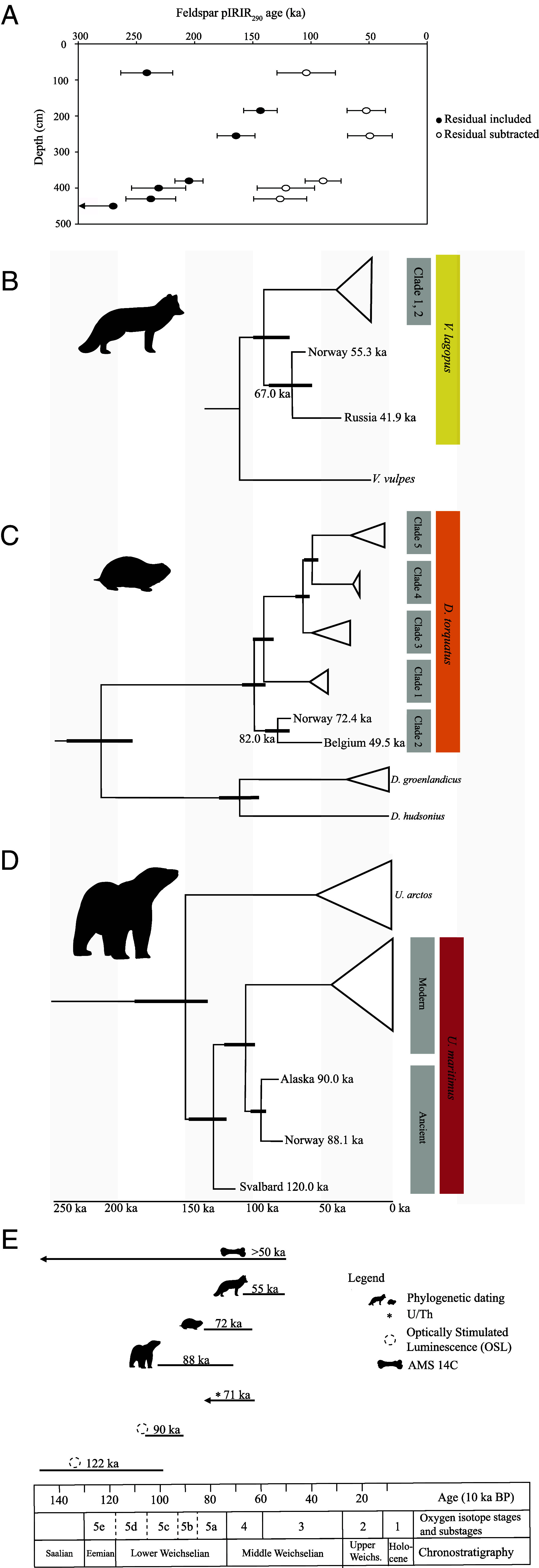
(*A*) Feldspar post-IR_50_ IRSL_290_ ages with and without residual dose (339 ± 36 Gy) subtracted. Residuals were estimated from the intercept on the *y*-axis on an IR_50_ to pIRIR_290_ D_e_ plot (*SI Appendix*, Text S4). Error bars represent age uncertainty comprising both random and systematic components and are given at 1 sigma. Uncertainty on the luminescence ages is large (≥15 ka at 1 sigma) and needs a significant residual dose correction. The age for the lowermost sample is a minimum age. (*B*) Dated mitochondrial phylogenies of *Vulpes lagopus* (Arctic fox), with *Vulpes vulpes* (red fox) as the outgroup, (*C*) *Dicrostonyx torquatus* (collared lemming) with *Phenacomys intermedius* (heather vole) as the outgroup (*P. intermedius* was removed for visualization purposes), and (*D*) *Ursus maritimus* (polar bear) and *U. arctos* (brown bear) with *U. americanus* (black bear) as the outgroup (*U. americanus* was removed for visualization purposes), obtained using BEAST v1.10.4. Full versions of all three phylogenies can be found in the *SI Appendix*, Figs. S10, S12, and S14). The specimens from Arne Qvamgrotta are labeled “Norway”. Date intervals for nodes are represented by thicker lines and actual dates represent median ages. Node support is >0.99 for all nodes and time is presented as ka. The Arne Qvamgrotta specimens were dated to ~55 ka (95% HPD 67.7 to 50.0 ka; *V. lagopus*), ~72 ka (95% HPD 81.5 to 63.5 ka; *D. torquatus*) and ~88 ka (95% HPD 102.2 to 72.5 ka; *U. maritimus*). (*E*) Overview of the obtained ages of the bone assemblage and bone-bearing layers in Arne Qvamgrotta, using phylogenetic dating, U/Th, OSL, and AMS ^14^C, with oxygen isotope stages and substages together with chronostratigraphy. Location on the profile for all samples can be found in the *SI Appendix*, Figs. S4 and S5.

Luminescence dating employed a post-IR IRSL protocol on K-rich feldspar extracts (44), corrected for a residual pIRIR_290_ dose at deposition based on the IR_50_ to pIRIR_290_ D_e_ relationship (*SI Appendix*, Text S4, Tables S4–S6, and Fig. S7). At Nygrotta, three residual-corrected feldspar pIRIR_50,290_ luminescence ages of 104 ± 25, 52 ± 16 and 49 ± 19 ka were obtained from Units N2, N3, and N4, respectively (Fig. 3*A* and *SI Appendix*, Fig. S5 and Table S6). The upper sample NY22-40 (104 ± 25 ka) in Unit N2 is clearly too old and is considered an outlier (*SI Appendix*, Text S4). In Arne Qvamgrotta, Unit 6 is dated by two radiocarbon dates of bones with nonfinite ages (>50 ka; *SI Appendix*, Text S4, and Table S7), two residual-corrected luminescence ages of 122 ± 25 and 90 ± 15 ka (Fig. 3*A* and *SI Appendix*, Table S6) and a U/Th minimum age of 71 ± 9 ka (*SI Appendix*, Text S4, Tables S8 and S9, and Figs. S8 and S9). Uncertainty on all luminescence ages is large (≥15 ka at 1 sigma) and needs a significant residual dose correction (*SI Appendix*, Text S4). Phylogenetic dating of sequenced mitochondrial genomes was performed for *V. lagopus*, *Dicrostonyx torquatus* (collared lemming), and *Ursus maritimus* (polar bear) specimens recovered from Unit 6 (see The fauna and flora below; *SI Appendix*, Text S4 and Table S10). *Vulpes lagopus* was molecularly dated to ~55 ka (95% HPD: 67.7 to 50.0 ka) and placed in an extinct basal lineage together with a ~30 ka *V. lagopus* specimen from Russia (Fig. 3*B* and *SI Appendix*, Table S11 and Fig. S10). Similar analyses for *D. torquatus* dated the specimen to ~72 ka (95% HPD: 82.5 to 63.5 ka) and placed it in the basal Clade 2 of the phylogeny along with a ~50 ka specimen from Belgium (Fig. 3*C* and *SI Appendix*, Table S11 and Figs. S11 and S12). *Ursus maritimus* grouped with the two other ancient polar bears sequenced to date outside of the modern clades, and its age was estimated at ~88 ka (95% HPD: 102.2 to 72.5 ka; Fig. 3*D* and *SI Appendix*, Figs. S13 and S14 and Tables S11 and S12). Finally, Units 7 and 8 are dated by luminescence to 127 ± 23 ka (Fig. 3 and *SI Appendix*, Table S6) and >130 ka, respectively (Fig. 3 and *SI Appendix*, Text S4 and Table S6). Further dating was attempted using cosmogenic nuclide burial dating, but this was unsuccessful (*SI Appendix*, Text S4 and Table S13).

### Taphonomic Indicators.

A total of 6,191 bone fragments were recovered from Units 6 to 8 (Layers Y, Z, K, L, U, and Q), with the majority (99.2%) from Unit 6 [Layers K (40.8%), K/L (3.5%), L (27%), L/U (7.8%), Y (12.4%), and Z (7.7%); Figs. 1*C* and 2]. The bone assemblage is highly fragmentary, and by using comparative osteology only 350 fragments (5.7%) could be identified to family taxonomic level or higher, with the exception of the order Rodentia (*SI Appendix*, Text S5 and Table S14). Bone element representation indicates a bias toward isolated molars (specifically for small mammals; *SI Appendix*, Table S15). The identified fragments show no clear indications of weathering, erosion, abrasion, or gnawing, with the exception of puncture marks consistent with predation observed on one bird coracoid. The unidentified fragments show signs of slight to moderate abrasion and rounding [based on (45)]. Taphonomy indicating hominin presence such as burning or tool marks was not observed on any of the remains, implying that these bones originate from a naturally formed deposit without anthropogenic influence.

### The Fauna and Flora.

**Fig. 4.**
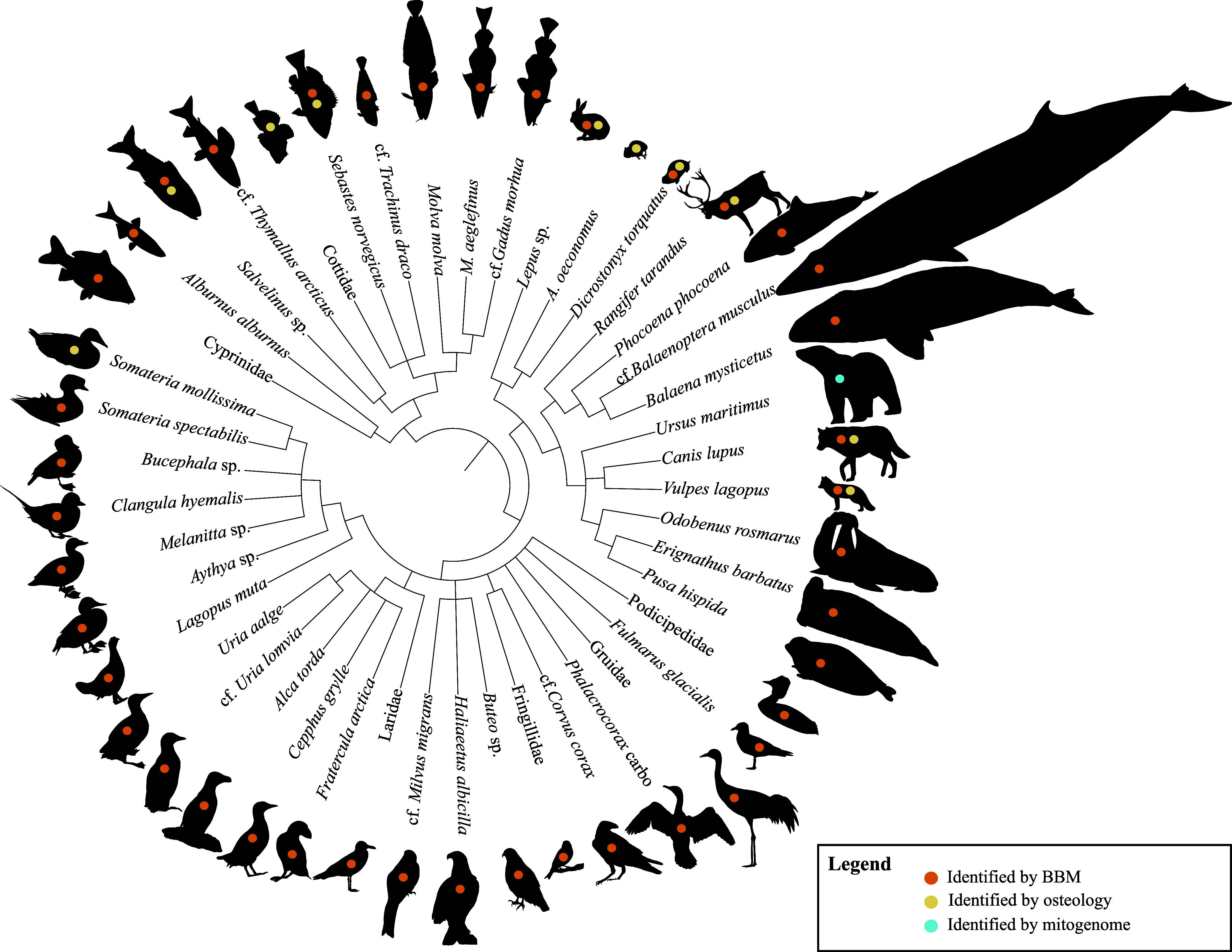
Dendrogram presenting the fauna identified from the bone assemblage recovered from Arne Qvamgrotta. Only the lowest taxonomic rank is presented for visual purposes (see *SI Appendix*, Table S21 for the complete list of identified taxa). *M. aeglefinus* (*Melanogrammus aeglefinus*) and *A. oeconomus* (*Alexandromys oeconomus*) are abbreviated to fit within the limit of the figure. Taxa were identified through a combination of comparative osteology, bulk-bone metabarcoding, and mitogenome phylogeny (*SI Appendix*, Table S21), indicated on the animal silhouettes by yellow, orange, and blue dots, respectively. The dendrogram phylogeny was generated using scripts from ref. 53, the NCBI database, and create_tree_from_curated_list.py (https://github.com/frederikseersholm/blast_getLCA/blob/master/README.md).

The bone assemblage was analyzed by comparative osteology and BBM, and consists of 46 taxa from 30 families, 37 genera, and 33 species, including Aves (birds; 23 taxa), Mammalia (mammals; 13 taxa), and Pisces (fish; 10 taxa; Fig. 4 and *SI Appendix*, Texts S5 and S6, and Tables S14–S21). Seven taxa were identified by both comparative osteology and BBM, three taxa exclusively by osteology and 36 taxa only by BBM (*SI Appendix*, Text S7 and Table S21). In addition, we identified one bivalve, one gastropod, one crustacean, and one plant taxon. The faunal species composition did not differ between the discontinuous layers within Unit 6, and the assemblage is therefore regarded as a single faunal unit. The birds recorded represent the most diverse Early Weichselian avifaunal assemblage in the Arctic to date, comprising 23 taxa from 12 families, 16 genera, and 14 species (Fig. 4 and *SI Appendix,* Table S21). Seabirds are the dominant group represented, in particular Anatidae (ducks) and Alcidae (auks). The Anatidae identified are almost exclusively sea ducks [such as *Somateria spectabilis* (king eider) and *Clangula hyemalis* (long-tailed duck)]. Diving ducks are represented by *Aythya* which is likely one of the two more northern species *Aythya marila* (greater scaup) or *Aythya fuligula* (tufted duck); (46). Within the Alcidae, we identify all extant Northern European species, except for *Alle alle* (little auk) which breeds in the high Arctic and has been recorded 100 km further north on Andøya during the LGM [~20 ka; (47)]. Land-dwelling birds include *Lagopus muta* (rock ptarmigan), *Corvus corax* (common raven), Gruidae (cranes), and Fringillidae (finches). The identification of Gruidae may represent *Grus grus* (common crane), the only species in this family that still breeds in Northern Norway (48). Alternatively, it may represent the critically endangered *Leucogeranus leucogeranus* (Siberian crane), which breeds in tundra wetlands and likely had a wider distribution during parts of the last glacial period (49, 50). The *Buteo* genus identified is probably the most northern species in Norway, *Buteo lagopus* [rough-legged buzzard; (46, 51)]. The identification of *Milvus* stands out as this genus only occurs as a vagrant in Norway today (52), with the current distribution of *Milvus migrans* (black kite) being more easterly restricted toward the White Sea and the edge of the Kola Peninsula (51).

Mammalia are also well represented within the faunal diversity, with 13 taxa identified from 10 families, 13 genera, and 12 species (Fig. 4 and *SI Appendix,* Table S21). Small mammals include *Alexandromys oeconomus* (tundra vole) and *D. torquatus*, the latter currently extinct in Fennoscandia (54) and not previously found in Scandinavia. Larger terrestrial mammals include *V. lagopus, Lepus* sp. (hares), and *Rangifer tarandus*. A total of seven marine mammal species includes *Balaena mysticetus* (bowhead whale), *Balaenoptera musculus* (blue whale), *U. maritimus* (polar bear; *SI Appendix*, Figs. S13 and S14), and *Phocoena phocoena* (harbor porpoise). Several of these [i.e., *U. maritimus, Pusa hispida* (ringed seal), *Erignathus barbatus* (bearded seal), *Odobenus rosmarus* (walrus), and *B. mysticetus*] have Arctic and subarctic distributions and are sea-ice associates (55, 56). *Phocoena phocoena* is also found in cold temperate and subpolar waters, but unlike the other sea mammals identified, it avoids areas with sea ice (57). Of the identified marine mammals, only *P. phocoena* and *B. musculus* are still found in the vicinity of Arne Qvamgrotta today.

Both freshwater and marine fish (Pisces) are represented with 10 taxa identified from eight families, eight genera, and seven species (Fig. 4 and *SI Appendix,* Table S21). Typical marine, cold-water adapted species include *Gadus morhua* (Atlantic cod), *Melanogrammus aeglefinus* (haddock), and *Sebastes norvegicus* (golden redfish). In addition, the genus *Salvelinus* most likely represents the cold-water species *Salvelinus alpinus* (Arctic char). Certain members of the Cottidae (sculpin) family are also adapted to cold-water conditions and have been found in other pre-LGM sites in Norway (30, 34, 35, 58), while *Molva molva* (common ling) and *Trachinus draco* (greater weaver) are typically regarded as more boreal species (59). Freshwater fish species are represented by *Alburnus alburnus* (common bleak), the family Cyprinidae (minnows), and *Thymallus arcticus* (Arctic grayling), whereby the latter can notably survive for long periods under ice (60). The phenotypic plasticity of *A. alburnus* is well documented, having recently been introduced widely in Europe (61), and it is known from assemblages dated to MIS 5a in the United Kingdom (20), and the Eemian in Poland (62, 63). All freshwater fish taxa with the exception of *T. arcticus* can still be found in the region today, and several have been identified in the Early Holocene assemblage from Nygrotta (39).

Marine invertebrates were identified from a total of 1479 shell fragments, representing Cirripedia (barnacles), specifically large Balanoide [e.g., *Balanus balanus* (rough barnacle)], *Mytilus* sp. (mussels), and *Littorina* sp. (sea snails; *SI Appendix*, Text S8 and Table S22). Pollen (eight samples) and sedimentary ancient DNA (*sed*aDNA, 12 samples) analyses for reconstruction of the flora yielded two wood fibers in one pollen sample from Layer L, identified to the Pinaceae family (the pines; sample AQP6; *SI Appendix*, Text S9, Table S23, and Fig. S15*A*). This sample also contained 4,845 charcoal dust particles (*SI Appendix*, Table S23 and Fig. S15*B*), accounting for 74% of all charcoal dust identified from the eight samples. None of the charcoal particles displayed any identifiable traits. Pollen-sized charcoal particles are not uncommon (e.g., ref. 64) and may derive from forest fires and blown into caves together with pollen, though anthropogenic origin cannot be excluded. None of the samples contained detectable plant DNA (*SI Appendix*, Text S10 and Table S24).

## Discussion

Arne Qvamgrotta harbors the first known sediment sequence in the European Arctic with a diverse faunal assemblage predating the LGM. Considering all the chronological information, we conclude that the most likely window for deposition of the bone-bearing layers was during MIS 5a (~85 to 71 ka). The 46 taxa we identify from marine, freshwater, and terrestrial ecosystems include the earliest evidence of birds and fish inhabiting Fennoscandia during the last glacial period, as well as species never observed in this region before and currently extinct genetic lineages. Below we discuss the implications of these findings from geological, biological, and climatic perspectives.

### Depositional History of the Stratigraphic Sequences in Arne Qvamgrotta.

Chronology of the analyzed sediment sequence dates back to at least ~122 ka inferred from OSL, U/Th, AMS ^14^C, and phylogenetic analyses (Fig. 3). This sequence has been deposited both during cycles with high-energy depositional environments (e.g., streaming water and sliding beds, which are fluid diamictons injected through a cave conduit) and during low-energy depositional environments (e.g., subaerial with weathering of rock and pools of stagnant water). Specifically, the gravelly and sandy layers within Unit 1 (Layer A), Unit 2 (Layer B), and Unit 8 (Layers N, Q, and W) are most likely glaciofluvial with deposition during high hydrological activity (43, 65), when the cave system was subject to subglacial drainage (41, 66). The sliding-bed deposit with imbricated boulders within Unit 7 must have required even higher water energy causing movement of mass into the cave system [similar to (41, 67)]. In these periods, the glacial margin must have been close with high water discharge and a hydraulic gradient (42). In contrast, the laminated fine-grained sediments in Units 4 (Layers D, F, and H) and 5 (Layer J) have been deposited by suspension settling with the intervening coarser sandy layers (C, E, G, and I) during episodes with medium through-flow of subglacial water masses. These layers have likely been deposited during periods of low hydrological activity, for instance when the glacier above the cave was thick and the glacial margin further away, filling the cave with standing or slow-moving water (34, 35, 42, 68–70). Such deposition is similar to other caves located in formerly glaciated areas (35, 70, 71). During any nonglaciated periods, other depositional environments prevail. For instance, the vertical position of the angular boulders draped by Unit 6 indicates that they fell from the roof of the cave, likely due to frost weathering (43). Unit 6 (Layers Z, Y, K, and L), consisting of discontinuous layers of sorted sediments with a high bone content, is probably deposited under subaerial conditions with occasional water flows that brought silt and sand into small depressions. Similar depositional settings have been recorded at the sites of Skjonghelleren and Hamnsundhelleren (34, 35).

The chronology and sedimentological analyses of the sequence support discontinuous deposition from the Late Eemian up to the Holocene. The complexity of depositional processes in caves can lead to mixed deposits, whereby material transported from various locations and time periods is redeposited within the cave system. In Arne Qvamgrotta our analyses that focus specifically on the bone-bearing layers (Unit 6), however, support that mixing, at least on any large scale, is not the primary process in the deposition of the assemblage. Minimum ages of the assemblage are constrained by nonfinite AMS ^14^C dating of bones in Unit 6 (>50 ka) and U/Th dating of a concretion from Unit 5 to 71 ± 9 ka, while an upper boundary is provided by OSL dating of sediments (122 ± 25 ka and 90 ± 15 ka; Fig. 3). We complement these classical dating methodologies with independent phylogenetic dating of *V. lagopus*, *D. torquatus,* and *U. maritimus* specimens from Unit 6, which places these at ~55 ka (95% HPD 67.7 to 50.0 ka), ~72 ka (95% HPD 82.5 to 63.5 ka) and ~88 ka (95% HPD 102.2 to 72.5 ka), respectively (Fig. 3 and *SI Appendix*, Text S4, Table S11, Figs. S10, S12, and S14). The relatively young age of *V. lagopus* may be considered a minimum age due to the limited available comparative data, with only a single other sequence from an ancient specimen (Fig. 3*B* and *SI Appendix*, Text S4 and Fig. S10), and additional samples may help to refine this date in the future. The obtained dates, together with the identification of a largely Arctic fauna (see below) and a well-defined stratigraphy, are most parsimonious with an Early Weichselian MIS 5a origin of the faunal assemblage (*SI Appendix*, Fig. S1 for placement in a Norwegian context).

The Arne Qvamgrotta assemblage was likely formed through a combination of local water transport and predator–prey interactions. The lack of heavy abrasion and articulated remains [as for example seen at Westbury Cave, United Kingdom, where rounded bones were transported ~10 km (72)] shows that any transport into the cave would have been over a short distance, possibly caused by seasonal meltwater. Moreover, the few well-preserved bones in the deposit likely represent animals that have either died in-situ or were brought in by predators. Although there are no consistent signs of digestion, puncture marks, or gnawing, many taxa fit the present-day diet of the main predators identified [*U. maritimus*, *Canis lupus* (wolf), *V. lagopus, Haliaeetus albicilla* (white-tailed eagle)*, B. lagopus*], and both *V. lagopus* and *U. maritimus* are known to stockpile food (73, 74). Cetaceans and pinnipeds were exclusively identified through BBM, and these most likely represent scavenged individuals, resulting in high fragmentation of the bone remains. Alternatively, the high lipid content of marine mammal bones [especially cetaceans (75)] could lead to poor preservation and their typical underrepresentation in faunal assemblages (76). Although predator–prey interactions appear most likely to explain the accumulation of this assemblage, we cannot exclude that this record may have formed over thousands of years during which sea levels may have changed rapidly covering both a marine and a terrestrial phase. Nevertheless, given the consistent ages, the taphonomy of the bones and taxa associated with northern latitudes, we are confident that the entire assemblage represents a stratigraphically unbiased sample of the Early Weichselian interstadial fauna.

### A Cold Arctic Coastal Ecosystem During MIS 5a (~85 to 71 ka).

The recovered faunal assemblage representing both marine and terrestrial fauna at Arne Qvamgrotta is largely indicative of a coastal, Early Weichselian interstadial environment that comprises an open Arctic or subarctic tundra, with nearby perennial or seasonal sea-ice, freshwater lakes or rivers, and the presence of some pine trees in the region. We find no evidence of hominin presence, yet recent niche modeling suggests that the environment as far north as central Scandinavia could have been suitable for Neanderthals during the Eemian and interstadials MIS5c and 5a, and data from this northern region is to date still extremely scarce (77, 78). The presence of several ice-dependent sea mammals (*U. maritimus*, *P. hispida*, *E. barbatus*, *O. rosmarus*, and *B. mysticetus*) suggests close proximity to sea-ice polynyas (79, 80), while seasonality of sea-ice can be inferred from the presence of *P. phocoena* which is known to avoid areas of sea-ice (57). The latter is further supported by diatom and foraminifera proxies from MIS 5a in an ocean core from the Faroe Islands (located over a 1,000 km south from Arne Qvamgrotta) implying seasonal sea-ice and meltwater at the ocean’s surface (81). Fish and avifauna also indicate cold oceanic conditions, with fish taxa (e.g., *G. morhua, S. norvegicus*) that have current distributions up to the Barents Sea (82, 83), and a dominance of North Atlantic and Arctic seabirds, specifically Alcidae and Anatidae (84, 85). The associated terrestrial fauna complements the marine environment we infer, with small mammals (e.g., *D. torquatus*, *A. oeconomus*), large mammals (e.g., *V. lagopus*, *R. tarandus*), and birds (e.g., *L. muta*) that inhabit tundra regions. The mix of resident and migratory bird species suggests favorable year-round conditions. Moreover, glaciers must have melted sufficiently for continuous ice-free regions along the coast to support *R. tarandus*, and the freshwater fish attest the colonization of nearby freshwater bodies. Several of these freshwater fish are known to tolerate wide ranges of temperatures and habitats (e.g., *T. arcticus*, *A. alburnus*). Overall, combining osteology with aDNA BBM greatly enhances the information content that can be gained from these kinds of assemblages that are dominated by morphologically unidentifiable bone fragments, and especially for taxonomic groups that are more difficult to identify by osteology alone. Nevertheless, identifications based on BBM are limited by the available reference sequences and despite promising efforts to sequence all diversity of the tree of life these reference databases are still incomplete, especially for fish. The power of BBM and other genetic methods (e.g., shotgun sequencing and capture enrichment) can thus be expected to only increase in the future.

The coastal, cold-associated faunal community we reconstruct is different to the well-known mammoth steppe communities from the last glacial period, lacking signature terrestrial taxa such as *M. primigenius* and *Ovibos moschatus* (musk ox) that have been recorded from Fennoscandia during the Middle Weichselian (33). The absence of evidence does not necessarily translate into evidence of absence and we cannot exclude that *M. primigenius* and other taxa have been present in the area. The inferred climate, nevertheless, supports previous reconstructions based on interpretations of a tundra and Arctic steppe dominating western and Northern Europe during MIS 5a (11, 14, 86, 87). However, climate reconstructions at high latitude inland Fennoscandia based on plant and insect observations at Sokli, Northern Finland, have recently contrasted this view, suggesting a climate with July temperatures that are similar to, or warmer than, present day (88). It has been postulated that the terrestrial vegetation developments and oceanic productivity of MIS 5a were similar to those recorded for the warm stages of the Holocene in Northern Europe (81, 88, 89). The Sokli record most probably represented the climatic optimum of MIS 5a, whereas the beginning and end of MIS 5a must have been cooler and it is therefore most likely that the Arne Qvamgrotta faunal community represents one of these colder phases.

### The Identification of Now-Extinct Lineages in Arne Qvamgrotta.

Many of the mammalian taxa preserved in Arne Qvamgrotta are still found in the Arctic yet no longer in the vicinity of the cave, and all sequenced mitochondrial lineages are now extinct. Specifically, the discovery of *D. torquatus* fills a long-standing gap in the fossil record with the closest known fossil from Denmark dated to ~25 ka (90). *Dicrostonyx torquatus* diversified into five mitogenome lineages following the Eemian and underwent range expansions and local extinctions in response to climatic shifts (91–94). After the taxon’s disappearance from central Europe at the end of the last glacial period, the extant populations occur close to the White Sea and the Siberian tundra (54, 95–97). The clustering of the Arne Qvamgrotta *D. torquatus* with clade 2 places it with the oldest specimens found to date in Marie-Jeanne Cave in Belgium (92, 94); Fig. 3*C*]. Also, *V. lagopus* from Arne Qvamgrotta represents a phylogenetically basal lineage known from a single specimen from Russia dated to 41.9 ka [(98); Fig. 3*B*]. Finally, the recovery of *U. maritimus* represents the third oldest find of this species globally, after one specimen from Svalbard (130 to 100 ka; (99, 100) and one from Alaska [110 to 70 ka; (101)], and these ancient *U. maritimus* specimens fall outside the modern clades (Fig. 3*D*). The identification of extinct lineages of *D. torquatus*, *V. lagopus,* and *U. maritimus* all attest to a lack of habitat tracking, as has previously been described for *V. lagopus* (98, 102, 103). Moreover, these data suggest that the west coast of Northern Norway was not a refugium for these taxa in periods of extreme cold during the subsequent fully glaciated periods.

Overall, the diverse faunal record of Arne Qvamgrotta, preserved in a well-stratified deposit, represents the first Early Weichselian MIS 5a assemblage from the glaciated regions of the Arctic. The combination of comparative osteology and BBM exposed a coastal, cold-adapted, Arctic faunal community with marine and terrestrial taxa not previously known from Fennoscandia (e.g., *D. torquatus*), and includes several taxa that suggest a nonanalogous community (e.g., *M. migrans* and *T. draco*). The assemblage challenges our traditional view of the fauna from the last glacial period, which is dominated by the signature megafauna of the mammoth steppe with relatively little knowledge of coastal communities or other taxonomic groups such as birds and fish. Moreover, the observation that all sequenced mitogenomes represent now-extinct lineages indicates significant faunal turnover in the Fennoscandian Arctic, due to a lack of habitat tracking and the absence of a local refugium during fully glaciated periods. Importantly, the observed faunal turnover would be underestimated if taxonomic diversity had been analyzed based on species identification alone, appealing for mitogenome analyses beyond mammals to investigate how widespread these turnovers are in the broader faunal community. In the absence of individual fossils such mitogenomes may in the future be reconstructed from direct shotgun sequencing or capture enrichment of bulk-bone samples. Finally, this rare Early Weichselian paleorecord contributes to our understanding of the underlying mechanisms of long-term biological response to climate change (104, 105), and reveals the resilience potential of coastal Arctic ecosystems. The cold-adapted community at Arne Qvamgrotta testifies that many species were able to recolonize the Arctic following fully glaciated periods, yet the extinction of lineages highlights the inability of fauna to adapt or migrate with major climatic events.

## Materials and Methods

### The Excavation and Sedimentology.

The excavation at Arne Qvamgrotta and the connected Nygrotta was conducted over two field seasons following standard archaeological excavation practice (106, 107). An area excavation approach was adopted to expose a vertical profile that cut perpendicular to the cave walls. A grid system was implemented and orientated across the sediments dividing the site into 1 m^2^ units with X running north–south and Y running east–west. We followed a stratigraphic-mechanical procedure and sediments were wet sieved through 4 and 2 mm mesh. In Arne Qvamgrotta Units 1 to 5 were sterile glacial sediments and only a subsample was sieved, while bone-bearing sediments (Units 6 to 8) were completely sieved. At Nygrotta, an area of 3 m^2^ was opened and a column of 50 × 50 cm was fully sieved (detailed excavation methodology can be found in the *SI Appendix*, Text S2). Sediments were dried and later stored at 4 °C before extracting bone and shell fragments off-site using magnification lamps. The vertical sections at Arne Qvamgrotta and Nygrotta were lithostratigraphically logged and drawn, documenting sediment properties as grain size, structure, and texture. Layers K/L and L/U are at the interface between their respective layers, and as few bones are recovered from Layer U we place L/U in Unit 6 with the bulk of the faunal remains. Detailed site descriptions and sediment analyses can be found in the *SI Appendix*, Text S3.

### Dating.

To establish the chronology of the analyzed Arne Qvamgrotta deposit, five dating methods were used: AMS ^14^C, optically stimulated luminescence (OSL), U/Th, cosmogenic nuclide burial dating, and phylogenetic dating (see *SI Appendix*, Texts S4 and S6 for detailed methods). Seven bone specimens were AMS ^14^C dated at the Oxford Radiocarbon Accelerator Unit (ORAU) in Oxford (*SI Appendix*, Table S7). Seven OSL samples were selected from distinct sand-rich layers, with four from Arne Qvamgrotta (Layers N, K, Z, and U; *SI Appendix*, Table S4) and three from Nygrotta (Layers ND, NG, and NI; *SI Appendix*, Table S4). The OSL samples were analyzed at the Nordic Laboratory for Luminescence Dating (DTU Physics, Roskilde). Five calcareous concretions were selected for U/Th dating (*SI Appendix*, Table S8). Because of the high detrital Th content, two sample preparation methods were used [(108, 109); *SI Appendix*, Text S4], and the ages were calculated using the “isochron” approach [(110); *SI Appendix*, Text S4, Figs. S8 and S9 and Table S9]. Four samples were selected for ^26^Al/^10^Be burial dating [see (111); *SI Appendix*, Text S4 and Table S13]. Sample preparation was carried out at the Cosmogenic Nuclide Preparation Facility, University of Bergen, and Al and Be targets were analyzed at Aarhus AMS Centre, but the very low ^10^Be concentrations were not sufficiently precise to provide meaningful nuclide ratios for geochronological information. Phylogenetic dating of *V. lagopus*, *D. torquatus,* and *U. maritimus* was done using Bayesian reconstruction of phylogenetic trees with BEAST v.1.10.4 [(112); *SI Appendix*, Text S4; see phylogenetic analyses below].

### Comparative Osteology, Pollen, sedaDNA, and Shell Methodology.

The osteological identifications were achieved through comparative osteology, using the modern skeletal reference collection held at the Department of Natural History, University Museum of Bergen. Species abundance was quantified based on the Number of Identified Specimens (NISP), Minimum Number of Individuals (MNI), and overall number of taxa (*SI Appendix*, Text S5 and Table S14). Taphonomic markers were recorded when present following Fernández-Jalvo and Andrews (45). Shells were identified, counted, and weighed (*SI Appendix*, Text S8). For the pollen analyses, we selected eight samples from Units 6 to 8 which were prepared following standard procedures with KOH and HF to remove minerogenic particles (*SI Appendix*, Text S9). DNA was extracted from 12 sediment samples and plant DNA was amplified following the methods in refs. 47, 113, and sequences were matched against the four DNA reference libraries PhyloNorway (113), PhyloAlps (114), ArcborBryo (115–117), and EMBL release 143 (*SI Appendix*, Text S10).

### Bulk-Bone Metabarcoding.

A total of 47 bulk samples of unidentifiable bone fragments were divided into four groups (Mammalia, Pisces, Aves, and unidentifiable vertebrates). Bone fragments were milled, and DNA was extracted from up to three subsamples of ~110 mg each making a total of 78 DNA extracts (*SI Appendix*, Table S16). A selection of 16 individual bones were also analyzed by barcoding without being included in bulk samples (*SI Appendix*, Table S17). DNA extracts were amplified with primers targeting a short fragment of the 16S rRNA (mammals and fish) and 12S rRNA (birds). Extracts from samples with unidentifiable vertebrates were amplified with all three primer sets (*SI Appendix*, Text S6). All pre-PCR laboratory work was carried out in the dedicated ancient DNA laboratory at the University of Oslo. PCR products were pooled and libraries were built using the TruSeq DNA Nano library preparation kit before sequencing on the Illumina NovaSeq 6000 S4 (150 bp PE) at the Norwegian Sequencing Centre. Data were processed using the OBITools package v.1.2.12 [https://pythonhosted.org/OBITools/index.html, ref. 118; *SI Appendix*, Text S6]. For taxonomic assignment using ecotag reference libraries were built for each of the different primer pairs by performing an in silico PCR with ecoPCR (119) on the European Molecular Biology Laboratory (release downloaded February 2022, www.ebi.ac.uk/ena/browser/home) and the National Centre for Biotechnology Information (NCBI) Taxonomic database (www.ncbi.nlm.nih.gov/taxonomy). Sequences were further filtered in R v.4.3.0 (https://www.r-project.org/) and the resulting taxa list was reviewed by taxonomists (*SI Appendix*, Text S6 and Tables S19 and S20). Overrepresentation was avoided when counting the total number of taxa identified with comparative osteology and BBM by disregarding identifications above family level, and only counting family level identifications when there were no genus or species level identification within that specific family.

### Shotgun Sequencing and Phylogenetic Analyses.

DNA was extracted from one *V. lagopus*, 13 *D. torquatus*, and one *Ursus* sp. specimens for shotgun sequencing (*SI Appendix*, Text S4 and Table S10). Single-indexed sequencing libraries using the single-stranded Santa Cruz Reaction (SCR) library protocol (120) were built from the *Ursus* sp. and *D. torquatus* DNA extracts while a double-stranded library was built from the *V. lagopus* DNA extract following ref. 121. For *D. torquatus* a consensus mitogenome was generated following iterative mapping with MIA [mitogenome iterative assembler (122)] while sequences from *Ursus* sp. and *V. lagopus* were mapped to their respective reference genomes (*SI Appendix*, Text S4). Dated Bayesian phylogenies (123) of mitochondrial genomes were obtained with BEAST v.1.10.4 [(112); *SI Appendix*, Text S4]. Previously published modern and ancient genomes were included in all analyses and two independent runs were performed. The median tip date and 95% highest posterior density interval were calculated for each of the Arne Qvamgrotta specimens (*SI Appendix*, Text S4).

## Supplementary Material

Appendix 01 (PDF)

## Data Availability

All bone material will be deposited in the collection of the University Museum of Bergen. Raw read data are available in the European Nucleotide Archive (ENA, www.ebi.ac.uk/ena) (124). Metadata, results of the BLAST analysis, sequence files from ecotag output, and R scripts are available as an archived Github on Zenodo (125).
