# Supplementary material for: A 75,000-y-old Scandinavian Arctic cave deposit reveals past faunal diversity and paleoenvironment
Source: Proc Natl Acad Sci U S A. 2025 Aug 4;122(32):e2415008122. doi: 10.1073/pnas.2415008122 (PMC12358836; doi:10.1073/pnas.2415008122)
Supplement: Supplementary file 1 — Appendix 01 (PDF) [file pnas.2415008122.sapp.pdf]

PNAS

Supporting Information for

A 75 000 year old Scandinavian Arctic cave deposit reveals past faunal diversity and palaeoenvironment

Samuel J. Walker, Aurélie Boilard, Mona Henriksen, Edana Lord, Marius Robu, Jan-Pieter Buylaert, Liselotte M. Takken Beijersbergen, Lene Synnøve Halvorsen, Adriana M Cintrón-Santiago, Emma Katrin Onshuus, Christopher Alan Cockerill, Gabor Ujvari, László Palcsu, Marjan Temovski, Jenny Maccali, Henriette Linge, Jesper Olsen, Sverre Aksnes, Anastasia Bertheussen, Ola Lygre, Inger G. Alsos, Love Dalén, Bastiaan Star, Anne Karin Hufthammer, Thijs van Kolfschoten, Stein-Erik Lauritzen, Trond Klungseth Lødøen, Sanne Boessenkool

Corresponding authors: Samuel J. Walker, Aurélie Boilard, Sanne Boessenkool
Email: sjwalker@bournemouth.ac.uk, aurelibo@ibv.uio.no, sanne.boessenkool@ibv.uio.no

This PDF file includes:

Supporting text (Text S1–S10)
Figures S1 to S15
Tables S1 to S24
SI References

Table of contents:

Text S1. The Storsteinhola cave system	3
Figure S1	3
Figure S2	4
Text S2. The excavations	4
Arne Qvamgrotta	4
Figure S3	6
Nygrotta	6
Sampling strategy	7
Figure S4	7
Text S3. Sedimentology	7
Methods	7
Table S1	8
Sediment descriptions Arne Qvamgrotta	8
Table S2	9
Sediment descriptions Nygrotta	10
Table S3	10
Figure S5	11
Figure S6	12
Text S4. Dating	13
Optically Stimulated Luminescence (OSL)	13
Table S4	13
Dose rate	13
Table S5	14
Table S6	14
Equivalent dose determination	14
Figure S7	15
Luminescence ages: bleaching and reliability	15
Radiocarbon dating AMS ^{14}C	16
Table S7	17
U/Th dating	17
Table S8	18
Figure S8	19
Figure S9	20
Table S9	20
Phylogenetic dating	21
DNA extraction and library preparation of V. lagopus, D. torquatus and Ursus sp.	*21*
Table S10	22
Vulpes lagopus phylogenetic analyses and dating	23
Figure S10	24
Table S11	25
Dicrostonyx torquatus mitogenome reconstruction, phylogenetic analyses and dating	25
Figure S11	27
Figure S12	28
Ursus sp. phylogenetic analyses and dating	28
Table S12	29
Figure S13	30
Figure S14	31
Cosmogenic nuclide burial dating	31
Description of sample material	31
Sample preparation	32
AMS analysis	32
Results	32
Table S13	33
Text S5. Comparative osteology	33
Table S14	34
Table S15	35
Text S6. Ancient DNA bulk-bone metabarcoding	36
Laboratory methods	36
Table S16	37-38
Table S17	39
Bioinformatic analyses of BBM	40
Table S18	41
Table S19	42
Adjustments of taxonomic identification	43
Table S20	44-45
Text S7. Comparison of identification methods	45
Table S21	46-47
Text S8. Identification of shells	47
Table S22	47
Text S9. Pollen analyses	48
Table S23	48
Figure S15	49
Text S10. sedaDNA analyses	49
Table S24	50
References	50

Text S1. The Storsteinhola cave system

The Storsteinhola karst system is located in Kjøpsvik village, Municipality of Narvik in Nordland County, Northern Norway (68.10° N 16.38° E). The complete cave system has an aggregate length of ~2.6 km and a maximum vertical range of 40 m [(1, 2); Fig. S2]. In 2021 and 2022 we excavated the conduit that is now Arne Qvamgrotta and its surface opening, Nygrotta. Arne Qvamgrotta was formerly known as Norcemgrotta, but was renamed to recognize Arne Qvam, a former director of NORCEM Kjøpsvik, now Heidelberg Materials Sement Norge AS. Arne Qvam's decision to build a protective wall sealing the deposit for future research showed foresight and prevented the loss of an incredibly rare paleo-archive.

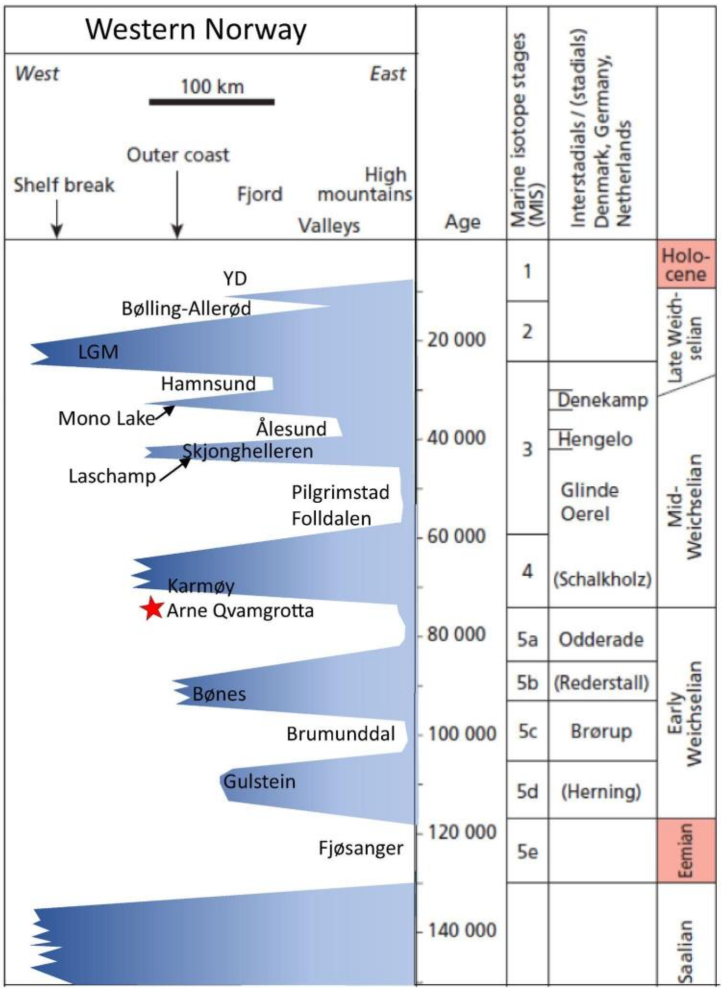

Figure S1. Glaciation curve for the southwestern edge of the Scandinavian Ice Sheet, figure adapted from Mangerud et al. (3). Names on the curve are key Norwegian sites including the site of Arne Qvamgrotta (red star), whilst the Laschamp and Mono Lake palaeomagnetic excursions are marked with an arrow, as per Mangerud et al. (3). The Younger Dryas (YD) and the Last Glacial Maximum are also marked.

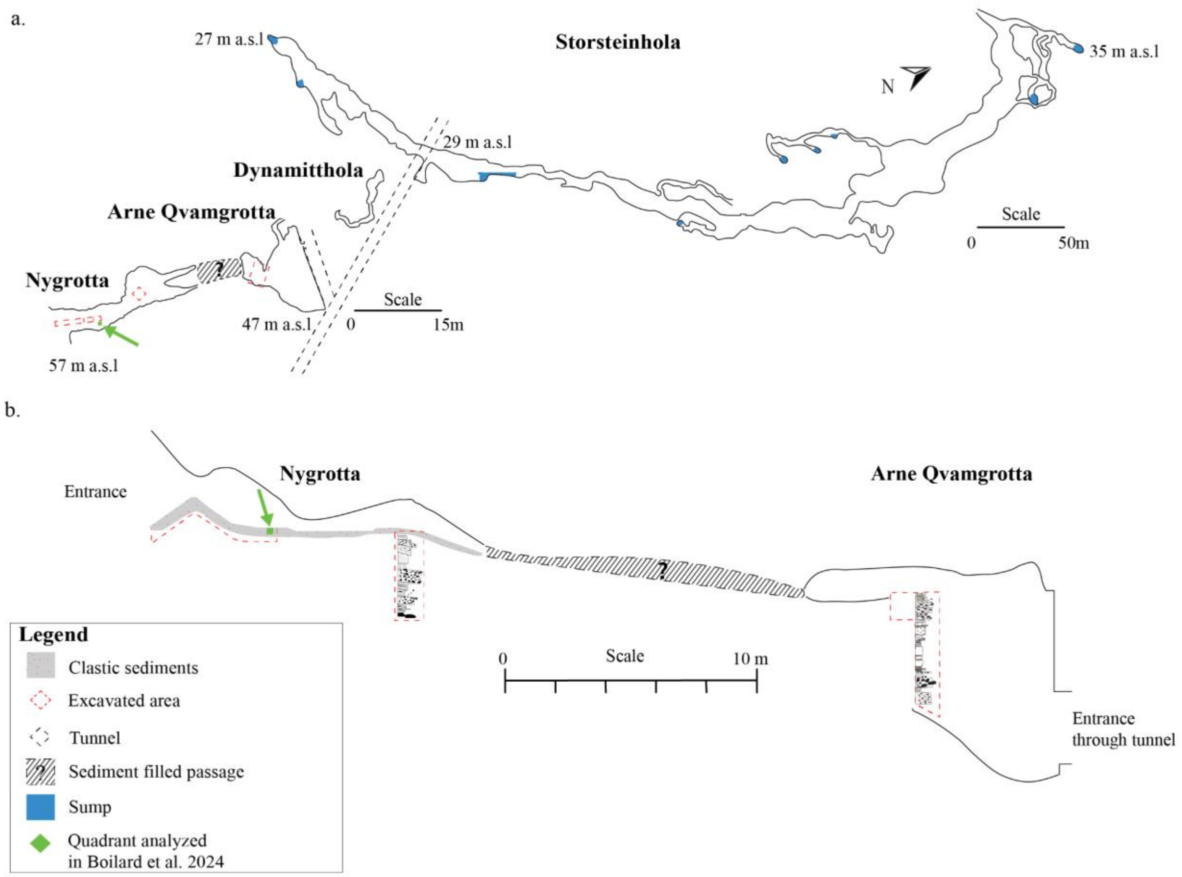

Figure S2. Plan and cross section of the Storsteinhola cave system. **a)** Plan of the Storsteinhola cave system (scale 1:50) showing the location of Arne Qvamgrotta and Nygrotta (drawn at scale 1:15 for visibility) along with height points across the cave system. Plan adapted from Lauritsen (2). **b)** Cross section showing the link between Nygrotta and Arne Qvamgrotta, which is estimated to be a 13 m filled passage. Excavations of both 2021 and 2022 in Nygrotta and Arne Qvamgrotta as well as associated logs are shown.

Text S2. The excavations

Arne Qvamgrotta

The excavation at Arne Qvamgrotta followed established archaeological excavation methodologies (4, 5). We adopted an area excavation approach to expose a vertical profile allowing a clearer understanding and interpretation of the sedimentation and stratigraphy. The profile was cut perpendicular to the cave walls to create a full and accurate cross-section of the deposit. A grid system was implemented and orientated across the sediments dividing the site into 1 m^2 units with X running north-south and Y running east-west (Fig. S3). To improve spatial resolution of the recovered material the 1 m^2 units were further divided into quadrants (NE, SE, SW and NW). We followed a stratigraphic-mechanic procedure excavating each stratigraphic layer by internal mechanical 10 cm thick units to allow for a more detailed assessment of the distribution of recovered material.

Two field seasons (September 2021 & September 2022) were conducted, excavating a total area of ~8.5 m^2 to a depth of ~5 m. During the first field season in 2021 an area of ~2.5 m^2 was excavated by hand using trowels and shovels (Fig. S3). First, one 50x50 cm column was hand excavated and sieved in its entirety at 4 and 2 mm resolution to establish which layers contained preserved

organics. Following this, and after establishing that the upper sediments were sterile, the upper ~2.5 m was shoveled and discarded and the lower sediments just above the organic layers were excavated using trowels and systematically sieved. Due to the height of the sediments, scaffolding was set up in front of the profile for security of personnel and better access and accuracy of excavation from the top while working down through the stratigraphic layers. During the second field season in 2022 we opened a larger area (~6 m^2; Fig. S3). Having established that the top ~2.5 m of sediments were sterile, we employed an industrial vacuum vehicle to rapidly remove sediments. This innovative form of excavation is ideal for fast and effective removal of sterile sediments allowing exposure of a larger area. In Arne Qvamgrotta, expanding the exposed area was important to gain a clearer understanding of the depositional processes, such as for instance the direction of water flow which is typically difficult to interpret in caves. When employing the industrial vacuum machinery, archaeologists joined the operators to ensure full control of the layers that were removed. Following the removal of the top 2.5 m across an area of 6 m^2 with the vacuum and stopping around 0.5 m above the sub-fossil bearing deposits, we continued excavating downwards by hand. Employing an industrial vacuum for removing sediments is extremely effective and without this method we would not have been able to expose such a large area within the available time frame for the excavation. Nevertheless, we do want to emphasize that such a method should only be employed when sediments have been confirmed to be sterile, as it removes the possibility for further analysis. Although it is theoretically possible to sieve sediments recovered by the vacuum, all contextual information is lost. It is also worth noting that whilst the tubing can be hundreds of meters long, this may not be long enough for very remote sites. Despite the limitations, employing an industrial vacuum opens many possibilities and could allow for more in-depth investigations when used correctly.

Sieving of excavated sediments is crucial for the recovery of small remains, such as bone, shell, seeds, charcoal and lithics (6, 7). Wet sieving was implemented at Arne Qvamgrotta which is a favorable method especially for clay or silt dominant sediments. Wet sieving was aided by direct access to a water supply and all sieving was conducted using drinking-quality tap water that has been stringently processed at the local drinking water treatment plant using both particle filtering and UV exposure. All sediments that were excavated by hand were sieved through 4 and 2 mm mesh to maximize recovery of small fragments. A subset of material was also sieved through 1 mm mesh, but as no bone remains were recovered from this additional fractionation, we limited the majority of sieving to 4 and 2 mm. Sieves and buckets were cleaned thoroughly with water and brushes between samples. The sediments and the sieved remains were inspected for archaeological artefacts but revealed no signs or traces of hominin presence. Sieved sediments were dried on geotextile in rooms set aside for sample drying. Rooms used for sample drying were cleaned thoroughly and new geotextile was used for every sample to prevent cross-sample contamination. Gloves and face masks were used during all post-sieving handling of sediments to minimize DNA contamination. Dried sediments were stored at 4°C to reduce post-excavation DNA degradation.

The recovery of organics was conducted off-site using magnification lamps. Tables were cleaned with bleach in between each sample, and sediments were placed under the lamp on clean tin foil and paper. Protective equipment (gloves and face masks) was used, and a one-way flow was applied ensuring that scientists had not been in or near modern DNA laboratories before working with the sediments. Bone and shell were recovered from the samples using tweezers, and a representative sample of the remaining material (composed of stone and mineral) was retained for each stratigraphic layer.

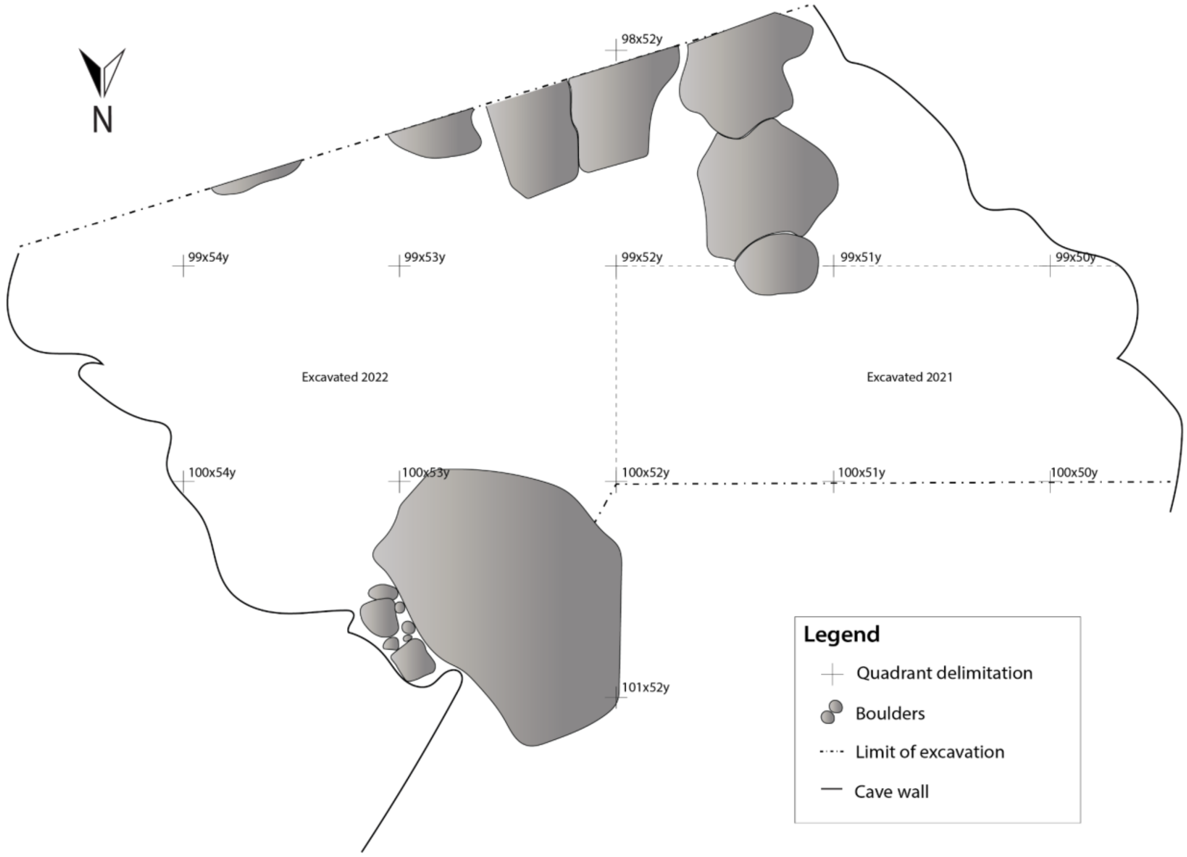

Figure S3. Plan of the excavated area at Arne Qvamgrotta. The plan shows the excavation grid system, and the areas excavated in the 2021 and 2022 field seasons. Each grid square is 1 m^2.

Nygrotta

Nygrotta was subject to two seasons of excavation, the 2021 excavation which investigated the end of the last glacial period up to the Mid-Holocene (8) and the 2022 excavation further into Nygrotta towards Arne Qvamgrotta (Fig. S2). The 2022 excavated profile in Nygrotta is the focus here, where we attempted to associate sediment layers in Nygrotta with those in Arne Qvamgrotta and obtained several Optically Stimulated Luminescence (OSL) dates from Nygrotta (see below). The excavation at Nygrotta followed the block excavation methodology, using a grid system as applied in Arne Qvamgrotta (i.e., 1 m^2 units with X running north-south and Y running east-west and a further division into quadrants). We followed the same stratigraphic-mechanical procedure as in Arne Qvamgrotta. An area of ~3.5 m^2 was opened down to a depth of ~3 m. Due to the confined space in Nygrotta, an industrial vacuum was employed to excavate an area of 3 m^2 allowing recording of the sediment deposition and sampling from the profile. A 0.5 m^2 quadrant was subsequently excavated by hand, wet sieving all material at both 4 and 2 mm resolution.

Sampling strategy

The sampling strategy at both Arne Qvamgrotta and Nygrotta was principally focused on the recovery of preserved organic material. To achieve this, large quantities of material were wet sieved and organic material was extracted by hand. In addition to the recovery of organics, bulk samples of ~1 kg from each stratigraphic layer were taken for sedimentological analysis. Further samples were taken for Optically Stimulated Luminescence (OSL), Uranium-Thorium (U/Th) and cosmogenic nuclide burial dating (Fig. S4). Samples were also taken for pollen analysis and sedimentary ancient DNA (sedaDNA; Fig. S4), whereby samples were taken from the lowest point of excavation upwards using gloves, facemasks and sterile equipment to limit contamination.

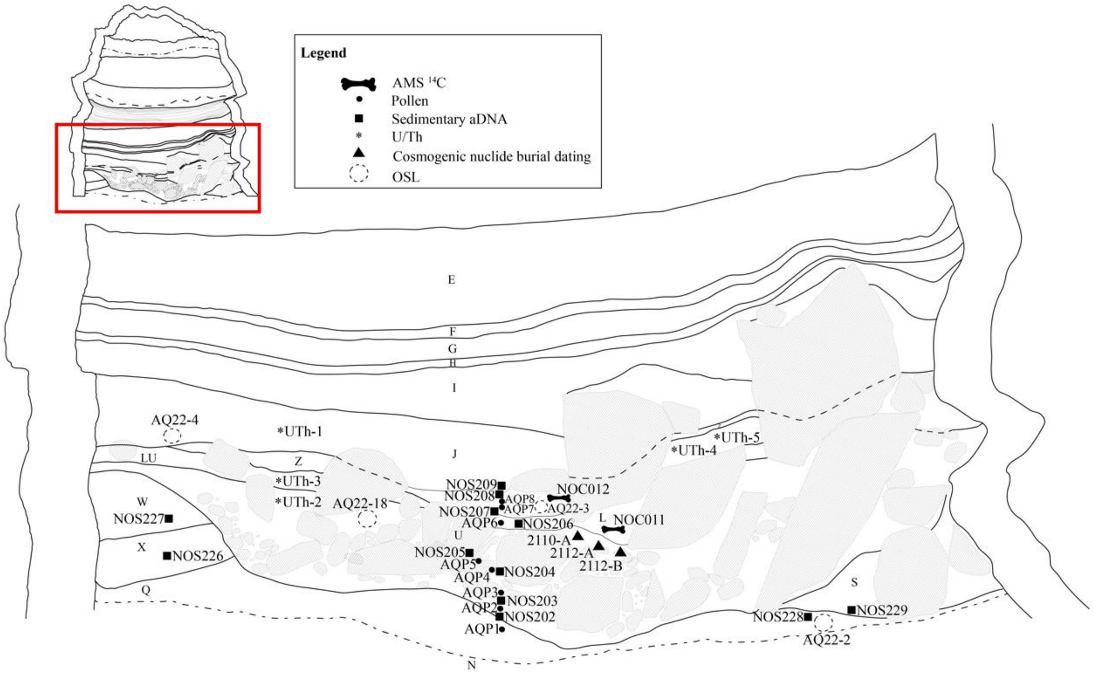

Figure S4. Arne Qvamgrotta profile drawing showing the locations of samples collected for OSL, AMS ^{14}C, $^{234}U/^{230}Th$, cosmogenic nuclide burial dating, pollen and sedaDNA analyses. Sample 2108-B for the cosmogenic nuclide burial dating was taken from Layer A, at the top of the profile. Sample information can be found in the respective sections below.

Text S3. Sedimentology

Methods

The sedimentological recording followed standard practice; after cleaning the sediment profile the sediment sections were documented and recorded through sketches, photographs and sedimentological logs using lithofacies codes adapted from Eyles et al. [(9); Fig. 2; Table S1]. Sedimentary sections were examined based on their lithology, sedimentary structures, bed contacts and clast roundness. Flow directions were obtained from imbrication and ripple laminations.

Table S1. Definition of lithofacies codes used in the sediment logs.

Grain size		Structures	
D(…)	Diamicton	m	massive
B	Boulder	s	stratified
G	Gravel	cm	clast-supported, massive
S	Sand	cs	clast-supported, stratified
Si	Silt	tc	trough cross-bedded
F	Fines	pc	planar cross-bedded
		r	ripple laminated
		l	laminated
		(def)	deformed
		(dr)	draped

Sediment descriptions Arne Qvamgrotta

In the Arne Qvamgrotta sequence we intercepted 20 stratigraphic layers that are grouped into eight larger descending units of sedimentation (Fig. 1; Fig. 2, Table S2). Unit 1 (Layer A) comprises laminated sand and gravelly sand. Unit 2 (Layer B) consists of sets of trough cross-bedded sand and gravel with an erosive lower boundary, depositing towards the southwestern side of the cave. Unit 3 (Layer C) consists of climbing ripple laminated sand interbedded with a sandy gravel layer, which is partly deformed by water escape structures and soft sediment deformation. Unit 4 comprises several layers (D, E, F, G, H, I) defined as interbedded layers of massive silty sand (E, G and I) with layers of laminated very fine sand, silt and clay (D, F and H). Concretions are found at several levels within the fine-grained sediments. Unit 5 (Layer J) consists of laminated silt and clay with some ripple laminated sand lenses. This layer drapes over Unit 6 and the protruding boulders below. Within Units 4 and 5, we observe 53 clay laminae. Unit 6 comprises four distinct discontinuous layers (L, K, Y and Z), and two interface layers (K/L and L/U) that fill the space between boulders and a concave depression in Unit 7. Layers L, K and Y are laminated silty sand capped by gravel of Layer Z. Several large angular boulders in vertical position indicate that they fell from the roof of the cave into Unit 7, especially close to the southwestern cave wall. Unit 7 is formed by one stratigraphic layer (U) defined as a diamicton with numerous sub-angular and sub-rounded boulders and cobbles, many oriented perpendicular to the cave walls and some imbricated with a tilt towards Nygrotta. Unit 7 has an erosive lower boundary cutting a channel partially through Unit 8. Unit 8 consists of five stratigraphic layers (N, Q, S, X and W) with a stratified sandy gravel (N) with gently sloping beds, overlain by planar and ripple laminated sand with some silt and clay laminae (Q), planar and ripple laminated sand (S and X) with massive sandy gravel (W). Layer S, Y, X, and W are remnant deposits only found at the cave walls.

Table S2. Sediment information per stratigraphic layer in Arne Qvamgrotta. For lithofacies codes, see Table S1.

Unit	Layer	Depth (cm)	Lithofacies code	Description
1	**A**	0–40	Sl, Sr<br>GSs	Uppermost flat-lying oxidized beds of coarse and medium sand and gravelly sand with scattered cobbles.
2	**B**	40–95	SGtc<br>GSs	Sets of sandy gravel trough cross-bedded and stratified gravelly sand with some cobbles (<20 cm in size). Sets are around 25 cm thick. Wedges out towards the left. Erosive lower boundary.
3	**C**	95–200	Sl, Sr<br>Sr(def)	Planar parallel and ripple-laminated medium sand, partly climbing ripples. Includes some silty sand laminae and gravelly layers. Synsedimentary deformation structures with ball-and-pillow and small water escape structures.
	**D**	200–255	Sl, Sr<br>Fl	Horizontally laminated very fine sand, silt and clay, normal graded. Some few ripple laminae and small cut-and-fill structures. Concretions are present in the clay laminae.
	**E**	255–295	SiSm	Massive silty very fine sand.
	**F**	295–305	Fl	Horizontally laminated clay, silt and very fine sand.
4	**G**	305–315	SiSm	Massive silty very fine sand.
	**H**	315–320	Fl	Horizontally laminated clay, silt and very fine sand. Concretions present.
	**I**	320–350	SiSm	Massive silty very fine sand.
5	**J**	95–200	Fl(dr)<br>SiSr(dr)	Laminated sand, clay and mostly silt, draping. Some silty sand layers are ripple laminated. Concretions are present in the fine-grained laminae.
	**Z**	-	SG	Sandy gravel, sub-angular clasts. Bones present.
	**Y**	-	SiSl	Laminated silty sand. Bones present. Likely the same stratigraphic layer as K. Right hand side remnant.
6	**K**	375–385	SiSl(dr)	Laminated silty sand, draping. Bones present. Concretions present.
	**L**	385–405	SiSl(dr)	Laminated silty sand with scattered gravel. Some variation in the amounts of gravel and silt across the layer. Bones present.
7	**U**	405–450	D(G)cm	Massive clast-supported diamicton with matrix of silty sandy gravel. Many cobbles and boulders between 10–50 cm. Some boulders show imbrication suggesting a flow into the cave from Nygrotta. Lower boundary is channel-shaped and erosional. Some bones present in the upper mechanical layers.
	**W**	-	SGm	Massive sandy gravel. Remnant left in left corner.
	**X**	-	Sl, Sr	Planar parallel and rippled laminated sand. Remnant left in left corner.
	**S**	-	Sl	Laminated fine and coarse sand. Likely same stratigraphic layer as X. Right hand side remnant.
8	**Q**	450–470	Sl, Sr<br>Fl	Ripple and planar parallel laminated sand with some variation across the layer. Silty sand with clay laminae in the lower part, coarsening upward. Few bones present.
	**N**	>470	GSs	Stratified sand and gravel, rounded to angular clasts, gently sloping beds (thickness >60 cm).

Sediment descriptions Nygrotta

In the Nygrotta sequence we intercepted 10 stratigraphic layers that are grouped into five descending units of sedimentation (Table S3; Fig. S5). Unit N1 consists of flat-lying beds of massive (Layer NA) and laminated (Layer NB) sand. Unit N2 comprises three layers, where the upper trough cross-bedded sandy gravel of Layer NC is overlying cross and horizontal bedded (gravelly) sand (Layer ND) which is overlying a draping ripple laminated sand layer (NE). Below is Unit N3, consisting of two Layers, NF and NG. Layer NF comprises stratified clast-supported gravel and cobbles with a silty sandy matrix. It is eroding into Layer NG consisting of cross-bedded, planar and ripple laminated sand. Some clay laminae occur in the lower part. Unit N4 also comprises two layers, where the upper Layer NH contains massive sandy gravel with cobbles where the matrix varies from silty sand to clay. Below an erosional contact is Layer NI. It consists of dipping layers and laminae of sand, silt and clay, including some ripple lamination. Layer NJ is the only layer within Unit N5, where gravelly silt and sand is found in between clast-supported boulders. Units N2, N3 and N4 are all coarsening upwards. The sediments are interpreted as glaciofluvial with flow towards the Nygrotta entrance (Fig. S2).

Three possible scenarios were identified for the correspondence between the excavated layers in Arne Qvamgrotta to those in Nygrotta (Fig. S6). First, layer B may correlate with the upper cross-bedded gravel and sand Layers of NC-ND. Sediments at both sites are likely glaciofluvial bar deposits. Second, Layer B may correlate with all the gravelly Layers NC-NK. Third, the glaciofluvial Layers B and C may correlate with the sandy and gravelly Layers NC-NK. Based on the current data we cannot exclude any of these scenarios, but neither of them affects our interpretation of the lower layers and specifically not the bone-bearing layers in Arne Qvamgrotta.

Table S3. Sediment information per stratigraphic layer in Nygrotta. For lithofacies codes, see Table S1.

Unit	Layer	Depth (cm)	Lithofacies code	Description
N1	**NA**	0–8	Sm	Massive fine sand.
	**NB**	8–22	Sl	Laminated fine and medium sand.
N2	**NC**	22–40	SGtc	Sets with trough cross-bedded sandy gravel with clasts up to 9 cm. Dip toward southeast (cave entrance). Partly erosive lower boundary.
	**ND**	40–106	GSpc<br>Ss	Planar cross-bedded gravelly sand with dip toward southeast and horizontal stratified medium and coarse sand.
	**NE**	106–126	Sr(dr)	Ripple laminated fine sand, draping. Some climbing ripples and draping silt laminae.
N3	**NF**	126–168	D(SG)cs	Clast-supported stratified gravel and cobbles (up to 18 cm) with silty sandy matrix. Matrix more clayey in middle part and sandier in upper part. Erosive lower boundary. Dip towards the southeast.
	**NG**	168–220	Spc<br>Sl, Sr	Sets of planar cross-laminated sand in the upper right corner, cutting underlying horizontal laminated sand. The lower part includes ripple laminae and some few clay laminae and some scattered pebbles. Dip towards the southeast.
N4	**NH**	220–246	SGm	Massive sandy gravel with cobbles in the upper 10 cm. In the middle part the clasts have a coating of silt. Wedges out towards the left.
	**NI**	246–278	SSil<br>Fl	Interbedded layers of sand, silty sand and silt. Some ripple laminae and clay laminae. Dip towards the south.
N5	**NJ**	278–300	SiBcm	Clast-supported boulders (>35 cm) with matrix of silt and gravelly silt and sand.

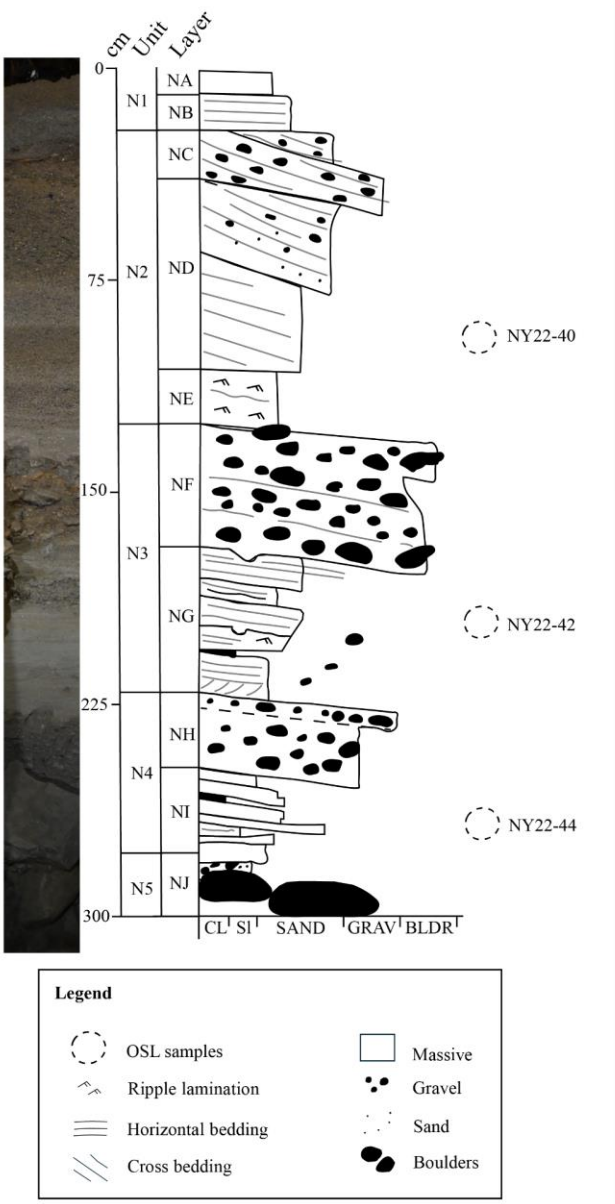

Figure S5. Sediment log of Nygrotta. Photo of the profile (left) and log (right) of the excavated area in Nygrotta. Sampling depth of analyzed OSL samples are shown (NY22-40, NY22-42, NY22-44).

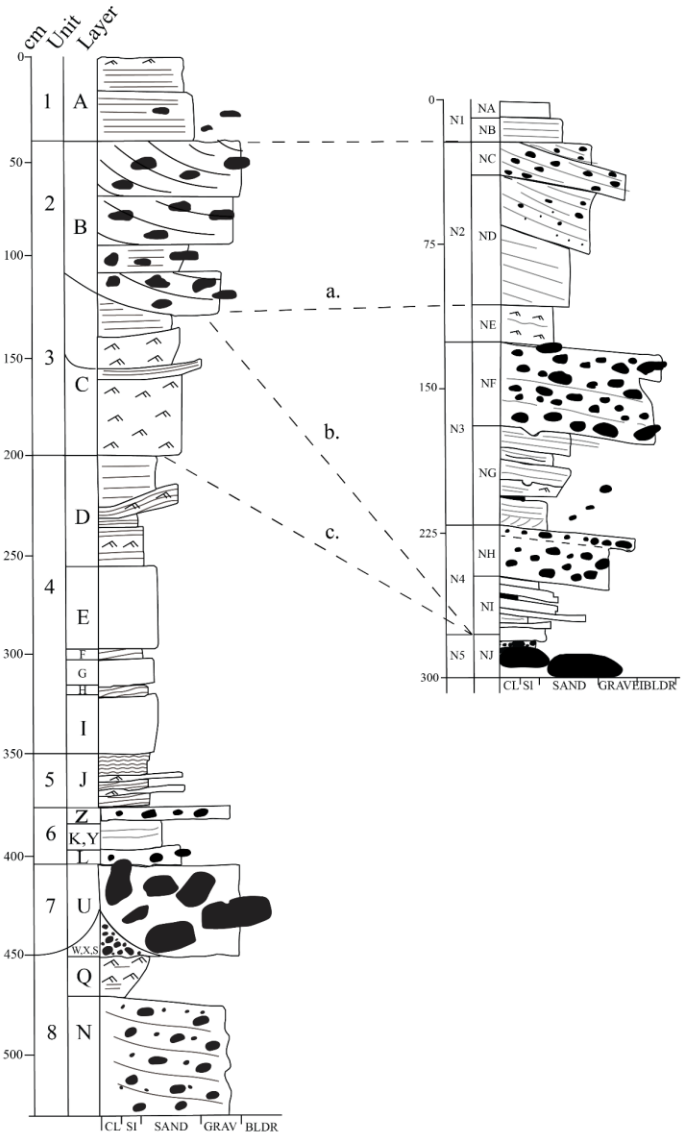

Figure S6. Correspondence between Arne Qvamgrotta and Nygrotta sediments. Three possible scenarios were identified: a. Layer B correlates with the upper cross-bedded gravelly and sandy layers of NC-ND; b. Layer B correlates with all the gravelly Layers NC-NI; c. The glaciofluvial Layers B and C correlate with the sandy and gravelly Layers NC-NI. CL = clay, SI = silt, GRAV = gravel, BLDR = boulder.

Text S4. Dating

Optically Stimulated Luminescence (OSL)

Samples for Optically Stimulated Luminescence (OSL) dating were selected from distinct sand-rich layers in Arne Qvamgrotta (four samples from Layers N, K, Z and U; Fig. S4; Table S4) and Nygrotta (three samples from Layers ND, NG and NI; Fig. S5; Table S4). Samples were collected by hammering steel or plastic tubes into freshly cleaned sediment surfaces, with the exception of sample AQ22-18 for which sediment was collected in a hollow steel rod and then transferred to a plastic tube as the amount of rocks did not allow hammering a plastic tube in the sediment surface. Background radiation was measured in the field using a Radiacmeter 6150AD. Tubes were transported to the Nordic Laboratory for Luminescence Dating (DTU Physics, Roskilde), where they were opened under subdued orange lighting conditions (10). The potentially light-exposed outer ~5 cm of material at both tube ends was used for dose rate analysis whereas the inner material was used for equivalent dose determination and water content measurements.

Table S4. OSL sample overview. OSL sample number, OSL lab ID from the Nordic Laboratory for Luminescence Dating (DTU Physics, Roskilde), cave and the corresponding stratigraphic layer, OSL Burial depth, water content (% water/dry weight) and background radiation are presented.

Sample No.	OSL lab ID	Cave	Layer	Depth (meters)	w.c. (%)	Background (µGr/h)
NY22-40	22 09 01	Nygrotta	ND	0.80	22	0.063
NY22-42	22 09 02	Nygrotta	NG	1.85	23	0.060
NY22-44	22 09 03	Nygrotta	NI	2.55	18	0.073
AQ22-4	22 09 06	Arne Qvamgrotta	Z	3.80	18	0.061
AQ22-3	22 09 05	Arne Qvamgrotta	K	4.00	21	0.054
AQ22-18	22 09 07	Arne Qvamgrotta	U	4.30	12	NA
AQ22-2	22 09 04	Arne Qvamgrotta	N	4.50	29	0.079

Dose rate

Dose rates were determined using high resolution gamma spectrometry (11). For this analysis the outer ~5 cm of samples was first dried and then homogenized by grinding to a fine powder (<200 µm). This powder was mixed with hot bottle wax and cast in a cup-shaped mould. Casting in wax minimizes ^{222}Rn and gives a fixed counting geometry. After three weeks of storage to allow equilibrium to establish between ^{222}Rn and the parent ^{226}Ra, the cups were counted for at least 24 hours on a high-resolution gamma spectrometer that was calibrated following Murray et al. (11, 12). The resulting radionuclide concentrations are given in Table S5. Radionuclide concentrations were converted to dose rates using the conversion factors of Creswell et al. [(13); Table S5]. An internal beta dose rate component to 180–250 µm K-feldspar grains was calculated assuming a 12.5 ± 0.5% K concentration and a 400 ± 100 ppm Rb concentration (14, 15). The dose rate contribution coming from internal U and Th was assumed to be 0.10 ± 0.05 Gy/ka (16). A cosmic ray component was incorporated following Prescott and Hutton (17). We measured both ‘present day’ water content from inner tube material and saturated water content using the syringe method (18). For life-time average water content we assumed 50% of the saturation water content which is, for four out of seven samples, in good agreement with the measured present day water contents. For the three samples with present day water content lower than 50% saturation, the sampling tubes were probably not completely sealed, and the samples dried out a little. Total feldspar dose rates ranged between ~2.5 and ~3.7 Gy/ka (Table S6).

Table S5. Radionuclide concentrations and dry, infinite matrix beta and gamma dose rates for the OSL samples.

Sample No.	^{238}U Bq.kg^{-1}	^{226}Ra Bq.kg^{-1}	^{232}Th Bq.kg^{-1}	^{40}K Bq.kg^{-1}	beta dose rate Gy.ka^{-1}	gamma dose rate Gy.ka^{-1}
NY22-40	17 ± 6	19.1 ± 0.5	19.4 ± 0.5	301 ± 8	1.13 ± 0.03	0.60 ± 0.02
NY22-42	27 ± 4	38.0 ± 0.5	41.8 ± 0.4	601 ± 9	2.28 ± 0.04	1.24 ± 0.03
NY22-44	16 ± 2	20.3 ± 0.3	24.4 ± 0.3	446 ± 6	1.57 ± 0.02	0.78 ± 0.02
AQ22-4	21 ± 6	21.4 ± 0.5	28.0 ± 0.5	422 ± 8	1.54 ± 0.03	0.82 ± 0.02
AQ22-3	23 ± 2	26.0 ± 0.2	33.3 ± 0.2	459 ± 5	1.72 ± 0.02	0.94 ± 0.02
AQ22-18	22 ± 9	20.7 ± 0.7	32.4 ± 0.8	401 ± 15	1.51 ± 0.04	0.85 ± 0.02
AQ22-2	43 ± 7	34.3 ± 0.6	56.3 ± 0.7	454 ± 9	1.95 ± 0.03	1.27 ± 0.03

Table S6. IR_{50} and $pIRIR_{290}$ equivalent doses and number of accepted (n_a) and rejected (n_r) aliquots [following IQR criterion of Tukey, (19)]. Total feldspar dose rate, resulting IR_{50} and $pIRIR_{290}$ ages. The pIRIR290 ages with residual correction have a residual dose of 339 ± 36 Gy subtracted prior to age calculation. Ages have not been corrected for any potential signal instability. Minimum ages are given based on 2,5xD_c for sample 220904 because natural $pIRIR_{290}$ signals were in full saturation. Ages in bold are the preferred ages for interpretation. Uncertainties are all one sigma. Total and random uncertainties on the $pIRIR_{290}$ age are given in normal and small font size, respectively.

Sample No.	IR_{50} D_e (Gy)	(n_r)	(n_a)	pIRIR D_e (Gy)	(n_r)	(n_a)	Feldspar dose rate (Gy/ka)	IR_{50} age (ka)	$pIRIR_{290}$ age (ka)		pIRIR290 age, resid subtr. (ka)	
NY22-40	262 ± 7	2	7	596 ± 49	0	9	2.47 ± 0.09	106 ± 5	241 ± 22	20	104 ± 25	20
NY22-42	192 ± 8	1	8	534 ± 49	0	9	3.72 ± 0.13	52 ± 3	143 ± 14	13	52 ± 16	13
NY22-44	166 ± 13	0	9	484 ± 43	1	8	2.95 ± 0.11	56 ± 5	164 ± 16	15	49 ± 19	15
AQ22-4	328 ± 10	1	8	602 ± 25	0	9	2.94 ± 0.11	112 ± 5	205 ± 12	9	90 ± 15	9
AQ22-3	403 ± 24	0	9	716 ± 66	0	9	3.10 ± 0.11	130 ± 9	231 ± 23	21	122 ± 25	21
AQ22-18	386 ± 25	0	9	725 ± 57	1	8	3.05 ± 0.12	127 ± 10	238 ± 21	19	127 ± 23	19
AQ22-2	>450	0	9	>900	0	9	3.35 ± 0.12	>130	>270		>270	

Equivalent dose determination

The inner material of collection tubes was processed using conventional sample preparation techniques as described in Murray et al. (18). A sufficient quantity of quartz and K-feldspar grains in the 180–250 µm fraction could be extracted from all samples. Large (8 mm) and small (2 mm) aliquots of quartz and K-rich feldspar were mounted on stainless cups using silicon spray as an adhesive. All measurements were done with standard Risø TL-OSL readers (model DA-20) equipped with blue LEDs (470 nm), infrared (IR) LEDs (850 nm) and beta sources calibrated using Risø calibration quartz (20, 21).

The purity of the quartz was confirmed by the absence of a significant infrared stimulated luminescence (IRSL) signal and an OSL IR depletion test (22). These analyses showed that the quartz OSL signal from all samples displayed undesirable characteristics: the signal was dim, and a fast component was absent. As a fast OSL component is required for reliable determination using quartz (18, 23) this was not considered further.

A post-IR IRSL protocol (24) was used to measure the feldspar De values. We employed the SAR $pIRIR_{50,290}$ protocol which has been tested by Buylaert et al. (25) and is often used in luminescence dating studies. Natural and regenerative dose signals were measured after a preheat of 320°C for 60 s by stimulating with infrared (IR) light for 200 s whilst holding the grains at 50°C. The resulting

infra-red stimulated luminescence (IRSL) signal is called the IR_{50} signal. Subsequently, the feldspar grains were stimulated again for 200 s, but now at 290°C to measure the more stable $pIRIR_{290}$ signal. Test dose signals were measured in the same way and the SAR cycle was completed by an elevated temperature (325°C) IR-cleanout for 200 s to minimize recuperation. The test dose size was typically 30–50% of the measured dose. The initial 2 s of the stimulation curve minus a background derived from the last 50 s was used for all calculations. As is common with feldspar, both the IR_{50} and $pIRIR_{290}$ signals are sensitive; an example of a $pIRIR_{290}$ signal is given in the inset of Fig. S7a.

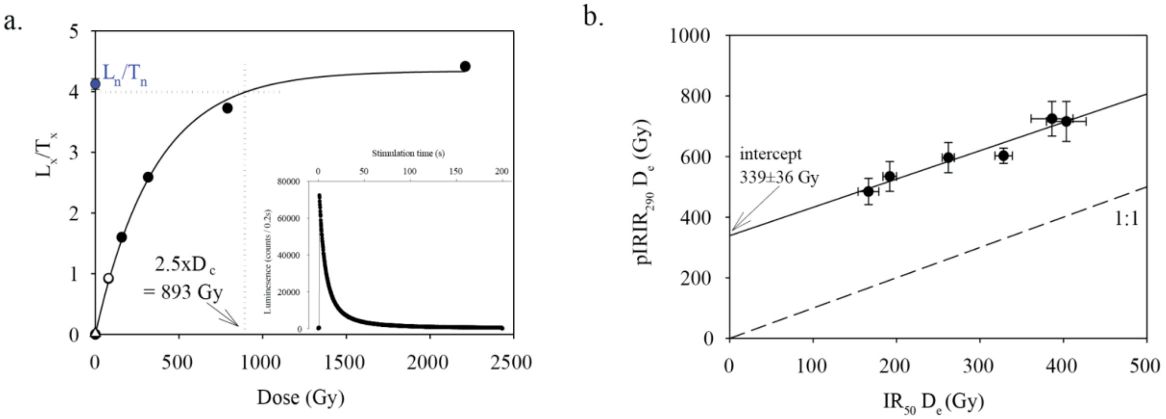

Figure S7. OSL dose and IR **a)** Felspar $pIRIR_{290}$ dose response curve for an aliquot of sample 220904 with natural signal (blue), recycled point (open circle) and recuperation (open triangle) shown. Dotted lines show the intersection at 92% of saturation and dose ($2xD_c$) corresponding to this level. Inset gives the natural stimulation curve. **b)** $pIRIR_{290}$ D_e values plotted as a function of IR_{50} D_e values (excluding saturated sample 220904). The solid line is a linear fit through the data with slope 0.93 ± 0.11 and intercept 339 ± 36 Gy.

We carried out a dose recovery test by adding a dose of 150 Gy on top of a set of pristine aliquots of sample 220903. After subtraction of the $pIRIR_{290}$ D_e from the measured dose, a dose recovery ratio of 0.92 ± 0.19 (n=6) was calculated. The measured standard error on this otherwise acceptable dose recovery ratio (within 10% of unity) is rather large because of scatter in the measured recovered doses.

The IR_{50} and $pIRIR_{290}$ D_e values listed are given in Table S6. Six out of seven samples gave finite D_e values. The natural $pIRIR_{290}$ signal of sample 220904 was too close to the saturation level of the dose response curve to allow reliable interpolation (Fig. S7a). A minimum dose estimate for this sample is calculated. This is based on the characteristic dose (D_c) derived from fitting the dose response curve with a single saturating exponential function of the form $I=I_0 \times (1-exp(D/D_c)$, where I is the sensitivity corrected luminescence (L_x/T_x), I_0 is the saturation level of the dose response curve and D is the dose. We quote a minimum age for this sample based on a dose equal to 2.5 x D_c (signal at 92% of saturation level), i.e. 889 ± 29 Gy (n=6) (rounded to >900 Gy in Table S6). The other samples have $pIRIR_{290}$ D_e values in the range 500–700 Gy and these are used to calculate finite ages.

Luminescence ages: bleaching and reliability

The resulting IR_{50} and $pIRIR_{290}$ luminescence ages are given in Table S6 and shown as filled symbols in Fig. 3a. Except for the top sample (220901) the results are in good stratigraphic order. If one excludes the top sample, extrapolation to zero depth of the $pIRIR_{290}$ ages would lead to a significant intercept on the age axis somewhere between 50 and 100 ka. This may suggest that the $pIRIR_{290}$ signal was not completely reset to negligible levels at time of deposition in the cave.

The $pIRIR_{290}$ signal is much more stable than the IR_{50} signal but is also more difficult to reset using daylight and is thus more prone to suffer from residual signals at deposition (25). The size of this

residual dose can be estimated by comparing the $pIRIR_{290}$ (and IR_{50}) ages with the much more bleachable quartz OSL for a suite of young samples [e.g., (26, 27)]. For well-bleached samples such as in loess a typical offset is calculated to ~10 Gy. Alternatively, one can estimate the residual $pIRIR_{290}$ dose by measuring modern analogue samples. For less favorable environments, Murray et al. (28) reported residual doses of the order of ~30 Gy for glaciofluvial sediments from Svalbard (data from; (29) whereas other modern analogues showed much smaller residuals. Lowick et al. (30) present $pIRIR_{290}$ ages from water lain sediments in Switzerland that by comparison with independent age control indicate residual $pIRIR_{290}$ doses at deposition that are much larger (several tens to hundreds of Gy) than those reported by Murray et al. (28). Unfortunately, modern analogue samples do not exist for our site and the poor quartz OSL characteristics prevent a comparison of the IR_{50} and $pIRIR_{290}$ feldspar ages with quartz OSL ages.

Buylaert et al. (31) used the relationship between the $pIRIR_{290}$ and IR_{50} D_e values to detect samples with poor bleaching of the $pIRIR_{290}$ signal (their Fig. 7b). It is expected that samples that are sufficiently bleached for both IR_{50} and $pIRIR_{290}$ signals plot along a smooth curve passing through the origin; this can be seen in the data from Buylaert et al. (31). However, if the $pIRIR_{290}$ signal is not as well-bleached as the IR_{50} signal an intercept will be detectable; this is the case for our suite of samples (Fig. S7b). This intercept (339 ± 36 Gy) can be interpreted as the residual $pIRIR_{290}$ dose present at time of deposition for our samples. We have subtracted this offset from the measured $pIRIR_{290}$ doses to give residual-corrected $pIRIR_{290}$ ages (Table S6; open symbols Fig. 3a). These residual-corrected ages are presumably closer to the true deposition age of the sediment (except top sample 220901 which is discussed below).

A clear linear relationship between the $pIRIR_{290}$ and IR_{50} data would imply that this large residual offset has remained constant over at least ~60 ka (difference between the bottom and top age groups, excluding 220901); this is surprising because bleaching is a heterogeneous process, and one would thus expect that sediment bleached to various degrees would be present in the sediment sequence. The top sample (220901) is, despite residual subtraction, still clearly out of stratigraphic order and we interpret this sample to be the result of reworking of old cave sediment inside the cave. We also note that the age-depth relationship of the residual-corrected $pIRIR_{290}$ ages is now more consistent with an intercept close to the origin (~modern age at zero depth).

Radiocarbon dating AMS ^{14}C

Seven bone specimens were radiocarbon dated at the Oxford Radiocarbon Accelerator Unit (ORAU; Table S7). Five of these specimens were excavated in 1993 and have since been stored at the University Museum of Bergen, while two specimens were excavated in 2021 and 2022, respectively. The associated layers in which the 1993 specimens were recovered are not clear with some bones found in the spoil below the profile, and therefore their exact location cannot be determined. The two specimens from 2021 (OxA-42509) and 2022 (OxA-43285) were found in Layers L and K respectively, which are the main bone bearing layers in Arne Qvamgrotta.

All radiocarbon dates were calibrated using OxCal version 4.4.4 software (32). For samples which had a $\delta^{13}C$ value indicating a terrestrial diet (OxA-42509) the calibration curve IntCal20 for the Northern Hemisphere (33) was used. For samples indicating a marine diet (OxA-42506, OxA-42507, OxA-42505 and OxA-43285) the marine calibration curve Marine20 (34), with an ΔR offset of -100 ± 37 (35) taken from Tromsø, Norway (the closest data point to the site) was used. The calibrated age ranges reported are at the 95.4% probability standard.

Samples P-52086 and P-52089 failed due to low yield and no yield, respectively. Four samples (OxA-42505, OxA-42507, OxA-42509 and OxA-43285) returned minimum ages beyond the limit of radiocarbon dating. One sample (OxA-42506) returned a radiocarbon age of 41,403–39,046 ka (Table S7). However, given that all other dates have returned non-finite ages, along with the results of the OSL, U/Th and the phylogenetic dating (see below) we believe this specimen is also likely beyond the radiocarbon limit, the younger age possibly a result of contamination with modern (younger) carbon. The most reliable dates come from the specimens recently excavated from

secure contexts (OxA-42509 and OxA-43285), both of which return non-finite dates for the bone bearing horizon.

Table S7. Radiocarbon dates for Arne Qvamgrotta. Specimen information and data for specimens that were radiocarbon dated at the Oxford Radiocarbon Accelerator Unit (ORAU) in Oxford. Specimens P-52086 and P-52089 failed due to low yield and no yield, respectively. aDNA Lab ID refers to the sample identifier in the aDNA laboratory at the University of Oslo. UMB ID refers to the specimen identifier in the collection at the University Museum of Bergen. Layer information includes a number referring to the mechanical layer within the stratified layer. Uncalibrated radiocarbon ages and calibrated ages at 95.4% probability in years before present (BP). Radiocarbon dates were calibrated using OxCal version 4.4.4 software (32). Samples indicating a primarily terrestrial diet (OxA-42509) were calibrated with IntCal20 for the Northern Hemisphere (33). Samples indicating a marine diet (OxA-42506, OxA-42507, OxA-42505 and OxA-43285) were calibrated with Marine20 (34), with an ΔR offset of -100 ± 37 (35) taken from Tromsø, Norway [the closest data point to the site; (36)].

ORAU Lab ID	aDNA Lab ID	UMB ID	Species	Element	Layer	Radiocarbon Age (BP)	Collagen yield (%Yld)	Calibrated date at 95.4% probability (BP)	$\delta^{13}C$	C:N	Year excavated
P-52086	NOC001	NA	*Ursus* sp.	Distal phalanx	Exact layer unknown	Failed	-	NA	-	-	1993 (1, 37)
P-52089	NOC004	ID24, JS826	*Ursus* sp.	Ulna	Associated to Layer B	Failed	-	NA	-	-	1993 (1, 37)
OxA-42506	NOC003	ID9, JS826	*Ursus* sp.	Vertebra	Exact layer unknown	36,000 ± 710	5.1	41,403–39,046	-15.3	3.355	1993 (1, 37)
OxA-42507	NOC005	ID2, JS1733	*C. lupus*	Ulna	Exact layer unknown	>43,700	1.1	Out of range	-16.9	3.255	1993 (1, 37)
OxA-42505	NOC002	ID1, JS826	*Ursus* sp.	Rib	Exact layer unknown	>52,600	2.9	Out of range	-14.8	3.2	1993 (1, 37)
OxA-42509	NOC011	JS1869, B-GR-OU3007*	*Ursus* sp.	Tibia	L2	>55,400	1.2	Out of range	-17.1	3.162	2021
OxA-43285	NOC012	B-S-OK1002*	*Ursus* sp.	Vertebra	K1	>51,100	2.4	Out of range	-14.9	3.2105	2022

* Unique specimen identifier given to all excavated bone material from the 2021 and 2022 excavations.

U/Th dating

Calcareous concretions in cave sediment [Höhlenkrapfen, *sensu*; Kyrle, (38)] consist of concentric cemented nodules in sandy, silty or clayey sediments. The carbonate content of the cement is usually less than ~10% and their chronology poses several challenges. Firstly, such concretions typically form after sediment deposition and therefore post-date the deposit. Secondly, precipitation of calcite from the pore-water may occur at any time after, so that the chronology need not be in stratigraphic order, implying that the oldest date at the lowest stratigraphic position provides a minimum age for the particular stratum. In a glacial environment, like in Arne Qvamgrotta, it is likely that supersaturation and triggering of the cementation process may occur when cold, sub-glacially derived pore-water is slightly warmed during the transition from stadial to interstadial/interglacial. Calculations using the approach described by Dreybrodt (39) suggest that a warming of 1–2 °C

would be sufficient to make previously saturated pore-water sufficiently supersaturated. The fact that the dates of such concretions tend to cluster into periods of cold/warm transitions supports this theory (40–42).

The extremely high detrital content of Höhlenkrapfen poses methodological challenges when applying Uranium-series dating on the calcite. This is due to detrital contamination with non-authigenic ^{230}Th which makes the age appear too old. Dating 'dirty calcites' can be achieved using a regression technique (isochron plotting), where coeval subsamples with different degrees of contamination are analyzed to calculate a 'contamination-free' endmember (43–46). To obtain coeval subsamples with varying degrees of contamination, a thorough observation of the samples usually allows the identification of 'cleaner' calcite areas. However, the concretions in our study are quite homogeneous prompting a sequential leaching approach, where sample powders are leached with increasing acid concentration as the stronger acids will extract more detrital contaminant.

To obtain the necessary spread of isotope ratios for well-defined isochrons, leaching experiments were performed on the collected carbonate powders. Separate subsamples of three carbonates (UTh 1–3; Table S8) were treated with weak (1 mol/L) acetic acid, 1 M and 6 M HCl for 1 hr each at room temperature, and finally an independent subsample was totally dissolved in 15 M HNO_3 at elevated temperature. Pretreatments of two additional samples (UTh 4–5; Table S8) differed from the previous leaching experiment. These latter samples were fired at 900 °C in a muffle furnace overnight to convert $CaCO_3$ to CaO and ensure destruction of organic matter (44, 47, 48). Subsamples of fired carbonate powders were then sequentially leached, first in 0.1 M HCl at room temperature for 1 hr, then spiked (^{229}Th-^{233}U-^{236}U) and the supernatant was recovered in a centrifuge after washing the residue with ultrapure water. As a second step, the residue was fully dissolved in concentrated HNO_3. The samples were ^{229}Th-^{233}U-^{236}U-spiked based on prior measurements of U and Th concentrations using an Agilent 8800 ICP-MS.

Table S8. Concretions analyzed for U/Th. Layer includes a number referring to the mechanical layer within the stratified layer. Weight presents the amount of carbonate material that was analyzed.

Sample	Layer	Quadrant	Size (µm)	Weight (g)
UTh-1	J	99x51y NE	125–500	2.7369
			<125	3.3007
UTh-2	U1/2	99x51y NE	125–500	5.8080
			<125	3.1450
UTh-3	L/U	99x49y NE	125–500	4.0025
			<125	2.2843
UTh-4	K1	99x51y NE	125–500	4.2809
			<125	4.9481
UTh-5	K1	99x52y NE	125–500	1.4377
			<125	1.2493

The column chemistry procedures were performed in a clean laboratory environment at the HUN-REN Institute for Nuclear Research, Debrecen, Hungary. Pre-cleaned (1:1 aqua regia:ultraclean water) columns were filled with UTEVA® resin, pre-suspended in Milli-Q (MQ) water and the U-Th separations were performed using a combination of HNO_3, HCl and $HClO_4$ of different molarities following procedures of Horwitz et al. (49) and La Rosa et al. (50), modified by Surányi (51). Finally, the samples were taken up in 3% nitric acid for the isotopic analysis. Mass spectrometry was performed using a Neptune Plus MC-ICP-MS at the same institute in dry plasma mode using an Aridus 3 desolvation nebulizer. During the isotopic measurements, the so-called StalMix solution,

containing both the ^{229}Th-^{233}U-^{236}U-spike and the dissolved CRM 112-A reference material, was used as standard and for bracketing samples with unknown U-Th isotopic compositions. Mass bias corrections were made using the known ^{235}U/^{238}U isotopic ratio of the respective sample. Activity ratio and age errors are reported as 2σ values. Calculations and illustrations were done using IsoplotR online (52).

Uranium (^{238}U) concentrations of the analyzed cave carbonates were relatively low ranging between 35 and 406 ppb. The ^{230}Th/^{232}Th activity ratios of the samples were found to be in the range of 0.463 – 2.122 indicating significant detrital contaminations. Carbonates with ^{230}Th/^{232}Th activity ratios below 20 (44), sometimes <100 (53), are considered impure carbonates which require correction for non-zero initial ^{230}Th/^{238}U.

A previous sample series collected in the 1990s was leached with the weak acetic acid approach and dated using alpha-spectrometry (1, 37). We re-analyzed the data from one sample from this previously collected series (sample E1-1) in addition to analyzing the new sample series. All samples were first scrutinized for sufficient spread along the line (cases where the slope was basically defined by one "outlier" point was rejected). This rendered only two samples as reliable (Fig. S8: Fig. S9; Table S9), with one from the previous sample series and one from the new sample series both collected from Layer J in Arne Qvamgrotta. The alpha-spectrometry of sample E1_1 was dated to 71.0 ± 9.0 ka, while sample UTh-1 was dated to 31.4 ± 14.8 ka.

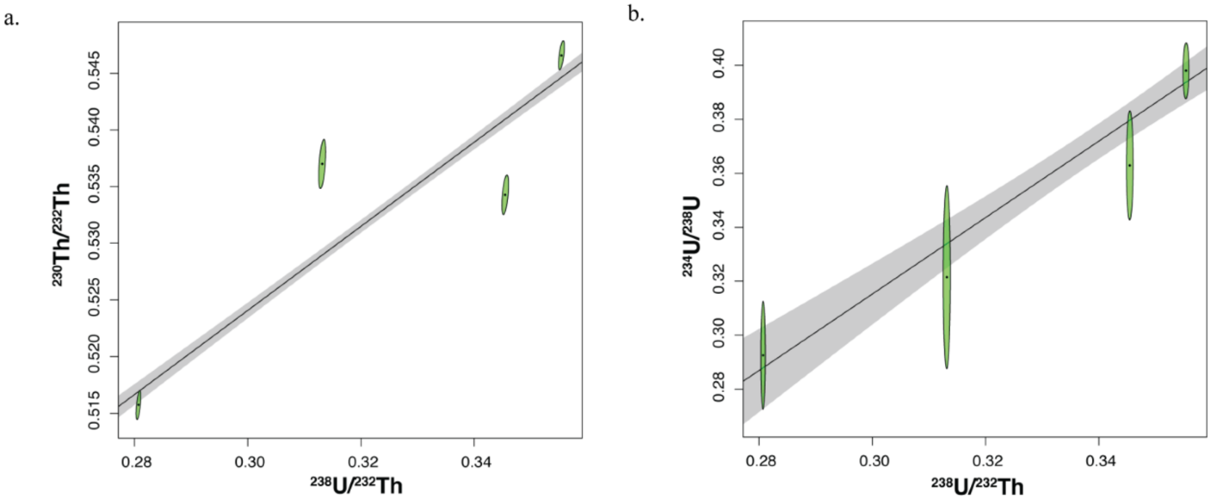

Figure S8. Rosholt type-II isochron diagrams. **a)** ^{230}Th/^{232}Th vs. ^{238}U/^{232}Th and **b)** ^{234}U/^{238}U vs. ^{238}U/^{232}Th) for coeval samples of stratigraphic Layer J (Sample UTh-1) dated at 31.4 ± 14.8 ka. Data points are plotted with 1σ error ellipses (green). The black line represents the best fit regression and the grey-shaded area the 1σ confidence interval.

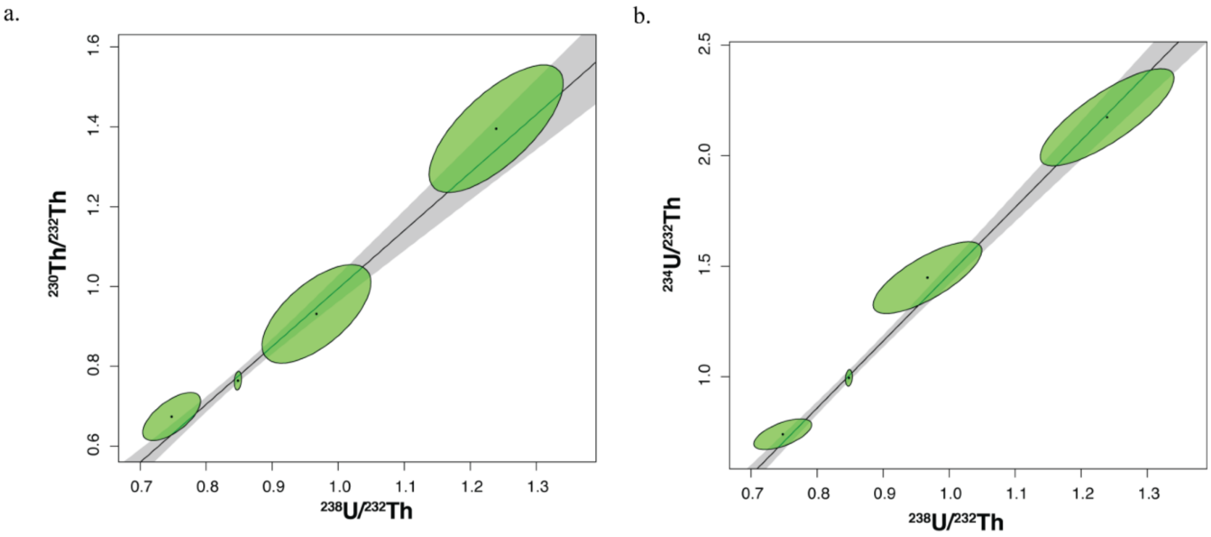

Figure S9. Rosholt type-II isochron diagrams. **a)** $^{230}Th/^{232}Th$ vs. $^{238}U/^{232}Th$ and **b)** $^{234}U/^{232}Th$ vs. $^{238}U/^{232}Th$) for coeval samples of stratigraphic Layer J (Sample E1_1) dated at 71.0 ± 9.0 ka. Data points are plotted with 1σ error ellipses (green). The black line represents the best fit regression and the grey-shaded area the 1σ confidence interval.

Table S9. U-series measurements. U concentration, activity ratios and isochron ages for samples E1_1 and UTh-1 are presented.

Sample	U [ppb]	±	$^{232}Th/^{238}U$	±	$^{234}U/^{238}U$	±	$^{230}Th/^{238}U$	±	Isochron age [ka]*	±
E1_1 a **	35	1	0.807	0.108	1.755	0.170	1.126	0.151	71.0	9.0
E1_1 b **	79	2	1.034	0.143	1.498	0.181	0.963	0.161		
E1_1 c **	118	4	1.167	0.038	0.847	0.041	0.719	0.038		
E1_1 d **	162	4	1.179	0.011	1.173	0.075	0.901	0.043		
E1_1 e **	406	6	1.337	0.125	0.989	0.120	0.901	0.102		
UTh-1 HoAx	175.7	0.3	2.814	0.003	1.120	0.024	1.538	0.002	31.4	14.8
UTh-1 1MHCl	244.2	0.4	2.895	0.004	1.051	0.048	1.547	0.004		
UTh-1 _6MHCl	281.5	0.4	3.193	0.005	1.027	0.089	1.715	0.005		
UTh-1 $15MHNO_3$	242.4	0.4	3.562	0.004	1.042	0.058	1.837	0.003		

All uncertainties are reported as 2**σ**
* Rosholt type II calculated using Isoplot online (http://isoplotr.es.ucl.ac.uk/)
** Measurements done by alpha-spectrometry

Interestingly, only the samples leached with weak acetic acid gave a sufficient spread of the data. Ignition of the carbonate matrix might have altered the clay particles leading to the release of associated detrital Th during the subsequent acid leaching. Similarly, because of the fine-grained texture of the concretion, leaching with 0.1 M HCl might already extract detrital Th and hence lead to a ‘clustering’ of the data. Another important point is that the previous alpha-counting technique used much larger samples (30–50 g), whilst mass spectrometric techniques require less than one gram. The smaller the sample, the more difficult it is to fractionate the cement rather than the detritus. The risk is that all cement – that is very sparse in cave sediment – becomes completely digested in the first fraction, causing the fractionation to be done on the detritus rather than on various phases of cement.

Phylogenetic dating

DNA extraction and library preparation of Vulpes lagopus, Dicrostonyx torquatus and Ursus sp.

One *Vulpes lagopus* (Arctic fox) astragalus, thirteen *Dicrostonyx torquatus* (collared lemming) specimens and one *Ursus* sp. (bear) phalanx and were selected for shotgun sequencing, phylogeographic analyses and phylogenetic dating (Table S10). The bear and collared lemming samples were processed in the ancient DNA laboratory of the University of Oslo, following the same methodology as described for the BBM samples above, with the addition of a bleaching step prior to the pre-digestion (54). Single-indexed sequencing libraries were built using the single-stranded Santa Cruz Reaction (SCR) library protocol with tier 4 adapter dilutions (55). The quality and concentration of each library was assessed with a High Sensitivity NGS Fragment Analysis Kit on the Fragment Analyzer (Agilent), and libraries were sequenced on the Illumina NovaSeq 6000 S4 (2x150 bp PE) at the Norwegian Sequencing Centre (NSC) in Oslo. The Arctic fox was processed in the ancient DNA laboratory of the Centre for Palaeogenetics, University of Stockholm, following protocols described in van der Valk et al. (56), including double stranded library preparation with USER treatment to remove DNA damage, and sequenced on an Illumina NovaSeq X using a 2x150bp setup at the National Genomics Infrastructure (NGI) in Stockholm.

Table S10. Sample information for the *Ursus* sp.*, D. torquatus* and *V. lagopus* specimens for which shotgun sequence data was obtained. Sample number, element, layer, location, bone powder included in the DNA extraction, endogenous content and mitogenome depth of coverage is presented.

Sample No.	Species ID	Bone and side	Layer	Total bone powder (mg)	Location	Endogenous DNA content	Mitogenome depth of coverage
NOC013	*Ursus* sp.	Phalanx	K1	110	98X/51Y NE	0.3%	12.72X*
BDO001	*Dicrostonyx torquatus*	Tibia	U2	37	99X/51Y SE	2.86%	1.1X
BDO002	*Dicrostonyx torquatus*	Tibia	L1	29	99X/50Y SE	14.74%	20.0X**
BDO003	*Dicrostonyx torquatus*	Molar fragment	U2	<10mg	99X/51Y SW	0.07%	0.1X
BDO005	*Dicrostonyx torquatus*	Mandibular M2, right	L1	<10mg	98X/54Y NW	0.02%	0.0X
BDO006	*Dicrostonyx torquatus*	Mandibular M2, right	K1	<10mg	98X/50Y NE	0.07%	0.0X
BDO007	*Dicrostonyx torquatus*	Mandibular M2, right	K1	<10mg	98X51Y NW	0.03%	0.0X
BDO008	*Dicrostonyx torquatus*	Mandibular M2, right	K1	<10mg	98X/51Y NW	0.02%	0.0X
BDO009	*Dicrostonyx torquatus*	Mandibular M2, right	L2	<10mg	99X51Y SE	0.15%	0.0X
BDO010	*Dicrostonyx torquatus*	M2/M3, right	L2	<10mg	99X51Y SE	0.03%	0.0X
BDO011	*Dicrostonyx torquatus*	M1/M2/M3, right	Z1	<10mg	99X/52Y NE	0.03%	0.0X
BDO012	*Dicrostonyx torquatus*	Mandibular molar, left	L2	<10mg	98X/50Y NE	0.02%	0.0X
BDO013	*Dicrostonyx torquatus*	Mandibular molar	K/L	<10mg	99X/52Y NE	0.02%	0.0X
BDO014	*Dicrostonyx torquatus*	Mandibular molar, left	Y1	<10mg	99X/53Y NE	0.03%	0.0X
CAC001	*Vulpes lagopus*	Astragalus	K2	28	98X/51Y NE	1.2%	7X

*Mitogenome coverage mapping to the *U. maritimus* mitogenome. **Mitogenome coverage after iterative assembly with MIA.

Vulpes lagopus phylogenetic analyses and dating

Raw sequencing reads were aligned to the *V. lagopus* mitogenome (Genbank ID: NC_026529.1) using GenErode v0.5.1 as specified above (57). The historical track was used for the ancient sample (CAC001), and the modern track for modern samples (retrieved from Genbank SRR5328115). We retrieved BAM files from Larsson et al. [(58); European Nucleotide Archive ID: PRJEB34472] and called consensus sequences with majority rule using ANGSD v0.933 [(59); -minQ 30, -minmapQ 30, minimum coverage of 3X per site, ambiguous sites = N]. Following alignment in Muscle v.3.8.31 (60), we performed Bayesian reconstruction of the phylogenetic tree with BEAST v.1.10.4 (61) using the HKY+G substitution model (62, 63) inferred by jModelTest v.2.1.10 (64) with tip ages calibrated for Late Pleistocene samples. We assumed a strict molecular clock with the coalescent constant size tree model. Next, we set normal priors for four samples with known dates (PAF05: mean = 22 177.5 years ago, SD = 290.5, PAF06, PAF07: mean = 30,000 years ago, SD = 10,000; PL07: mean = 12,500, SD = 500) and set uniform priors for the mutation rate and non-dated samples (μ: lower = 6.1×10^{-8}, upper = 1.0×10^{-7}; CAC001: lower = 0, upper = 100,000; PL15: lower = 0, upper = 13,000). We ran two independent Markov chain Monte Carlo (MCMC) chains of 100 million generations each, sampling every 1,000. The resulting log files and trees files were combined using LogCombiner 1.10.4, with 10% burnin removed. Convergence was verified in Tracer (effective sample sizes ≥200), and a Maximum Clade Credibility tree was generated using TreeAnnotator v1.10.4 with 10% burnin removed and visualized in FigTree v1.4.4 (http://tree.bio.ed.ac.uk/software/figtree/). We recovered 1.2% endogenous DNA from the *V. lagopus* astragalus and a complete mitogenome with an average depth of coverage of 7X.

The Bayesian phylogeny revealed two main clades consistent with Larsson et al. [(58); Fig. 3]. The earliest maternal split was ~89 ka (95% HPD: 101–77 ka) (Fig. S10; Table S11). The last maternal common ancestor between the Arne Qvamgrotta sample (CAC001) and one Late Pleistocene Russian sample (PAF07; ~30 ka) was dated at ~67 ka (95% HPD: 80–58 ka BP), together forming the basal clade of the phylogeny. The median age of the Arne Qvamgrotta Arctic fox specimen CAC001 was inferred at ~55 ka (95% HPD 66.7–50 ka).

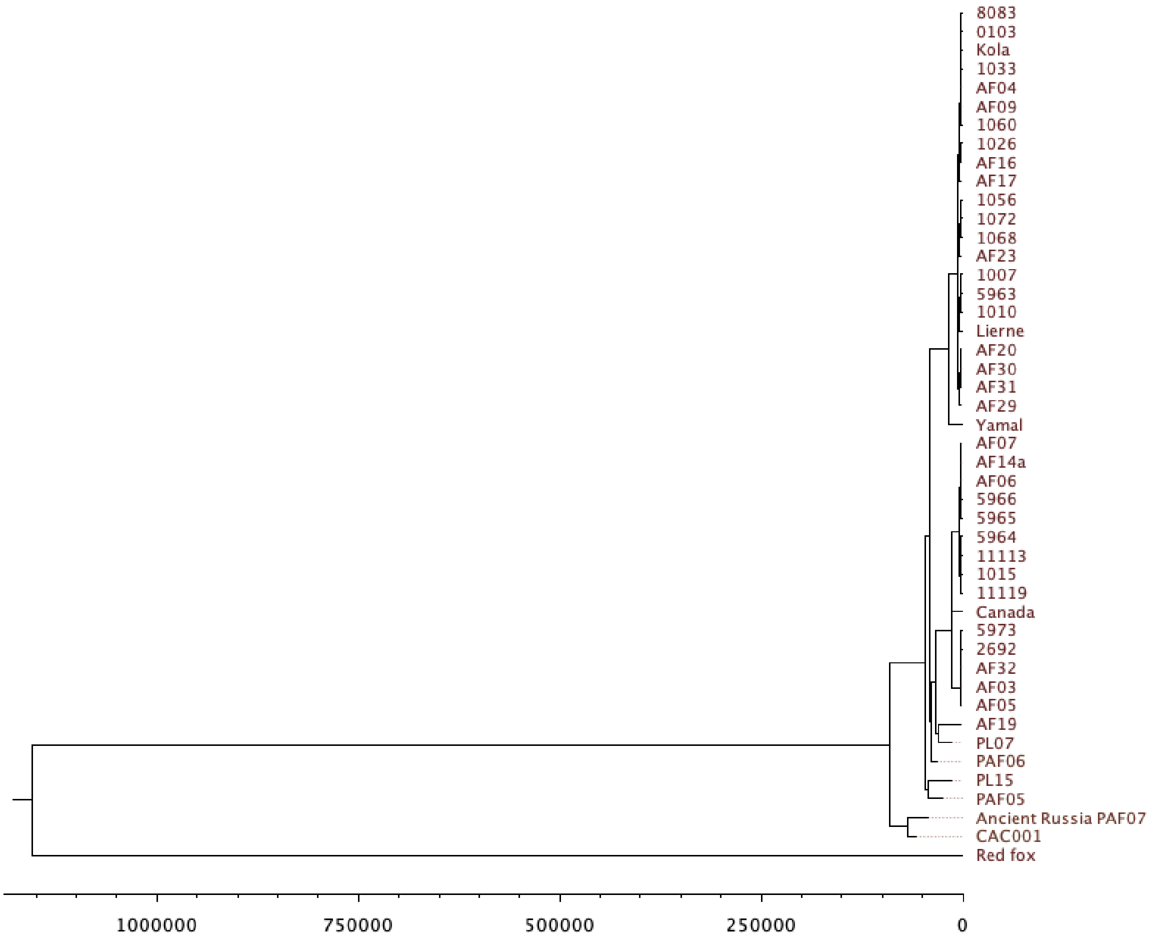

Figure S10. Mitochondrial phylogeny of *Vulpes lagopus* mitochondrial genomes obtained using BEAST v1.10.4, with *Vulpes vulpes* as the outgroup, including one ancient *V. lagopus* (Russia) specimen, and the dataset from Larsson et al. (58). The *V. lagopus* specimen of Arne Qvamgrotta (CAC001) clusters with the Late Pleistocene Russian sample, together forming the basal clade of the phylogeny.

Table S11. Divergence time and tip dates. Sample information, divergence time and tip dates of the Arne Qvamgrotta *Vulpes lagopus*, *Dicrostonyx torquatus* and *Ursus maritimus* specimens and the samples they group with in their respective phylogenies.

Sample ID	Species	Median age	95% HPD lower	95% HPD upper	Median node age	95% HPD lower	95% HPD upper
CAC001	*Vulpes lagopus*	55,289	50,000	66,655	67,007	57,628	79,557
PAF07	*Vulpes lagopus*	41,911	34,748	45,000			
BDO002	*Dicrostonyx torquatus*	72,481	63,457	81,513	81,935	73,398	90,721
E039	*Dicrostonyx torquatus*	49,553	43,011	56,200			
NOC013	*Ursus maritimus*	88,065	72,579	102,316	99,918	92,226	110,339
Bruno-SC16.JK007	*Ursus maritimus*	90,000					

Dicrostonyx torquatus mitogenome reconstruction, phylogenetic analyses and dating

GeneErode v0.5.1 (57) was used to align all raw sequencing data to a concatenated nuclear-mitogenome reference of *D. torquatus* (nuclear genome Genbank ID: GCA_028555095.1; mitogenome Genbank ID: NC_034646.1). The historical track of GenErode was used to allow for specific ancient DNA mapping parameters (aln algorithm, -l 16500, -n 0.01, -o 2). Endogenous DNA was calculated for all samples using a custom script for collating statistics from GenErode (Table S10). One sample, BDO002, with an endogenous content of 14.7%, had sufficient coverage of the mitogenome (7.4X) to proceed with further analyses, although the breadth of coverage was initially low (77.4%). Damage across the reads was visualized using MapDamage v2.0.9 (Fig. S11). In order to increase coverage across the mitogenome, an iterative mapping with MIA (mitogenome iterative assembler) was conducted (65). Three different mitogenomes were used as seeds across independent runs: *D. torquatus* (NC_034646.1), *D. groenlandicus* (NC_034313.1), and *D. hudsonius* (NC_034307.1). The following parameters were specified in MIA: -c -C -U -s ${ANCIENT_DNA_MATRIX} -i -F -k 14 -m (https://github.com/mpieva/mapping-iterative-assembler). Consensus mitogenomes were produced after convergence (4-7 iterations depending on the reference seed) by MIA, and consensus fastas were generated from the final maln file for each run using a custom perl script with a depth of coverage of 5X and a majority rule of 0.8. All consensus sequences were visually inspected in Geneious Prime to confirm that they were identical despite the reference that was used as the initial seed, and we proceeded with the consensus sequence generated using the *D. torquatus* reference. As a check, raw sequencing data was realigned to the MIA consensus FASTA sequence with GenErode as above, and visually checked using Geneious Prime. The median depth of coverage increased to 20X and breadth of coverage increased to 99.9%. A consensus FASTA sequence was generated using ANGSD v0.940 using the majority rule consensus, with the following parameters: -minQ 30, -minmapQ 30, minimum coverage of 5X per site, ambiguous sites = N.

To analyze the mitogenome phylogeny and estimate the age of *D. torquatus* in Arne Qvamgrotta the consensus FASTA sequence for BDO002 was analyzed with a dataset of modern and ancient collared lemmings from Lord et al. (66). Muscle v.3.8.31 (60), was used to align the whole dataset with default parameters, and we used jmodeltest v.2.1.10 (64) to determine the nucleotide substitution model as GTR+I+G based on the Bayesian Information Criteria. BEAUTI v1.10.4 (61) was used to construct the XML file for BEAST v1.10.4 (61) as per Lord et al. (66), with tip date prior information as specified in Additional File Table S5 in Lord et al. (66). A strict molecular clock was used and a constant coalescent tree model. BDO002 was given a uniform prior between 0 and 200,000. Two independent runs of BEAST with 100 million MCMC chains were performed. The log and tree files from each run were combined using LogCombiner v. 1.10.4 with 10% burnin removed. Twenty percent burnin was discarded and the maximum clade credibility tree was constructed using TreeAnnotator. The tree was visualized using FigTree v4.4 (http://tree.bio.ed.ac.uk/software/figtree/) and TreeViewer. Clades 1, 3, 4, and 5 of *D. torquatus* and *D. groenlandicus* were collapsed, and E313 was removed for visual purposes (Fig. 3). The median tip date and 95% highest posterior density interval were calculated for BDO002 and the node for Clade 2 using FigTree (Table S11).

The Bayesian phylogenetic tree shows five distinct clades (Fig. 3; Fig S12), which is consistent with previous *D. torquatus* mitochondrial studies (66–68). Sample BDO002 falls in Clade 2 (Fig. 3; Fig S12), along with a Late Pleistocene sample from Belgium (E039). Clade 2 has a node divergence of ~81 ka BP (95% Higher Posterior Density [HPD]: 90–73 ka; Table S11). Using the molecular clock, we estimated the date of BDO002 to be ~72 ka (95% HPD: 81.5–63.5 ka; Table S11).

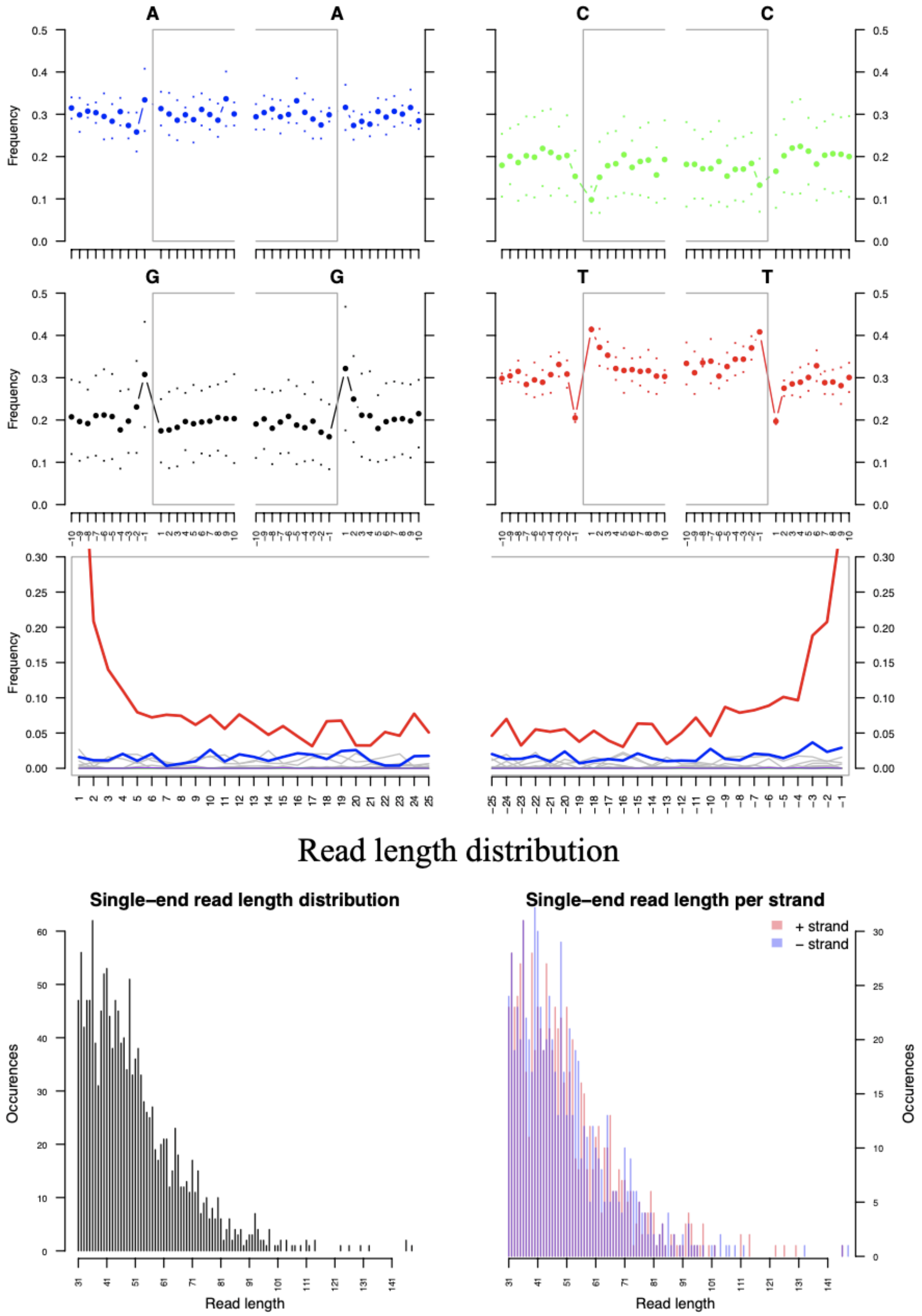

Figure S11. Damage plots and read length distribution for BDO002 mapped to the *D. torquatus* mitochondrial reference genome (NC_034646.1). Plots were generated using mapDamage.

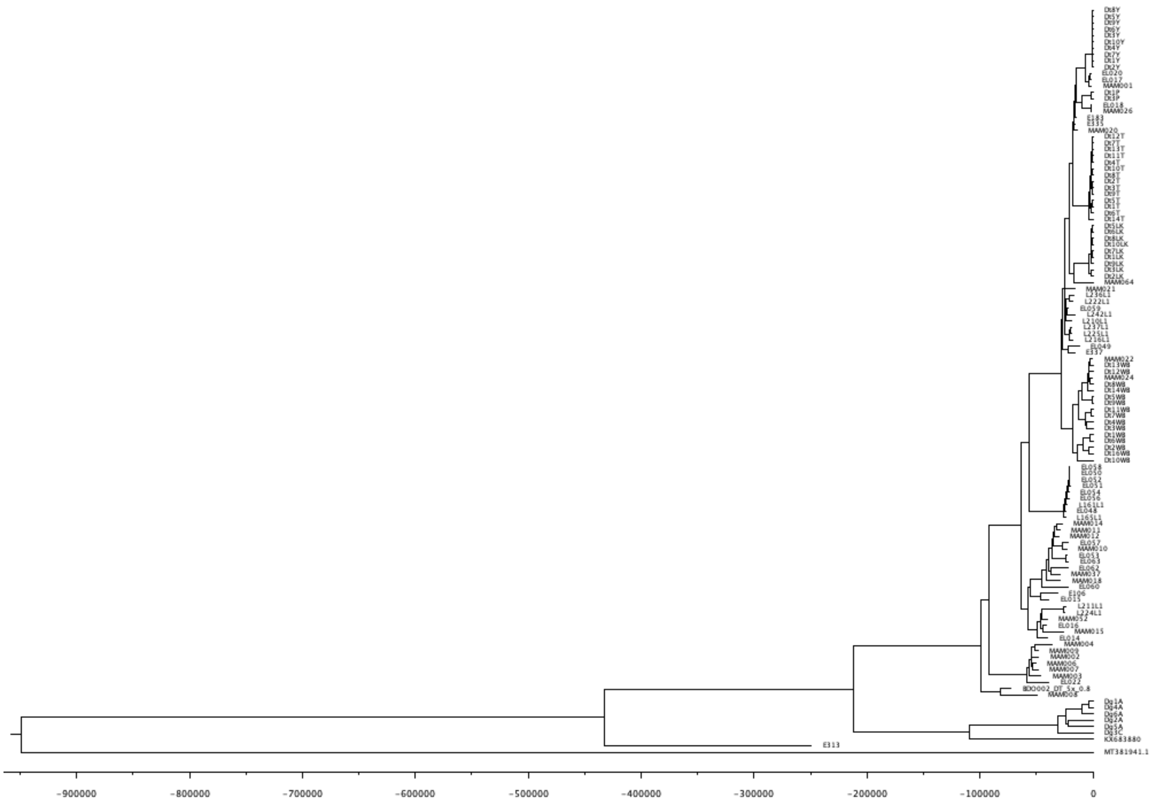

Figure S12. Mitochondrial phylogeny of *D. torquatus* mitochondrial genomes obtained using BEAST v1.10.4, with *Phenacomys intermedius* as the outgroup, and including the dataset from Lord et al. (66). The *D. torquatus* specimen from Arne Qvamgrotta clusters with the sample from Belgium within the extinct clade 2 (see also Fig. 3c). The tree was visualized using FigTree v4.4 (http://tree.bio.ed.ac.uk/software/figtree/) and TreeViewer.

Ursus sp. phylogenetic analyses and dating

In total, 394,418,850 DNA fragments were generated of NOC013 (Table S10). Reads were processed in PALEOMIX v1.3.8 (69). Adapters were removed from reverse and forward reads using AdapterRemoval v2.3.1 [(70); --mm3 --minlength25 --collapse yes --trimns yes --trimqualities yes] and reads were mapped using BWA backtrack v0.7.17 (71) to the reference *U. maritimus* nuclear genome (NCBI accession number: GCF_017311325) and the reference mitochondrial genome [NCBI accession number: NC_003428; (72, 73)]. The DNA damage patterns and read length distribution generated by mapDamage show typical ancient DNA fragmentation and damage patterns (Fig. S13) We recovered 0.3% endogenous DNA, with a coverage of 0.021X and of 12.78X for the nuclear and the mitochondrial genome, respectively, when mapping to *U. maritimus*. Reads were also aligned against the *U. arctos* nuclear (ASM358476v1 from DNAzoo) and mitochondrial genome (NCBI accession number NC_003427.1) resulting in a coverage of 0.021X and 11.78X of the nuclear and mitochondrial genome, respectively.

A phylogeny of the mitochondrial genome with our sequences mapped to *U. maritimus* was built with 40 bear mitogenomes from Wang et al. (73), including one 100,000 year old specimen from Alaska and one 100,000–130,000 specimen from Svalbard (72, 73). Raw data from the Alaskan specimen was downloaded from NCBI and mapped using the above-mentioned procedure. We used BCFtools v.1.15.1 (74) for consensus sequences and set ploidy to 1 to take into account the haploid mitogenome data. Variants with genotype quality <30 and depth <3 as well as indels were removed using BCFtools v.1.15.1 and VCFtools v.0.1.16 (75). All mitogenomes were aligned using Muscle in MEGA (v.11.0.13) (60) and a dated mitogenome phylogeny was generated with BEAST

1.10.4. A strict clock was used, and the nucleotide model was specified as HKY with invariant sites (I) and Gamma distribution (G4) as determined by jModelTest v.2.1.10 (64). We used a coalescent constant size tree model, with a normal prior given to the clock rate as per (76) (mean=2.48E-8, initial=2.48E-8, sd=0.01), and the mutation rate of 2.48×10^{-8} mutations per site per year (77). The four previously published ancient bear samples had radiocarbon or estimated ages included as tip dates (Table S12). The specimen from Arne Qvamgrotta, NOC013, was given a uniform prior of 0-200000. Two independent analyses were run for 100 million generations each, sampling every 1000 states. Convergence was assessed using Tracer, with estimated sampling sizes all >200. Both the log files and trees files were combined using LogCombiner 1.10.4, with 10% burnin removed. A maximum clade credibility tree was generated using TreeAnnotator 1.10.4, with 10% burnin removed. The tree was visualized using FigTree v4.4.

NOC013 is sister to the ancient Alaskan polar bear, supporting its identification as *U. maritimus,* with a median age of ~88 ka (95% HPD: 102.2−72.5 ka; Fig. 3, Fig. S14, Table; S11; S20). All three ancient polar bears fall outside the modern clades analyzed.

Table S12: Genbank accession and ages of two ancient *U. spelaeus* and two ancient *U. maritimus* samples included in the mitogenome phylogeny presented in Fig. 3 and Fig. S14.

Sample	Reported age	Median	Error (years)	Study
EU327344.1	31,870 +300/−270	31,870	300	(78)
NC_011112	44,160 +1400/-1190	44,160	1400	(79)
GU573488.1	130,000−110,000	120,000	10,000	(72, 80)
SRR14329358 (Bruno-SC16.JK007)	110,000−70,000	90,000	20,000	(73)

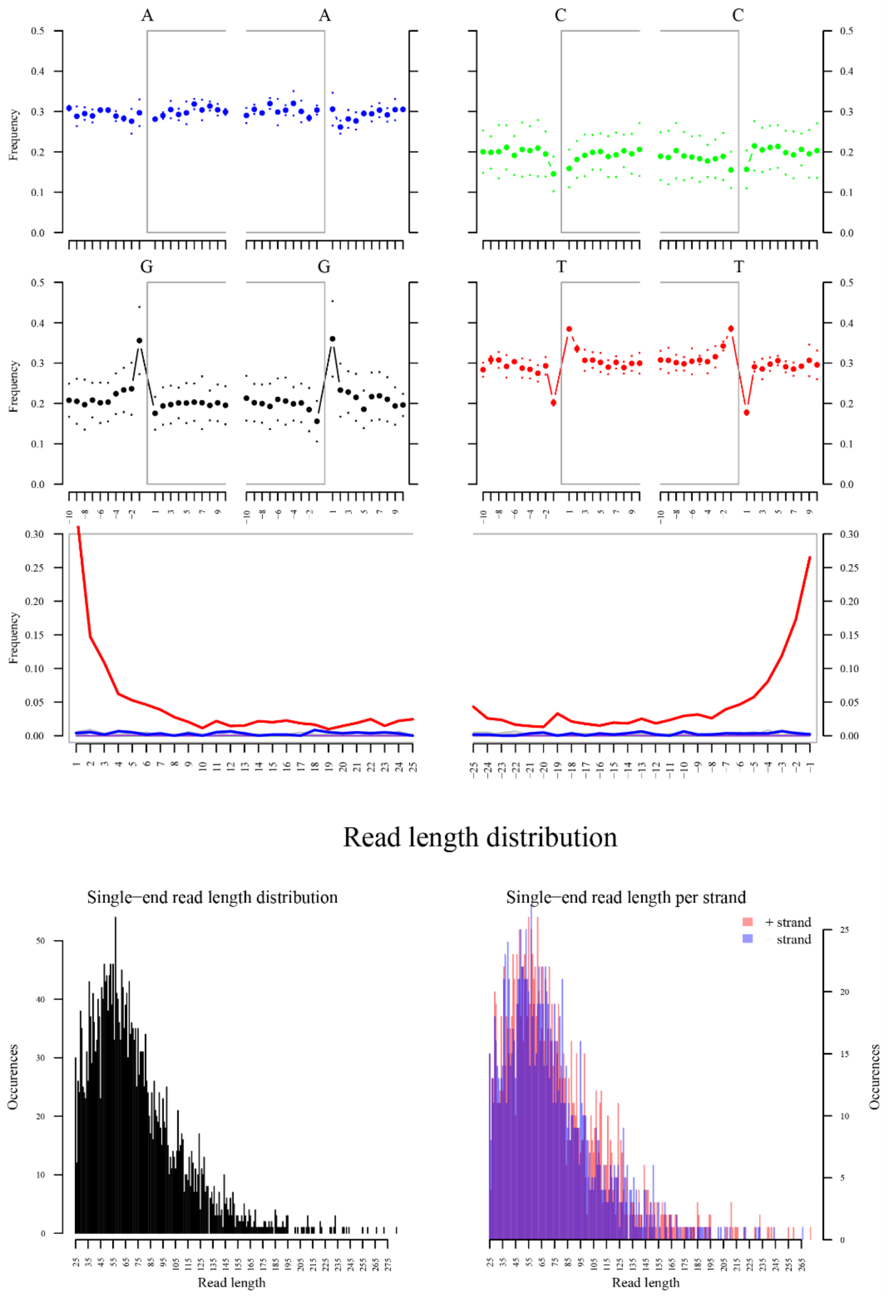

Figure S13. Damage plots and read length distribution for NOC013 mapped to the *U. maritimus* mitochondrial reference genome (NC_003428). Plots were generated using mapDamage.

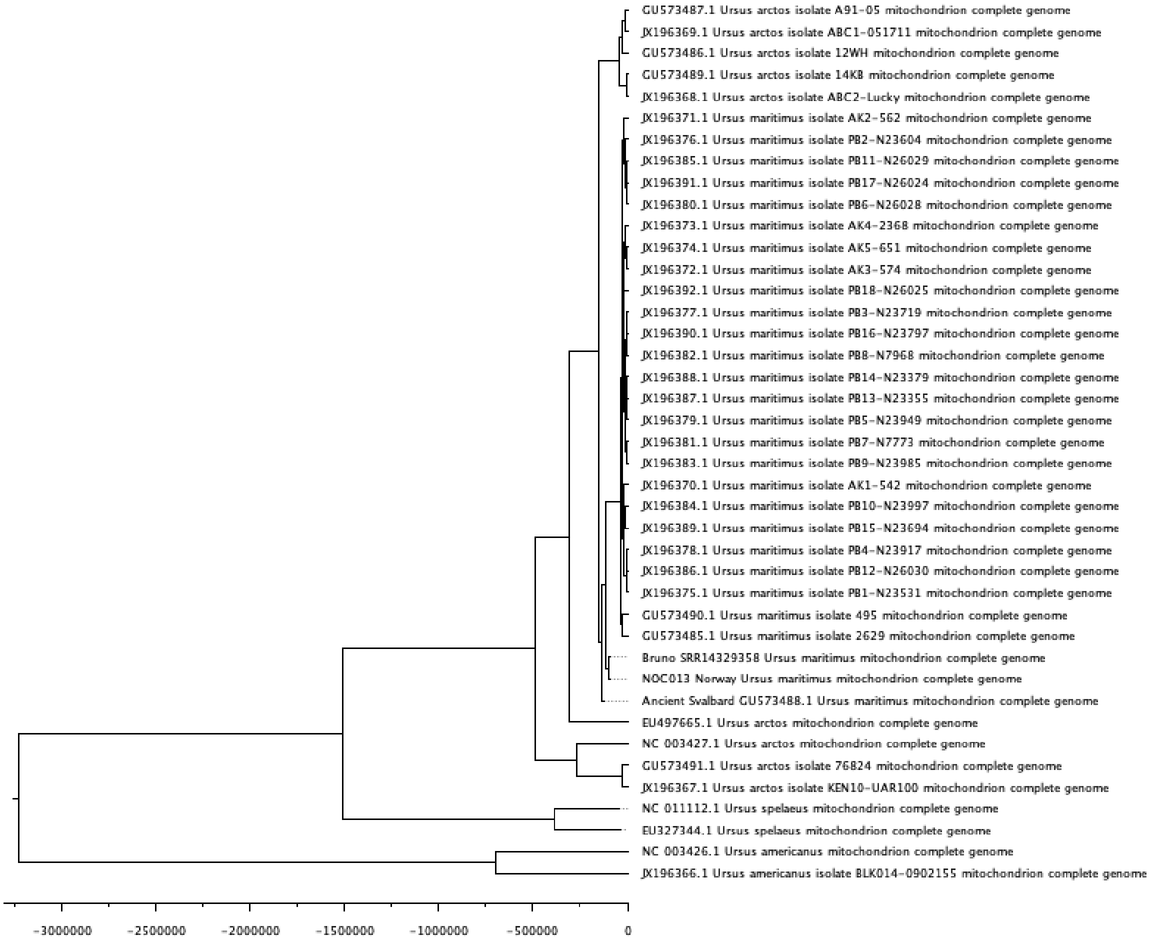

Figure S14. Mitochondrial phylogeny of *Ursus* sp. mitochondrial genomes obtained using BEAST v1.10.4, including two ancient *U. maritimus* (Svalbard, Alaska) specimens, 36 modern specimens of *U. maritimus*, *U. arctos* and *U. americanus*) and two ancient *Ursus spelaeus* specimens. The *Ursus* sp. specimen of Arne Qvamgrotta clusters with the ancient Alaskan *U. maritimus*.

Cosmogenic nuclide burial dating

Description of sample material

Coarse grained sediments in the Kjøpsvik caves can in theory be suitable for simple burial dating [e.g., (81)] with $^{26}Al/^{10}Be$ as they contain quartz and quartz-bearing lithologies sufficiently deep to ignore post-burial nuclide production. For burial dating to work, sediments must have been exposed at the surface and then transported into the cave. As the most likely transport mechanism for the Kjøpsvik caves is subglacial, the non-carbonate minerogenic cave fill can potentially have been derived via glacial erosion and transport of material with short to non-existing surface exposure, and even sourced from bedrock well below surfaces exposed to cosmic radiation. Several studies have successfully applied cosmogenic nuclide burial dating on caves in formerly glaciated settings [e.g., (82–84)], but the technique has not yet been applied to sites previously covered by the Scandinavian Ice Sheet. In theory, this technique could provide age estimates for sediments' residence times in the cave, and potentially the age of different stratigraphic units if they reflect multiple episodes of sediment injection.

Sample preparation

Four samples from the stratigraphic section in the AQ passage were selected for piloting the application of $^{26}Al/^{10}Be$ burial dating (Table S13). The chosen samples were from the top surface (KJØ 2108-B, gneiss cobble), U1–U5 (KJØ 2112-A, gneiss cobble; KJØ 2112-B, biotite gneiss cobble) and U5-U6 (KJØ 2110-A, garnet gneiss cobble). Preparation of targets for AMS analysis was done at the University of Bergen (UiB) Cosmogenic Nuclide Preparation Facility. The mineral separation protocol largely follows Kohl and Nishiizumi (85). After sample disintegration, the 250–500 mm grain size fraction went through the necessary steps to isolate and enrich quartz. The quartz fraction (sub-sample sizes of max. 40 g) was purified by four repeated 3-day etches in 2% HF and 2% HNO_3. Quartz purity was assessed by Inductively Coupled Plasma Optical Emission Spectrometry (ICP-OES). Between 79 and 100 g of the purified quartz (Table S13) was spiked with 255 µg of the in-house Be carrier and dissolved. The in-house Be carrier (PHE1601) has a seven-year mean $^{10}Be/^{9}Be$ value for chemical process blanks of (9.83 ± 1.39) x 10^{-16} (n = 49, analyzed at Aarhus AMS Centre, AARAMS). The performance of the UiB preparation laboratory has since 2018 been monitored by preparation and measurement (AARAMS and COLOGNE) of subsamples of the reference material CoQtz-N. This yields a ^{10}Be nuclide concentration of (2.606 ± 0.018) x 10^{6} at g^{-1} (n = 14), which lies within the (2.53 ± 0.09) x 10^{6} at g^{-1} value reported by Binnie et al. (86). No addition of Al carrier to the four samples was necessary. After quartz digestion, Al splits were taken and the concentrations of Be and Al were determined with ICP-OES (Table S13). [Al] ranged from 19.9 ± 0.2 to 58.3 ± 0.7 µg g^{-1} and Be ranged from 2.53 ± 0.5 to 3.2 ± 0.6 µg g^{-1}. Be and Al was extracted following modified procedures of Kohl and Nishiizumi (85) and Child et al. (87).

AMS analysis

From the processed samples and one process blank, $^{10}Be/^{9}Be$ ratios of BeO targets and $^{26}Al/^{27}Al$ ratios of Al_2O_3 targets, were analyzed at Aarhus AMS Centre, AARAMS, using a multi-element AMS system (88). Be results are normalized to the ICN standard 01-5-4 with a $^{10}Be/^{9}Be$ ratio of 2.851 x 10^{-12} (89), and sample errors include 1.1% uncertainty of the ICN standard. All BeO targets included in this specific AMS run showed unusually high ^{10}B (average of 11.9 sec^{-1} $microA^{-1}$, n = 53) compared to an earlier run in 2023 (<3 ^{10}B, n = 39) for targets prepared in Bergen. The blank-corrected $^{10}Be/^{9}Be$ ratios (Table S13) for the four samples range from 0.5 to 1.1 x 10^{-14} with 1 SE between 17 to 44%. Al results are normalized to ICN-01-5-2 with a $^{26}Al/^{27}Al$ ratio of 1.82 x 10^{-12} [1 s = 1.1%, (89)]. The blank-corrected $^{26}Al/^{27}Al$ ratios (Table S13) for the four samples range from 0.2 to 4.8 x 10^{-14} with 1 SE between 20 and 78%.

Results

The ^{10}Be concentrations (Table S13) calculated from the blank-corrected $^{10}Be/^{9}Be$ ratios range from 0.9 to 2.4 x 10^{3} at g^{-1}, and the corresponding ^{26}Al concentrations range from 2.2 to 21.1 x 10^{3} at g^{-1}. The resulting $^{26}Al/^{10}Be$ ratios (Table S13) are, in stratigraphic order from the top, 4.8 ± 1.5 (KJØ 2108-B), 8.1 ± 3.0 (KJØ 2112-A), 2.5 ± 2.2 (KJØ 2112-B), and 9.0 ± 2.3 (KJØ 2110-A).

Table S13. Results of the paired cosmogenic ^{26}Al and ^{10}Be analyses. Element concentrations, isotope ratios, nuclide concentrations, and nuclide ratios for the four samples analyzed for burial dating using paired cosmogenic ^{26}Al and ^{10}Be.

Sample ID	Layer	Quartz (g)	ICP-OES		AMS		[^{26}Al] (x10^3 at g^{-1})	[^{10}Be] (x10^3 at g^{-1})	$^{26}Al/^{10}Be$
			[Al] (µg g^{-1})	[Be] (µg g^{-1})	$^{26}Al/^{27}Al$ (x 10^{-14})	$^{10}Be/^{9}Be$ (x 10^{-14})			
2108-B	A	99.78560	20.33 ± 0.12	2.53 ± 0.01	1.94 ± 0.45	1.08 ± 0.24	8.80 ± 2.04	1.84 ± 0.41	4.8 ± 1.5
2112-A	U1	96.57720	58.30 ± 0.41	2.63 ± 0.02	2.10 ± 0.52	1.07 ± 0.29	15.24 ± 3.77	1.89 ± 0.52	8.1 ± 3.0
2112-B	U1	100.19920	19.85 ± 0.04	2.56 ± 0.01	0.17 ± 0.13	0.53 ± 0.23	2.21 ± 1.69	0.90 ± 0.40	2.5 ± 2.2
2110-A	U1	79.02610	32.51 ± 0.10	3.20 ± 0.02	4.76 ± 0.93	1.09 ± 0.19	21.09 ± 4.12	2.36 ± 0.40	9.0 ± 2.3

Calculations of simple burial histories have not been attempted due to 1) the large uncertainties of the AMS ratios, and 2) the fact that two of the samples give $^{26}Al/^{10}Be$ ratios that plot in what is considered the "forbidden" zone. If the <5 $^{26}Al/^{10}Be$ ratios would be considered reliable, the sediments have burial ages exceeding 500 ka, which would not be relevant for the dating of the analyzed bone-bearing layers.

Text S5. Comparative osteology

The osteological analysis was performed through comparative osteology using the reference collection at the Department for Natural History, University Museum of Bergen. In addition to the modern reference collection, several identification manuals, specifically relating to small mammals, were utilized (90, 91). All bone material collected from the excavated sediments was analyzed.

Species abundance was quantified based on the Number of Identified Specimens (NISP; Table S14). Wherever possible fragments which refit (i.e., belong to a single specimen) were counted as one to prevent over-representation of species. Due to the lack of identifiable material available and the rarity of a site this age, all bone elements which could be identified were included within the NISP counts (i.e., including vertebrae and phalanges; Table S15). The Minimum Number of Individuals (MNI; Table S14) was calculated for each species and genus. This quantification method takes into account both side and bone element to give the lowest possible number of individuals present at a site. Taxa counts were calculated so as to avoid over-representation of the overall number of taxa. To do this we disregarded identifications above family level, and we only counted family level identifications if we did not have any genus or species level identifications within that specific family. All genus and species level identifications were counted.

Taphonomic markers were recorded where present and followed guidance outlined in Fernández-Jalvo and Andrews (92). Further digital recording of the few larger, complete bones was performed using a 3D scanner (Creaform Black Elite Handy Scan) especially when these were sampled for DNA analysis. To minimize contamination whilst handling the bones, face masks and gloves were used at all times.

Table S14. Number of Identified Specimens (NISP) and Minimum Number of Individuals (MNI) from Arne Qvamgrotta. Osteological identification and NISP are given by layer (Fig. 1; Fig. 2; numbers indicate mechanical layer within stratigraphic layer). NISP was calculated taking into account all elements which were identified beyond class. Due to the fragmentary nature and small amount of identifiable material vertebrae and phalanges were included and are likely to have little effect on the final figures. Ribs were not identified. MNI figures do not take into consideration deposition in different layers, and therefore represent the very minimum number of individuals. MNI is only calculated for taxonomic level genus and higher.

Species ID	Y1	Z1	K1	K2	K/L	L1	L2	L/U	U1/2	U3/4	U5/6	U7/8	U9/10	Q1	NISP	MNI
**Aves**																
Anatidae	-	1	2	-	-	-	-	1	-	-	-	-	-	-	4	NA
Somateria mollissima	-	-	-	-	-	1	-	-	-	-	-	-	-	-	1	1
Tetraoninae	-	-	1	-	-	-	-	-	-	-	-	-	-	-	1	NA
Lagopus sp.	-	-	1	-	-	-	-	-	-	-	-	-	-	-	1	1
Alcidae	-	-	-	1	-	2	-	1	-	-	-	-	-	-	4	NA
Uria sp.	-	1	-	-	-	-	1	-	-	-	-	-	-	-	2	1
Passeriformes	-	1	1	-	-	1	-	-	-	-	-	-	-	-	3	NA
**Mammalia**																
Lepus sp.	-	1	-	-	-	1	-	-	-	-	-	-	-	-	2	1
Rodentia	12	16	69	10	8	31	7	21	-	-	-	-	-	1	175	NA
Cricetidae	-	-	-	-	-	5	-	1	-	-	-	-	-	-	6	NA
Arvicolinae	-	-	7	-	2	1	8	1	-	-	-	-	-	-	19	NA
Microtus sp.	-	-	2	-	-	-	-	-	-	-	-	-	-	1	3	1
cf. *Alexandromys oeconomus*	-	-	-	1	-	-	-	-	-	-	-	-	-	-	1	1
Alexandromys oeconomus	-	-	1	-	-	-	-	-	-	-	-	-	-	-	1	1
cf. *Dicrostonyx torquatus*	3	3	11	2	1	4	1	-	-	-	-	-	-	-	25	4
Dicrostonyx torquatus	5	5	36	-	2	10	10	2	3	-	-	-	-	-	73	9
Cervidae	-	-	3	-	-	-	-	-	-	-	-	-	-	-	3	NA
cf. *Rangifer tarandus*	-	2	-	-	-	-	-	-	-	-	-	-	-	-	2	1
Rangifer tarandus	-	1	-	-	1	-	-	-	-	-	-	-	-	-	2	1
cf. *Ursus* sp.	-	1	-	-	-	-	-	-	-	-	-	-	-	-	1	1
Ursus sp.	-	-	3	-	-	-	1	-	-	-	-	-	-	-	4	1
Vulpes lagopus	-	-	1	1	1	-	-	-	-	-	-	-	-	-	3	1
Canis lupus	-	-	1	-	1	-	-	-	-	-	-	-	-	-	2	1
Pusa sp.	-	1	-	-	-	-	-	-	1	-	-	-	-	-	2	1
**Pisces**																
Gadidae	-	-	1	-	-	-	1	1	-	-	-	-	-	-	3	NA
Sebastes norvegicus	-	-	-	-	-	1	-	-	-	-	-	-	-	-	1	1
cf. *Salvelinus* sp.	1	1	1	-	-	2	-	-	-	-	-	-	-	-	5	1
Cottidae	-	-	1	-	-	-	-	-	-	-	-	-	-	-	1	NA
Total Identified	21	33	143	15	16	59	29	28	4	0	0	0	0	2	350	29
Unidentified Aves	-	1	15	1	1	6	2	3	-	-	-	-	-	-	29	
Unidentified Mammalia	53	43	242	8	48	109	222	41	3	-	-	-	-	-	769	
Unidentified Pisces	10	6	130	6	33	44	9	31	1	-	-	-	-	-	270	
Unidentified	685	395	1859	109	117	941	250	377	31	6	-	1	1	1	4773	
Total Unidentified	748	445	2246	124	199	1100	483	452	35	6	0	1	1	1	5841	
TOTAL	769	478	2389	139	215	1159	512	480	39	6	0	1	1	3	6191	29

Table S15. Table of elements. Breakdown of taxa identified by skeletal element. This data was used to calculate Minimum Number of Individuals (MNI; Table S14).

	Anatidae	*Somateria mollissima*	Tetraoninae	*Lagopus* sp.	Alcidae	*Uria* sp.	Passeriformes	*Lepus* sp.	Rodentia	Cricetidae	Arvicolinae	*Microtus* sp.	cf. *Alexandromys oeconomus*	*Alexandromys oeconomus*	cf. *Dicrostonyx torquatus*	*Dicrostonyx torquatus*	Cervidae	cf. *Rangifer tarandus*	*Rangifer tarandus*	cf. *Ursus* sp.	*Ursus* sp.	*Vulpes lagopus*	*Canis lupus*	*Pusa* sp.	Gadidae	*Sebastes norvegicus*	cf. *Salvelinus* sp.	Cottidae	Total
Antler	-	-	-	-	-	-	-	-	-	-	-	-	-	-	-	-	1	-	-	-	-	-	-	-	-	-	-	-	1
Cranium/maxilla/ premaxilla	-	-	-	-	-	-	-	-	8	-	4	-	-	-	1	-	-	-	-	-	-	-	-	-	-	-	-	-	13
Mandible	-	1	-	-	-	-	-	-	1	-	1	-	-	-	5	2	-	-	-	-	-	-	-	-	-	-	-	-	10
Mandible/maxilla	-	-	-	-	-	-	-	-	1	-	3	-	-	-	-	-	-	-	-	-	-	-	-	-	-	-	-	-	4
Loose teeth/Dentary	-	-	-	-	-	-	-	1	2	-	6	-	-	-	4	30	2	-	-	-	-	-	-	1	3	1	-	-	50
Loose Incisor	-	-	-	-	-	-	-	1	50	2	-	-	-	-	-	-	-	-	-	-	-	1	-	1	-	-	-	-	55
Mandibular m1	-	-	-	-	-	-	-	-	-	-	-	-	1	1	-	17	-	-	-	-	-	-	-	-	-	-	-	-	19
Mandibular m2	-	-	-	-	-	-	-	-	-	-	-	1	-	-	-	9	-	-	-	-	-	-	-	-	-	-	-	-	10
Mandibular m3	-	-	-	-	-	-	-	-	-	-	-	-	-	-	-	5	-	-	1	-	-	-	-	-	-	-	-	-	6
Maxillary M1	-	-	-	-	-	-	-	-	-	-	-	1	-	-	-	3	-	-	-	-	-	-	-	-	-	-	-	-	4
Maxillary M2	-	-	-	-	-	-	-	-	-	-	-	-	-	-	-	4	-	-	-	-	-	-	-	-	-	-	-	-	4
Maxillary M3	-	-	-	-	-	-	-	-	-	-	-	1	-	-	-	4	-	-	-	-	-	-	-	-	-	-	-	-	5
Quadratum	-	-	-	-	-	1	-	-	-	-	-	-	-	-	-	-	-	-	-	-	-	-	-	-	-	-	-	-	1
Axis	-	-	-	-	-	-	-	-	1	-	-	-	-	-	-	-	-	-	-	-	-	-	-	-	-	-	-	-	1
Vertebra	1	-	1	-	1	-	-	-	11	-	-	-	-	-	-	-	-	-	-	-	1	1	-	-	-	-	5	1	22
Sacrum	-	-	-	-	-	-	-	-	-	-	-	-	-	-	-	-	-	-	-	1	-	-	-	-	-	-	-	-	1
Sternum	1	-	-	-	-	-	-	-	-	-	-	-	-	-	-	-	-	-	-	-	-	-	-	-	-	-	-	-	1
Scapula	1	-	-	-	-	-	1	-	9	-	1	-	-	-	-	-	-	-	-	-	-	-	-	-	-	-	-	-	12
Humerus	-	-	-	-	-	-	-	-	19	1	1	-	-	-	-	-	-	-	-	-	-	-	-	-	-	-	-	-	21
Radius	-	-	-	-	-	-	-	-	5	-	-	-	-	-	-	-	-	-	-	-	-	-	-	-	-	-	-	-	5
Ulna	-	-	-	-	-	-	1	-	6	-	1	-	-	-	-	-	-	-	-	-	-	-	-	-	-	-	-	-	8
Metapodial	-	-	-	-	-	-	-	-	14	-	-	-	-	-	-	-	-	-	-	-	-	-	-	-	-	-	-	-	14
Pelvis	-	-	-	-	-	-	-	-	6	-	-	-	-	-	-	-	-	-	-	-	-	-	-	-	-	-	-	-	6
Femur	-	-	-	-	-	1	-	-	17	-	-	-	-	-	-	-	-	-	-	-	-	-	-	-	-	-	-	-	18
Tibia/Tibiotarsus	-	-	-	-	-	-	1	-	16	2	2	-	-	-	8	-	-	-	-	-	1	-	-	-	-	-	-	-	30
Fibula	-	-	-	-	-	-	-	-	-	-	-	-	-	-	-	-	-	-	-	-	-	-	1	-	-	-	-	-	1
Carpals	1	-	-	1	1	-	-	-	-	-	-	-	-	-	-	-	-	1	1	-	-	-	-	-	-	-	-	-	5
Astragalus	-	-	-	-	-	-	-	-	1	1	-	-	-	-	-	-	-	-	-	-	-	1	-	-	-	-	-	-	3
Calcaneus	-	-	-	-	-	-	-	-	-	-	-	-	-	-	6	-	-	-	-	-	-	-	-	-	-	-	-	-	6
Sesamoid	-	-	-	-	-	-	-	-	-	-	-	-	-	-	-	-	-	-	-	-	-	-	1	-	-	-	-	-	1
Phalanx	-	-	-	-	2	-	-	-	8	-	-	-	-	-	-	-	-	1	-	-	2	-	-	-	-	-	-	-	13
Total	4	1	1	1	4	2	3	2	175	6	19	3	1	1	24	74	3	2	2	1	4	3	2	2	3	1	5	1	350

Text S6. Ancient DNA bulk-bone metabarcoding

Laboratory methods

Bone fragments from quadrants 99X/51Y and 99X/53Y were used for bulk-bone metabarcoding (BBM) analyses. A total of 47 bulk samples of unidentifiable bone fragments were divided into four groups (Mammalia, Pisces, Aves and unidentifiable vertebrates). Following milling of the bones using a bone crusher (93), DNA was extracted from 1–3 subsamples per milled bulk-sample, with each subsample weighing up to ~110 mg and making a total of 78 DNA extracts (Table S16). We counted the total number of distinguishable fragments per sample, but the majority of fragments were under 3 mm and included many fragments that were close to bone powder that could not be counted (e.g., fragments disintegrated to bone powder after having been manipulated). We milled and mixed all powder to maximize the detection potential of all taxa. A selection of 16 bones (12 birds and 4 mammals) excavated in 2022 were also analyzed by metabarcoding without being included in bulk samples (Table S17).

All pre-PCR laboratory work was carried out in the dedicated ancient DNA laboratory at the University of Oslo. For both pre-digestion and DNA extraction we followed the protocols of Lord et al. (66). Samples were pre-digested in 700 ųl extraction buffer (Urea 1M, EDTA 0.5M at pH8) with 15 ųl proteinase K (18 mg/ml) at 55°C for 30 minutes. Pre-digestion was followed by an overnight digestion (on a nutator at 55°C) with fresh extraction buffer and proteinase K. The following day, 500 ųl of supernatant was concentrated to ~100 ųl using an Amicon Ultra-0.5 Centrifugal Filter Unit (30kDa MWCO). The concentrated supernatant was then purified with a MinElute PCR Purification Kit (QIAGEN) and eluted in 100 ųl elution buffer (EB buffer).

For DNA amplification, we used the Mamp007 primers for the amplification of mammal DNA [5'-CGAGAAGACCCTATGGAGCT-3', 5'-CCGAGGTCRCCCCAACC-3'; Giguet-Covex et al., (94)], the Fish16S primers for the amplification of fish DNA [5'-TACCAAAAACATCGCCTCYTG-3', 5'-CATTTAAAAGACAAGTGATTRCG-3'; designed by Laura Epp (8)] and the Aves12S primers for the amplification of bird DNA [5'-GATTAGATACCCCACTATGC-3', 5'-GTTTTAAGCGTTTGTGCTCG-3'; Epp et al., (95)]. Extracts from samples with unidentifiable vertebrates were amplified with all three primer sets. PCR reactions (25 ųl) contained 2 units of AmpliTaq Gold DNA Polymerase (Applied Biosystems), 15 mM (Tris-HCL), 150 mM (KCl), 10X amplification buffer, 0.2 mM of each dNTP, 2.5 mM $MgCl_2$, 8 ųg BSA, 0.2 ųM of each primer and 5 ųl of template DNA. A human blocking primer (MamP007_B_Hum1, 5′-GGAGCTTTAATTTATTAATGCAAACAGTACCC-3′) was added at a concentration 2 ųM to the PCR reaction with Mamp007 in order to reduce the amplification of human DNA (94, 96). Each primer (forward and reverse) was tagged with a unique identifier of 8–9 bp at the 5' end to allow for multiplexing. Each primer pair shared the same tag and each primer-tag combination was used only once. The full list of tags can be found in Taberlet et al. (97). The conditions for PCR cycling were as follows: 10 minutes at 95°C, 40 cycles at 95°C for 30s, annealing temperature for 30s, 1 minute at 72°C, and a final extension of 10 minutes at 72°C. An annealing temperature of 55°C was used for the mammal and bird primers, and 50°C was used for the fish primers. Negative controls were included in all DNA extractions and PCRs and all negative controls were sequenced. Amplification was assessed on agarose gels (1.5%) and pooling of PCR products was done according to the strength of the bands (three categories were recognized: strong, medium and weak amplification). The pooled PCR products were purified with the MinElute PCR Purification Kit (QIAGEN) and final concentrations of the pools were measured using the Qubit 2.0 dsDNA BR Assay Kit (Thermo Fisher). Libraries were built using the TruSeq DNA Nano library preparation kit and sequenced on the Illumina NovaSeq 6000 S4 (150 bp PE) at the Norwegian Sequencing Center.

Table S16. Sample information of the BBM samples. Metabarcoding group (Aves, Mammalia, Pisces and Unidentified vertebrate), layer from which the bones were retrieved (Fig. 1; Fig. 2; numbers indicate mechanical layer within stratigraphic layer), number of subsamples from which DNA was extracted, total bone powder from the sample following milling, total number of countable fragments in the sample, quadrant and year the sample was excavated are presented. The number of countable fragments does not include fragments that were close to bone powder. DNA was extracted from up to three subsamples of ~110 mg where that amount was available, for a total of 78 subsamples analyzed.

BBM sample No.	Group	Layer	No. of samples	Total bone powder (mg)	Total number of countable fragments	Location	Excavation year
BBO-007	Pisces	L1	1	9	6	99X/51Y	2021
BBO-010	Unidentified	L1	1	10	6	99X/51Y	2021
BBO-011	Aves	L2	1	10	1	99X/51Y	2021
BBO-014	Mammalia	L2	3	2810	154	99X/51Y	2021
BBO-015	Pisces	L2	1	203	14	99X/51Y	2021
BBO-016	Unidentified	L2	3	1238	142	99X/51Y	2021
BBO-019	Aves	L/U	1	35	1	99X/51Y	2021
BBO-020	Mammalia	L/U	3	798	30	99X/51Y	2021
BBO-021	Unidentified	L/U	1	95	11	99X/51Y	2021
BBO-022	Mammalia	U1	3	685	2	99X/51Y	2021
BBO-023	Pisces	L/U	1	12	4	99X/51Y	2021
BBO-024	Mammalia	K1	3	1220	168	99X/51Y	2021
BBO-025	Unidentified	K1	1	70	38	99X/51Y	2021
BBO-026	Aves	K1	3	388	2	99X/51Y	2021
BBO-027	Pisces	K1	1	71	14	99X/51Y	2021
BBO-029	Unidentified	K2	1	22	14	99X/51Y	2021
BBO-030	Mammalia	K2	1	50	5	99X/51Y	2021
BBO-031	Pisces	K/L	1	15	30	99X/51Y	2021
BBO-035	Unidentified	L1	1	74	26	99X/51Y	2021
BBO201	Aves	L1	1	48.2	1	99X/53Y	2022
BBO202	Aves	L2	1	41.8	1	99X/53Y	2022
BBO203	Pisces	U1	1	53.7	1	99X/53Y	2022
BBO204	Pisces	KL	1	18.8	2	99X/53Y	2022
BBO205	Pisces	L2	1	47	3	99X/53Y	2022
BBO206	Pisces	Y1	1	21.5	2	99X/53Y	2022
BBO207	Pisces	L1	1	67.2	3	99X/53Y	2022
BBO208	Pisces	L/U	3	433.7	12	99X/53Y	2022
BBO209	Mammalia	K1	3	2940	6	99X/53Y	2022
BBO210	Mammalia	L1	3	2213.4	6	99X/53Y	2022
BBO211	Mammalia	L2	1	20.4	1	99X/53Y	2022
BBO212	Mammalia	Z1	1	7.7	1	99X/53Y	2022
BBO213	Mammalia	L/U	3	2072.3	14	99X/53Y	2022

Table S16 continued. Sample information of the BBM samples. Metabarcoding group (Aves, Mammalia, Pisces and Unidentified vertebrate), layer from which the bones were retrieved (Fig. 1; Fig. 2; numbers indicate mechanical layer within stratigraphic layer), number of subsamples from which DNA was extracted, total bone powder from the sample following milling, total number of countable fragments in the sample, quadrant and year the sample was excavated are presented. The number of countable fragments does not include fragments that were close to bone powder. DNA was extracted from up to three subsamples of ~110 mg where that amount was available, for a total of 78 subsamples analyzed.

BBM sample No.	Group	Layer	No. of samples	Total bone powder (mg)	Total number of countable fragments	Location	Excavation year
BBO214	Mammalia	Y1	3	7770	38	99X/53Y	2022
BBO215	Unidentified	Y1	3	5414.1	491	99X/53Y	2022
BBO216	Unidentified	Z1	2	198.8	12	99X/53Y	2022
BBO217	Unidentified	KL	3	744.3	97	99X/53Y	2022
BBO218	Unidentified	K1	3	1718	136	99X/53Y	2022
BBO219	Unidentified	L1	3	1860.1	128	99X/53Y	2022
BBO220	Unidentified	L2	2	250.4	19	99X/53Y	2022
BBO221	Unidentified	L/U	3	4655.4	233	99X/53Y	2022
BBO222	Unidentified	U1	1	7.3	1	99X/53Y	2022
BBO223	Unidentified	U3	1	13.1	3	99X/53Y	2022
BBO224	Unidentified	U9	1	1.3	1	99X/53Y	2022
BBO225	Unidentified	Q1	1	8.4	1	99X/53Y	2022

Table S17. Sample information of single bones tested for identification by metabarcoding. Bone element, layer from which the bone was retrieved (Fig. 1; Fig. 2; numbers indicate mechanical layer within stratigraphic layer), total bone powder obtained from milling, quadrant and excavation year are presented. Up to 110 mg of bone powder was used in DNA extractions.

Sample No.	Osteological identification	BBM identification	Bone	Layer	Total bone powder (mg)	Quadrant	Excavation year
NOC013	*Ursus* sp.	*Ursus* sp.	Phalanx	K1	110	98X/51Y NE	2022
NOC017	Passeriformes	Failed	Scapula	L1	12.4	99X/53Y SW	2022
NOC018	Passeriformes	Fringillidae	Ulna	K1	61.8	99X/53Y SW	2022
NOC019	Passeriformes	Failed	Tibiotarsus	Z1	8.4	98X/54Y NW	2022
NOC020	Aves	*Lagopus muta*	Phalanx	K1	10.2	99X/52Y SW	2022
NOC021	Aves	Failed	Phalanx	K1	18.7	98X/50Y NE	2022
NOC022	Alcidae	Failed	Phalanx	LU	112	99X/53Y NE	2022
NOC023	*Somateria mollissima*	Anatidae	Mandible	L1	121.4	99X/50Y SE	2021
NOC033	Alcidae	*Fratercula arctica*	Vertebrae	L1	156.8	98X/51Y NW	2022
NOC034	Anatidae	*Melanitta* sp.	Sternum	K1	110	98X/50Y NE	2022
NOC035	Anatidae	Anatidae	Scapula	Z1	230.5	99X/53Y SW	2022
NOC036	Anseriformes	*Bucephala* sp.	Vertebrae	LU	143.7	99X/54Y SW	2022
NOC037	Galliformes	Failed	Vertebrae	K1	51.4	98X/50Y NE	2022
NOC038	Mammalia	Failed	Phalanx II	U1	16.4	99X/51Y SW	2022
NOC039	*Lepus* sp.	*Lepus* sp.	Molar	Z1	80.4	100X/53Y SW	2022
NOC040	*Lepus* sp.	*Lepus* sp.	Incisor	L1	123.7	98X/52Y SW	2022

Bioinformatic analyses of BBM

Data was processed using the OBITools, package v.1.2.12 [https://pythonhosted.org/OBITools/index.html; (98)] and by following https://pythonhosted.org/OBITools/wolves.html. We used the program *illuminapairedend* to assemble forward and reverse reads before assigning samples with *ngsfilter*. Reads with quality scores <40, tags with a match <100%, primers with >3 mismatches and short reads (length <20bp) were discarded. We merged unique reads and used *obiclean* to discard singletons and identify sequencing and/or PCR artifacts. We also applied a 5% threshold ratio in order to reclassify “internal” sequences to their respective “head”. Sequences were then compared to a reference library for taxonomic assignment with the program *ecotag*. We built a reference library for each primer pair (Mamp007, Fish16S and Aves12S) with ecoPCR, a program running an *in-silico* PCR on the European Molecular Biology Laboratory (EMBL, February 2022, https://www.ebi.ac.uk/ena/browser/home) and the NCBI Taxonomy database (https://www.ncbi.nlm.nih.gov/taxonomy). The sequences of the *Lemmus lemmus* (Norwegian lemming) and the *Alexandromys oeconomus* (tundra vole) were added manually to the reference library (99).

Following analyses with OBITools, we filtered the sequences in R v.4.3.0 (https://www.r-project.org/). To minimize misidentification we kept sequences with >95% identity for fish and birds and >98% for mammals. The primer Mamp007 amplified a few species of fish and birds as well as mammals and reads with >95% identity belonging to these taxa were extracted from the mammal data and merged with the results from their respective primers (Fish16S and Aves12S). Thereafter all sequence variants were merged by taxonomic identification and we further removed taxa that had a total read count under 200 and identifications to order level or above (with the exception of Mysticeti which was further investigated due to the very high number of reads, see Table S20). A summary of the filtering steps including reads is provided in Table S18. Genera *Ovis* sp. and *Sus* sp. remained in one sample each post filtering. Though they did not appear in blanks, we removed these from the dataset as they are common contaminants. Other common contaminants such as *Bos* sp. or *Gallus* sp. were not found in the dataset and reads assigned to *Homo* sp. were not retained after applying the various filtering steps. One extraction negative control had reads identified to the Leuciscinae family. As this family has been identified as a contaminant in our previous work (8), we removed this identification from the taxa list. None of the other negative controls had sequences post-filtering. The final taxon list obtained by BBM list after filtering and taxonomic adjustments are presented in Table S19 and S20.

Table S18. Number of sequences and unique sequences remaining after each filtering step. Sequences were processed using the OBITools package v.1.2.12 [https://pythonhosted.org/OBITools/index.html (98)], and filtered in R (version 4.3.0; R https://www.r-project.org/). Asterisks indicate programs included in the OBITools package.

Filtering step	Program	Primer	Total reads		Unique sequences	
			2021	2022	2021	2022
Raw reads from sequencing	-	-	68,730,588	10,755,162	-	-
Pairwise alignment	illuminapairedend*	-	68,730,588	10,755,162	-	-
Removal of sequences with <40 quality score	obigrep*	-	64,863,530	10,368,001	-	-
Assignment of sequences to samples	ngsfilter*	Aves12S	22,445,565	1,254,454	-	-
		Fish 16S	19,455,457	1,852,432	-	-
		Mamp007	18,509,479	6,548,128	-	-
Removal of reads with <20 bp and >165 bp length and merging identical reads	obigrep, obiuniq*	Aves12S	21,474,039	1,141,126	43,386	11,990
Removal of reads with <20 bp and >150 bp length and merging identical reads	obigrep, obiuniq*	Fish16S	14,980,571	1,553,160	91,382	31,092
		Mamp007	16,206,949	5,612,569	149,142	105,975
Matching to the reference library	ecotag,obigrep*	Aves12S	21,474,039	1,141,126	43,386	11,990
		Fish16S	14,980,571	1,553,160	91,382	31,092
		Mamp007	16,206,949	5,612,569	149,142	105,975
Removal of PCR and sequencing errors	obiclean*	Aves12S	19,975,069	1,058,828	8957	3455
		Fish16S	12,851,727	1,390,743	27,286	11,973
		Mamp007	21,474,039	4,924,981	43,386	50,669
Removal of sequences with <95% ID match	R	Aves12S	-	-	1130	1025
		Fish16S	-	-	6422	3909
Removal of sequences with <98% ID match	R	Mamp007	-	-	142	193

Table S19. Number of reads per layer per taxon identified by bulk-bone metabarcoding. Layers (Fig.1; Fig. 2) include numbers indicating the mechanical layer within the stratigraphic layer. Original taxonomic identifications are presented, and identifications that were later manually adjusted or removed are marked with an asterisk (see Table S20).

	Layer													
Species ID	Y1	Z1	K1	K2	K/L	L1	L2	L/U	U1/2	U3/4	U5/6	U7/8	U9/10	Q1
**Aves**														
Anatidae	0	70,214	870,548	0	109,614	7,740,545	1,032,130	55,660	0	0	0	0	0	0
Somateria sp.	0	0	0	0	0	0	432	0	0	0	0	0	0	0
Somateria spectabilis	0	0	0	0	0	0	1486	0	0	0	0	0	0	0
Bucephala sp.	0	0	0	0	0	210	2063	1117	0	0	0	0	0	0
Clangula hyemalis	0	0	0	0	0	1337	0	0	0	0	0	0	0	0
*Melanitta deglandi**	0	0	40,645	0	0	0	0	0	0	0	0	0	0	0
Aythya sp.	0	0	0	0	0	293,986	0	0	0	0	0	0	0	0
Tetraoninae	0	0	0	0	0	627,926	193,108	0	0	0	0	0	0	0
Lagopus sp.	22,954	3677	133,899	0	0	654	363,692	74,227	0	0	0	0	0	0
Lagopus muta	0	0	0	0	0	0	390	0	0	0	0	0	0	0
Fulmarus glacialis	0	0	0	0	0	0	33,622	0	0	0	0	0	0	0
Cepphus grylle	0	0	0	0	0	742	2,426,710	0	0	0	0	0	0	0
Uria sp.*	38,614	0	2042	0	0	0	85,841	398,764	0	0	0	0	0	0
Uria aalge	0	0	1,702,215	0	0	0	0	0	0	0	0	0	0	0
Alca torda	0	0	2050	0	0	0	0	0	0	0	0	0	0	0
Fratercula arctica	0	0	0	0	0	92,351	0	0	0	0	0	0	0	0
Laridae	0	1146	0	0	0	0	60,635	0	0	0	0	0	0	0
Accipitrinae*	0	0	0	0	0	0	770,175	86,909	0	0	0	0	0	0
Buteo sp.	0	0	0	0	0	0	3643	0	0	0	0	0	0	0
Haliaeetus sp.*	0	0	0	0	0	0	1654	0	0	0	0	0	0	0
Milvus sp.*	0	0	0	0	0	2677	0	0	0	0	0	0	0	0
Fringillidae	0	0	0	0	0	1979	0	0	0	0	0	0	0	0
Corvidae	0	0	0	0	0	0	6478	0	0	0	0	0	0	0
Corvus sp.*	0	0	0	0	0	0	1194	0	0	0	0	0	0	0
Podicipedidae	0	0	0	0	0	0	201	0	0	0	0	0	0	0
Phalacrocorax sp.*	0	0	0	0	0	0	16,789	1,035,614	0	0	0	0	0	0
Gruidae	0	0	0	0	0	0	723	0	0	0	0	0	0	0
**Mammalia**														
Lepus sp.	0	63,124	0	0	0	40,080	0	30,993	0	0	0	0	0	0
Dicrostonyx torquatus	0	0	1,092,105	58,613	0	29,159	4,256,075	236,042	33,442	19,305	0	0	0	0
Rangifer tarandus	28,223	25,400	630,661	127,621	10,525	209,073	648,271	594,163	0	6771	0	0	0	0
Phocoena phocoena	0	1596	0	0	3475	0	46,729	201	0	0	0	0	0	0
Mysticeti*	0	0	55,920	0	0	17,418	0	82,956	12,261	0	0	0	0	0
Balaenoptera sp.	0	0	5293	0	0	0	0	0	0	0	0	0	0	0
Balaena mysticetus	0	0	376,269	0	39,608	0	107,477	0	0	0	0	0	0	0
Ursus sp.*	34,706	0	231,340	27,003	0	5314	885,446	318,731	0	0	0	0	0	0
Odobenus rosmarus	197,877	0	774,955	0	6351	3027	61,595	11,941	0	0	0	0	0	0
Vulpes lagopus	0	0	0	0	0	0	22,528	315,040	0	0	0	0	0	0
Canis lupus	0	0	0	0	0	0	0	1953	0	0	0	0	0	0
Erignathus barbatus	0	0	66,602	0	0	0	0	0	0	0	0	0	0	0
Pusa hispida	0	0	0	0	0	4633	0	0	0	0	0	0	0	0
**Pisces**														
Gadidae*	0	0	5,192,478	0	6127	252,924	1,046,910	225,195	0	0	0	0	0	0
Gadoidei	0	0	463,008	0	3707	0	56,340	0	0	0	0	0	0	0
Melanogrammus aeglefinus	0	0	98,423	0	87,770	620,460	39,805	142,876	50,140	0	0	0	0	0
Molva molva	0	0	0	0	0	0	0	0	0	0	0	0	0	46,040
*Trachinus draco**	0	0	0	0	0	0	0	224	0	0	0	0	0	0
Sebastinae*	0	0	15,359	1,610,009	0	0	669,068	0	0	0	0	0	0	0
*Sebasticus marmoratus**	0	0	0	332,781	0	0	0	0	0	0	0	0	0	0
Salmonidae	0	0	0	0	0	0	208	28,388	0	0	0	0	0	0
Thymallus sp.*	0	0	0	0	0	0	0	3181	0	0	0	0	0	0
Salvelinus sp.	0	0	0	0	0	0	335	0	0	0	0	0	0	0
Alburnus alburnus	0	0	0	0	0	4446	2881	0	0	0	0	0	8279	0
Cyprinidae	0	0	0	0	0	0	0	0	39,806	0	0	0	0	0

Adjustments of taxonomic identification

The complete taxa list was reviewed by taxonomic experts with a good understanding of Northern European fauna. Liselotte M. Takken Beijersbergen, senior engineer at the University Museum of Bergen and Thijs van Kolfschoten, professor emeritus in archeology at the University of Leiden, reviewed the mammals. Ingvar Byrkjedal, professor emeritus and Nicolas Straube, associate professor and curator of ichthyology from the Department of Natural History at the University Museum of Bergen reviewed the fish. Samuel J. Walker at the University of Oslo and Bournemouth University reviewed the birds. Sequences of unexpected taxa and identification higher than species level were also assessed with the NCBI Nucleotide BLAST tool as a complementary analysis. For cases with multiple sequence variants per taxon we only analyzed the one with the highest read count. We did a standard nucleotide blast, optimizing for highly similar sequences (megablast), and applying the same identify threshold as in the ObiTools analyses (95% for fish and birds). We restricted to sequences with 100% query cover and looked at the first 500 matches. Only matches belonging to the 16S ribosomal RNA gene for sequences amplified by primers Mamp007 and Fish16S, and to the 12S for Aves12S were considered because our ObiTools database was built by an *in silico* PCR using primers matching highly conserved sequences within those genes. Based on this assessment the taxonomic level of some identifications was adjusted (Table S20). To account for any uncertainty in identifications we added cf. to all species that were identified following adjustments as justified in Table S20. After final adjustments the aDNA BBM and osteological taxa lists were combined (Table S21).

Table S20. Manually adjusted or removed taxonomic identifications. Justifications for manual adjustment and removal of taxa identified from the bulk-bone metabarcoding data using the OBITools package v. 1.2.12 (98).

EcoTag ID	Adjusted ID	Justification
Melanitta deglandi	*Melanitta* sp.	Breeds in North America and winters along the coasts of the Pacific and Atlantic oceans (100). It is more likely that the Arne Qvamgrotta reads represents one of the native European species of either *Melanitta nigra* or *Melanitta fusca*. Therefore, we adjusted this identification to the genus *Melanitta*.
Uria sp.	cf. *Uria lomvia*	Results of the NCBI-Blast show a 100% query cover and sequence identity to the 12S of *Uria lomvia*. The next best matches for the 12S gene have 98.04% identity. Not all sequences were identified to species level, so we add cf., and we also keep *Uria* sp. in the identification list.
Accipitrinae	cf. *Haliaeetus albicilla*	100% query cover and sequence identity to *Haliaeetus albicilla*, *Haliaeetus leucocephalus*, *Haliaeetus pelagicus* and *Spilornis cheela.* Given the current geographic distribution, we adjust to *H. albicilla*. Not all sequences were identified to species level, so we add cf., and we also keep *Haliaeetus* sp. in the identification list.
Haliaeetus sp.	*Haliaeetus albicilla*	100% query cover and sequence identity to the 16S of *Haliaeetus albicilla* and *Haliaeetus leucocephalus*. Given the current geographic distribution, we adjust to *H. albicilla*.
Milvus sp.	cf. *Milvus migrans*	100% query cover and sequence identity to *Milvus migrans*. Both *M. migrans* and *M. milvus* that have possible geographical ranges including Northern Europe and Scandinavia were in the database. Not all sequences were identified to species level, so we add cf., and we also keep *Milvus* sp. in the identification list.
Corvus sp.	cf. *Corvus corax*	100% query cover and 100% identity to the 16S of *Corvus corax* and *Corvus cryptoleucus*. Given that the geographic distribution of *C. cryptoleucus* is in North America we adjust to *C. corax.*
Phalacrocorax sp.	*Phalacrocorax carbo*	*Phalacrocorax carbo* is the only species in this genus found in Europe (101). Therefore, we adjust to *P. carbo*.
Mysticeti	cf. *Balaenoptera musculus*	The sequence has a 100% query cover and 98.67% identity to *Balaenoptera musculus*, and the next best match is 98.6% (to *Balaenoptera omurai* which is found in the Indian Ocean and Western Pacific). All four species with relevant geographic range were in the database. We therefore add cf. *B. musculus* to the list of identified taxa.
Ursus sp.	*Ursus maritimus*	The *Ursus* sample from Arne Qvamgrotta clusters with *Ursus maritimus* in the mitogenome phylogeny generated in BEAST v1.10.4 (Fig. S14). Therefore, we adjusted this identification to *Ursus maritimus*.
Gadidae	cf. *Gadus morhua*	100% query cover and sequence identity to *Gadus morhua*. Sequence GH00000007 has a count of 1,330,352 and was amplified by primer Mamp007. Not all sequences were identified to species level, so we add cf., and we also keep Gadidae in the identification list.
Sebasticus marmoratus	Sebastinae	Tropical species distributed in the Western Pacific. Therefore, we adjusted the identification to the family of Sebastinae which has many taxa in the Atlantic.
Sebastinae	*Sebastes norvegicus*	100% query cover and sequence identity to species of the *Sebastes* genus, including *Sebastes norvegicus*. Most likely belongs to *S. norvegicus* as that species was identified by osteology, but because not all sequence variants are identified to *S. norvegicus* we also keep Sebastinae in the identification list.

Table S20 continued. Manually adjusted or removed taxonomic identifications. Justifications for manual adjustment and removal of taxa identified from the bulk-bone metabarcoding data using the OBITools package v. 1.2.12 (98).

EcoTag ID	Adjusted ID	Justification
Trachinus draco	*Trachinus* sp.and cf. *Trachinus draco*	The Obitools identification is 96.25% to *T. draco*. NCBI-Blast shows a match to the North American species *Prosopium williamsoni* (100% query cover; 97.5% identity). The next best match is *T. draco* (100% query cover and 96.25%) followed by species, the Salmonidae family (100% query match; 96.20% sequence identity). One species potentially within nearby geographic range (current suitable habitat for this species includes the western coast of Norway) was missing from the database for the 16S (*Trachinus radiatus*; Starry weaver). We therefore add cf. to the species identification and add the genus to our taxa list as a precaution.
Thymallus sp.	cf. *Thymallus arcticus*	All tree sequence variants have 100% query cover and 98.73% sequence identity to the 16S of *Thymallus arcticus*. The next best matches have a 97.47% identity. All seven species belonging to this genus were in the database, as a precaution we add cf.

Text S7. Comparison of identification methods

Comparative morphological analysis identified 350 fragments (5.7%; Table S17) to at least family level, representing 16 taxa from 14 families, 13 genera and seven species. The remaining 5841 (94.3%) bone fragments were regarded unidentifiable by comparative osteology and grouped by class: Mammalia (769 Fragments), Pisces (270 Fragments), Aves (29 Fragments) and unidentifiable vertebrates (4773 Fragments). A total of 79.5 million raw sequence reads (Table S19) were generated for the aDNA BBM analyses. Initial filtering and taxonomic assignment gave a total of 37073 sequence variants for the Fish16S primer, 132,802 for Mamp007 and 20062 for Aves12S. We filtered the variants based on identity, requiring 98% match for mammals and 95% match for fish and birds. Out of these filtered variants we identified 43 taxa, including 28 families, 35 genera and 31 species (including the *U. maritimus* identified through phylogenetic analyses; Table S19). Identification of some taxa was adjusted (Table S20).

Overall, more taxa were identified for all taxonomic classes using aDNA BBM compared to comparative osteology (Table S21). Combined, the two methods identified 46 taxa representing a total of 30 families, 37 genera and 33 species (Table S21). The class Aves accounted for 23 taxa, Mammalia comprised 13 taxa, Pisces 10 taxa. A total of seven taxa, including five mammals and two fish were identified using both methods (Table S21). By contrast, three taxa, including for example *Alexandromys oeconomus* (tundra vole) and the Cottidae (sculpins) family were exclusively identified by osteological methods, whilst 36 taxa, comprising mainly birds, fish and sea mammal species, were exclusively detected by aDNA BBM.

Table S21. Taxa identified at Arne Qvamgrotta, indicating by which method (osteology or aDNA BBM) it was identified (see also Fig. 4).

Species ID	Common name	Osteology	aDNA BBM
**Aves**			
Anatidae	Ducks & geese	X	X
Somateria sp.	Eider sp.	-	X
Somateria mollissima	Common eider	X	-
Somateria spectabilis	King eider	-	X
Bucephala sp.	Goldeneye sp.	-	X
Clangula hyemalis	Long-tailed duck	-	X
Melanitta sp.	Scoter sp.	-	X
Aythya sp.	Diving duck sp.	-	X
Tetraoninae	Grouse	X	X
Lagopus sp.	Ptarmigan sp.	X	X
Lagopus muta	Rock ptarmigan	-	X
Fulmarus glacialis	Northern Fulmar	-	X
Alcidae	Auks	X	-
Cepphus grylle	Black guillemot	-	X
Uria sp.	Guillemot sp.	X	X
cf. *Uria lomvia*	cf. Brünnich's guillemot	-	X
Uria aalge	Common guillemot	-	X
Alca torda	Razorbill	-	X
Fratercula arctica	Atlantic puffin	-	X
Laridae	Gulls	-	X
Accipitrinae	'True' hawks	-	X
Buteo sp.	Hawks & Buzzards sp.	-	X
Haliaeetus albicilla	White-tailed eagle	-	X
Milvus sp.	Kite sp.	-	X
cf. *Milvus migrans*	cf. Black kite	-	X
Passeriformes	Perching birds	X	-
Fringillidae	Finches	-	X
Corvidae	Crows, ravens, rooks, etc.	-	X
cf. *Corvus corax*	cf. Common raven	-	X
Podicipedidae	Grebes	-	X
Phalacrocorax carbo	Great cormorant	-	X
Gruidae	Cranes	-	X
**Mammalia**			
Lepus sp.	Hares	X	X
Rodentia	Rodents	X	-
Cricetidae	Family within Rodentia	X	-
Arvicolinae	Voles, lemmings and muskrats	X	-
Microtus sp.	Vole sp.	X	-
cf. *Alexandromys oeconomus*	cf. Tundra vole	X	-
Alexandromys oeconomus	Tundra vole	X	-
cf. *Dicrostonyx torquatus*	cf. Collared lemming	X	-
Dicrostonyx torquatus	Collared lemming	X	X
Cervidae	Deer	X	-
cf. *Rangifer tarandus*	cf. Reindeer	X	-
Rangifer tarandus	Reindeer	X	X
Phocoena phocoena	Harbour porpoise	-	X
Balaenoptera sp.	Rorquals	-	X
cf. *Balaenoptera musculus*	cf. Blue whale	-	X
Balaena mysticetus	Bowhead whale	-	X
cf. *Ursus* sp.	cf. Bear sp.	X	-
Ursus sp.	Bear sp.	X	X
*Ursus maritimus**	Polar bear	*	*
Odobenus rosmarus	Walrus	-	X
Vulpes lagopus	Arctic fox	X	X

*The identification of *U. maritimus* was achieved by building a mitogenome phylogeny (Fig. 3; Fig. S14).

Table S21 continued. Taxa identified at Arne Qvamgrotta, indicating by which method (osteology or aDNA BBM) it was identified.

Species ID	Common name	Osteology	aDNA BBM
Canis lupus	Grey wolf	**X**	**X**
Erignathus barbatus	Bearded seal	**-**	**X**
Pusa sp.	Earless seal sp.	**X**	**-**
Pusa hispida	Ringed seal	**-**	**X**
**Pisces**			
Gadidae	Cods & haddocks	**X**	**X**
Gadoidei	Ray-finned fish	**-**	**X**
cf. *Gadus morhua*	cf. Atlantic cod	**-**	**X**
Melanogrammus aeglefinus	Haddock	**-**	**X**
Molva molva	Common ling	**-**	**X**
Trachinus sp.	Weever sp.	**-**	**X**
cf. *Trachinus draco*	cf. Greater weever	**-**	**X**
Sebastinae	Rockfishes	**-**	**X**
Sebastes norvegicus	Golden redfish	**X**	**X**
Salmonidae	Family of ray-finned fish	**-**	**X**
cf. *Thymallus arcticus*	cf. Arctic grayling	**-**	**X**
cf. *Salvelinus* sp.	cf. Char sp.	**X**	**-**
Salvelinus sp.	Char sp.	**-**	**X**
Cottidae	Sculpins	**X**	**-**
Alburnus alburnus	Common bleak	**-**	**X**
Cyprinidae	Minnows and carps	**-**	**X**

*The identification of *U. maritimus* was achieved by building a mitogenome phylogeny (Fig. 3; Fig. S14).

Text S8. Identification of shells

Shell fragments were recovered from the sieved sediments (2–4 mm mesh) and stored separately from the bone fragments. Fragments were generally very small with no complete shells having been recovered, resulting in much of the material being un-diagnostic and therefore unidentifiable. Total shell material per layer was weighed and larger shell fragments were identified (Table S22).

Table S22. Identification of shell from Arne Qvamgrotta. Presence of shell species where X represents presence within a given layer. An overall shell weight of all fragments per layer is given.

Species ID	Z	Y	K	L	L/U	U	Q	W
Mytilus sp.	X	X	X	X	X	X	X	-
Littorina sp.	X	X	X	X	X	-	-	-
Cirripedia	X	X	X	X	X	X	-	-
Balanoide	X	-	X	-	-	X	-	-
Total weight (g)	4.77	0.62	12.79	1.88	0.56	6.22	0.18	0.13

Text S9. Pollen analyses

Samples for pollen analyses were collected from both Arne Qvamgrotta (n=29) and Nygrotta (n=21). Eight samples from the lower layers (Units 8–6) of Arne Qvamgrotta were selected for initial analyses, based on the samples' proximity to the bone-bearing layers (Fig. S4). From each selected pollen sample 1 cm^3 of material was extracted for pollen analysis except for sample AQP2 which contained only 0.8 cm^3 of material. The samples were left in 10% HCl for a few minutes to remove $CaCO_3$ before adding four *Lycopodium*-tablets (nr. 100320201) (102). These tablets are added in a known quantity for calculating the concentration of pollen deposited per cubic centimeter. The samples were prepared following standard procedures (103) where 10% KOH was added to remove humic acids, and 48% HF heated to 100ºC was added to remove minerogenic particles. The samples were not acetolysed as there was very little material left after the HF treatment. All samples were dyed using fuchsine and subsequently stored in glycerol. A Zeiss Imager.A1 microscope with phase contrast and 40x and 63x magnification was used for the analysis. Charcoal dust over 10 µm was counted.

None of the samples yielded any pollen, but charcoal dust was detected. Sample AQP6 from the bottom of Layer L contained wood fibers of what is possibly Pinaceae (the pine family; Table S23; Fig. S15a). This sample also contained a lot more charcoal dust compared to the other analyzed samples (Table S23; Fig. S15b). None of the charcoal fragments displayed any identifiable traits such as intervessel pits or fiber structures. All samples were highly minerogenic, mostly consisting of sand and small gravel, and in such sediments, pollen is easily washed-out during periods of water flow.

Table S23. Results of the pollen analyses in Arne Qvamgrotta. Sample ID and layer from which the sample was collected. Pollen was not detected in the samples. Number of *Lycopodium* spores counted after adding four *Lycopodium* tablets is presented, and charcoal dust (>10 µm) and wood fibers were identified.

Sample No.	Layer	No. of *Lycopodium* spores	Charcoal dust	cf. Pinaceae, wood fiber
AQP8	J	33	600	0
AQP7	K	43	250	0
AQP6	L	935	4845	2
AQP5	U	54	75	0
AQP4	U	21	124	0
AQP3	U	7	140	0
AQP2	U	196	80	0
AQP1	Q	180	415	0

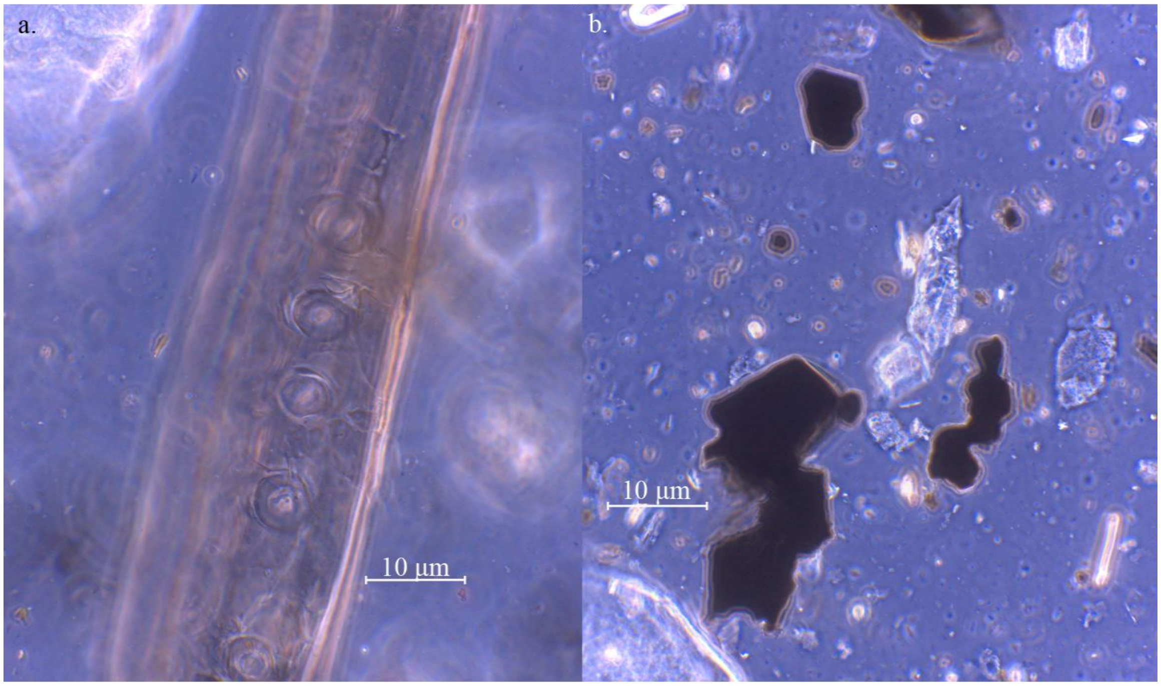

Figure S15. Possible wood fiber and charcoal dust from sample AQP6. **a)** Possible wood fiber showing pits identified as Pinaceae (pine) type, **b)** charcoal dust particles. Scale of 10 µm.

Text S10. sedaDNA analyses

Samples for sedaDNA analyses were collected from Arne Qvamgrotta while wearing personal protective equipment (hairnets, facemasks, arm covers and gloves) and using sterile instruments. Sampling tubes and scalpel holders were UV irradiated in preparation for the sampling. Before samples were taken, ~0.5 cm of surface material was removed by two consecutive cuts made with sterile scalpels. Samples were collected by pushing a collection tube into the cleaned sediment surface. Samples were stored in a freezer and transported on ice to the ancient DNA facilities.

DNA extraction and plant metabarcoding was done in the ancient DNA facilities at Tromsø Museum, UiT. DNA was extracted from 12 samples (Fig. S4; Table S23), two negative controls and one positive control (ancient DNA sample that is known to have good DNA quality). The DNA extraction was done from 0.25–0.35 g sediments using the PowerSoil PowerLyzer kit following Alsos et al. (104). We amplified the P6-loop of *trn*L (UAA) intron using the g-h primers (105). A total of eight PCR replicates were obtained of each DNA extract and sequencing was performed on an Illumina NextSeq Platform following Alsos et al. (104). We matched DNA sequences to four reference libraries: PhyloNorway (106), PhyloAlps (107), ArcborBryo (108–110), and EMBL release 143. Only sequences with a 100% match to at least one of these reference libraries were kept. One of the negative controls contained *Vaccinium* and *Apioideae* in one PCR replicate. These are known background contaminants in the ancient DNA facilities at Tromsø Museum due to studies on *Vaccinium uliginosum* (111) and *Heracleum* (112).

Only eight sequences were identified in the 12 samples, and all had poor or no replication and were identified as common contaminants. In contrast, 69 taxa with good replication were identified in the positive control showing that the laboratory procedures had been successful.

There are three possible reasons for the lack of plant DNA: 1) plant DNA was there, but it was not extracted and/or amplified due to inhibition, 2) plant DNA was there, but their fragmented length was too short for metabarcoding (fragment length <90 bp), or 3) there was no plant DNA. The sediments contained clay which generally binds DNA well and there was no humic material

observed that could inhibit DNA amplification. We therefore do not think that inhibition prevented DNA extraction and/or amplification. While DNA is known to degrade over time, plant metabarcoding has been successful for samples of similar age on Ellesmere Island (113) and many sites for samples with ages around 50 ka [e.g., (109)]. Given the exceptional stable temperatures in caves, and the good preservation of animal bone DNA in Arne Qvamgrotta, we do not think that fragmentation prevented DNA amplification. It is therefore most likely that the samples did not contain any plant DNA. Plant records are poor or scattered from caves except for caves that have large openings or where plant material was actively brought into the caves by humans or animals [e.g., (114)]. In Arne Qvamgrotta the analyzed sediments are ~13 m from the present opening in Nygrotta, and the probability that plant material was blown or washed into the cave appears low. This result is analogous with the lack of pollen grains in the sediments of the cave (see Pollen section above).

Table S24. Samples analyzed for sedaDNA analyses**.** Sample number and layer information are provided.

Sample No.	Layer
NOS202	U (Lower)
NOS203	U (Lower)
NOS204	U (Middle)
NOS205	U (Upper)
NOS206	L
NOS207	K
NOS208	Z
NOS209	J (Lower)
NOS226	X
NOS227	W
NOS228	N
NOS229	S

SI References

1. H. Nese, “Sedimentologisk utvikling av grottene i Kjøpsvik–med hovudvekt på Stronsteinsholesystemet,” University of Bergen, Bergen. (1996).
2. Å. Lauritsen, “Geomorfologisk utvikling av grottene i Kjøpsvik - korrelert med landskapsutviklingen i Tysfjordområdet,” University of Bergen, Bergen. (1997).
3. J. Mangerud, *et al.*, Did the Eurasian ice sheets melt completely in early Marine Isotope Stage 3? New evidence from Norway and a synthesis for Eurasia. *Quat Sci Rev* **311**, 108136 (2023).
4. T. R. Hester, H. J. Shafer, K. L. Feder, *Field methods in archaeology* (Routledge, 2016).
5. C. Renfrew, P. Bahn, *Archaeology: Theories, Methods and Practice*, 8th Ed. (Thames and Hudson, 2020).
6. C. Orton, *Sampling in archaeology* (Cambridge University Press, 2000).
7. S. Payne, “Partial recovery and sample bias: the results of some sieving experiments” in *Papers in Economic Prehistory*, E. S. Higgs, Ed. (Cambridge University Press, 1972), pp. 49–64.
8. A. Boilard, *et al.*, Ancient DNA and osteological analyses of a unique paleo-archive reveal Early Holocene faunal expansion into the Scandinavian Arctic. *Sci Adv* **10**, 3032 (2024).
9. N. Eyles, C. H. Eyles, A. D. Miall, Lithofacies types and vertical profile models; an alternative approach to the description and environmental interpretation of glacial diamict and diamictite sequences. *Sedimentology* **30**, 393–410 (1983).
10. R. Sohbati, A. Murray, L. Lindvold, J.-P. Buylaert, M. Jain, Optimization of laboratory illumination in optical dating. *Quat Geochronol* **39**, 105–111 (2017).
11. A. Murray, L. Helsted, M. Autzen, M. Jain, J.-P. Buylaert, Measurement of natural radioactivity: Calibration and performance of a high-resolution gamma spectrometry facility. *Radiat Meas* **120**, 215–220 (2018).

12. A. Murray, R. Marten, A. Johnston, P. Martin, Analysis for naturally occuring radionuclides at environmental concentrations by gamma spectrometry. *J Radioanal Nucl Chem* **115**, 263–288 (1987).
13. A. J. Cresswell, J. Carter, D. C. W. Sanderson, Dose rate conversion parameters: Assessment of nuclear data. *Radiat Meas* **120**, 195–201 (2018).
14. D. J. Huntley, M. R. Baril, The K content of the K-feldspars being measured in optical dating or in thermoluminescence dating. *Ancient TL* **15**, 11–13 (1997).
15. D. J. Huntley, R. G. V. Hancock, The Rb contents of the K-feldspar grains being measured in optical dating. *Ancient TL* **19**, 43–46 (2001).
16. H. Zhao, S. H. Li, Internal dose rate to K-feldspar grains from radioactive elements other than potassium. *Radiat Meas* **40**, 84–93 (2005).
17. J. R. Prescott, J. T. Hutton, Cosmic ray contributions to dose rates for luminescence and ESR dating: large depths and long-term time variations. *Radiat Meas* **23**, 497–500 (1994).
18. A. Murray, *et al.*, Optically stimulated luminescence dating using quartz. *Nature Reviews Methods Primers* **1** (2021).
19. J. Tukey, *Exploratory data analysis* (1977).
20. V. Hansen, *et al.*, Towards the origins of over-dispersion in beta source calibration. *Radiat Meas* **120**, 157–162 (2018).
21. M. Autzen, C. E. Andersen, M. Bailey, A. S. Murray, Calibration quartz: an update on dose calculations for luminescence dating. *Radiat Meas* **157**, 106828 (2022).
22. G. A. T. Duller, Distinguishing quartz and feldspar in single grain luminescence measurements. *Radiat Meas* **37**, 161–165 (2003).
23. A. G. Wintle, A. S. Murray, A review of quartz optically stimulated luminescence characteristics and their relevance in single-aliquot regeneration dating protocols. *Radiat Meas* **41**, 369–391 (2006).
24. K. J. Thomsen, A. S. Murray, M. Jain, L. Bøtter-Jensen, Laboratory fading rates of various luminescence signals from feldspar-rich sediment extracts. *Radiat Meas* **43**, 1474–1486 (2008).
25. J. P. Buylaert, *et al.*, A robust feldspar luminescence dating method for Middle and Late Pleistocene sediments. *Boreas* **41**, 435–451 (2012).
26. R. N. Kurbanov, *et al.*, A detailed luminescence chronology of the Lower Volga loess-palaeosol sequence at Leninsk. *Quat Geochronol* **73** (2022).
27. M. H. Riis, *et al.*, Middle and Late Holocene relative sea level changes and coastal development at Rugård, Denmark. *Boreas* **53**, 56–70 (2024).
28. A. S. Murray, K. J. Thomsen, N. Masuda, J.-P. Buylaert, M. Jain, Identifying well-bleached quartz using the different bleaching rates of quartz and feldspar luminescence signals. *Radiat Meas* **47**, 688–695 (2012).
29. H. Alexanderson, A. S. Murray, Luminescence signals from modern sediments in a glaciated bay, NW Svalbard. *Quat Geochronol* **10**, 250–256 (2012).
30. S. E. Lowick, M. Trauerstein, F. Preusser, Testing the application of post IR-IRSL dating to fine grain waterlain sediments. *Quat Geochronol* **8**, 33–40 (2012).
31. J. P. Buylaert, *et al.*, Luminescence dating of the PASADO core 5022-1D from Laguna Potrok Aike (Argentina) using IRSL signals from feldspar. *Quat Sci Rev* **71**, 70–80 (2013).
32. C. Bronk Ramsey, Bayesian analysis of radiocarbon dates. *Radiocarbon* **51**, 337–360 (2009).
33. P. J. Reimer, *et al.*, The IntCal20 Northern Hemisphere radiocarbon age calibration curve (0–55 cal kBP). *Radiocarbon* **62**, 725–757 (2020).
34. T. J. Heaton, *et al.*, Marine20—the marine radiocarbon age calibration curve (0–55,000 cal BP). *Radiocarbon* **62**, 779–820 (2020).
35. J. Mangerud, S. Gulliksen, Apparent radiocarbon ages of recent marine shells from Norway, Spitsbergen, and Arctic Canada. *Quat Res* **5**, 263–273 (1975).
36. P. J. Reimer, R. W. Reimer, A marine reservoir correction database and on-line interface. *Radiocarbon* **43**, 461–463 (2001).
37. S. E. Lauritzen, H. Nese, R. W. Lie, Å. Lauritsen, R. Løvlie, Interstadial/interglacial fauna from Norcemgrotta, Kjøpsvik, north Norway. *Karst Waters Institute Special Publication 2* 89–92 (1996).
38. G. Kyrle, *Grundriß der theoretischen Speläologie*, 1st Ed. (Österreichies Bundeshöhlenkommision, 1923).
39. W. Dreybrodt, *Processes in karst systems: physics, chemistry, and geology* (Springer Verlag, 1988).
40. C. Hillaire-Marcel, C. Causse, The Late Pleistocene Laurentide Glacier: Th/U dating of its major fluctuations and δ18O range of the ice. *Quat Res* **25**, 312–329 (1989).
41. C. Hillaire-Marcel, C. Causse, Chronologie Th/U des concrétions calcaires des varves du lac glaciaire de Deschaillons (Wisconsinien inférieur). *Can J Earth Sci* **26**, 1041–1052 (1989).
42. S. E. Lauritzen, R. Ø. Skoglund, "Glacier ice-contact speleogenesis in marble stripe karst" in *Treatise of Geomorphology, Vol.6: Karst Geomorphology*, A. Frumkin, Ed. (Elsevier, 2013), pp. 363–396.
43. H. P. Schwarcz, A. G. Latham, Dirty calcites 1. Uranium-series dating of contaminated calcite using leachates alone. *Chem Geol* **80**, 35–43 (1989).
44. J. L. Bischoff, J. A. Fitzpatrick, U-series dating of impure carbonates: an isochron technique using total-sample dissolution. *Geochim Cosmochim Acta* **55**, 543–554 (1991).
45. W. Przybylowicz, H. P. Schwarcz, A. G. Latham, Dirty calcites 2. Uranium-series dating of artificial calcite-detritus mixtures. *Chem Geol* **86**, 161–178 (1991).
46. M. Ivanovich, R. Harmon, *Uranium-series disequilibrium: applications to earth, marine, and environmental sciences*, 2nd Ed. (Clarendon Press, 1992).
47. T. L. Ku, Z. C. Liang, The dating of impure carbonates with decay-series isotopes. *Nuclear Instruments and Methods in Physics Research* **223**, 563–571 (1984).
48. S. Luo, Ku T-.L., U-series isochron dating: A generalized method employing total-sample dissolution. *Geochim Cosmochim Acta* **55**, 555–564 (1991).

49. E. P. Horwitz, *et al.*, Separation and preconcentration of uranium from acidic media by extraction chromatography. *Anal Chim Acta* **266**, 25–37 (1992).
50. J. J. La Rosa, *et al.*, Separation of actinides, cesium and strontium from marine samples using extraction chromatography and sorbents. *J Radioanal Nucl Chem* **248**, 765–770 (2001).
51. G. Surányi, "Development of uranium series dating method based on the measurement of 230Th/234U ratio," ELTE University, Budapest. (2005).
52. P. Vermeesch, IsoplotR: A free and open toolbox for geochronology. *Geoscience Frontiers* **9**, 1479–1493 (2018).
53. A. Martínez-Aguirre, J. M. Alcaraz-Pelegrina, J. Rodríguez-Vidal, U/Th dating of impure carbonates: 230Th/232Th activity ratios in detrital material. *J Radioanal Nucl Chem* **321**, 71–81 (2019).
54. S. Boessenkool, *et al.*, Combining bleach and mild predigestion improves ancient DNA recovery from bones. *Mol Ecol Resour* **17**, 742–751 (2017).
55. J. D. Kapp, R. E. Green, B. Shapiro, A fast and efficient single-stranded genomic library preparation method optimized for ancient DNA. *Journal of Heredity* **112**, 241–249 (2021).
56. T. V. der. Valk, *et al.*, Million-year-old DNA sheds light on the genomic history of mammoths. *Nature* **591**, 265–269 (2021).
57. V. E. Kutschera, *et al.*, GenErode: a bioinformatics pipeline to investigate genome erosion in endangered and extinct species. *BMC Bioinformatics* **23**, 1–17 (2022).
58. P. Larsson, *et al.*, Consequences of past climate change and recent human persecution on mitogenomic diversity in the arctic fox. *Philosophical Transactions of the Royal Society B* **374**, 20190212 (2019).
59. T. S. Korneliussen, A. Albrechtsen, R. Nielsen, ANGSD: Analysis of Next Generation Sequencing Data. *BMC Bioinformatics* **15**, 1–13 (2014).
60. R. C. Edgar, MUSCLE: multiple sequence alignment with high accuracy and high throughput. *Nucleic Acids Res* **32**, 1792–1797 (2004).
61. M. A. Suchard, *et al.*, Bayesian phylogenetic and phylodynamic data integration using BEAST 1.10. *Virus Evol* **4** (2018).
62. M. Hasegawa, H. Kishino, T. aki Yano, Dating of the human-ape splitting by a molecular clock of mitochondrial DNA. *J Mol Evol* **22**, 160–174 (1985).
63. Z. Yang, N. Goldman, A. Friday, Comparison of models for nucleotide substitution used in maximum-likelihood phylogenetic estimation. *Mol Biol Evol* **11**, 316–324 (1994).
64. D. Darriba, G. L. Taboada, R. Doallo, D. Posada, jModelTest 2: more models, new heuristics and high-performance computing. *Nat Methods* **9**, 772–772 (2012).
65. R. E. Green, *et al.*, A complete Neandertal mitochondrial genome sequence determined by high-throughput sequencing. *Cell* **134**, 416–426 (2008).
66. E. Lord, *et al.*, Population dynamics and demographic history of Eurasian collared lemmings. *BMC Ecol Evol* **22** (2022).
67. S. Brace, *et al.*, Serial population extinctions in a small mammal indicate Late Pleistocene ecosystem instability. *Proceedings of the National Academy of Sciences* **109**, 20532–20536 (2012).
68. E. Palkopoulou, *et al.*, Synchronous genetic turnovers across Western Eurasia in Late Pleistocene collared lemmings. *Glob Chang Biol* **22**, 1710–1721 (2016).
69. M. Schubert, *et al.*, Characterization of ancient and modern genomes by SNP detection and phylogenomic and metagenomic analysis using PALEOMIX. *Nat Protoc* **9**, 1056–1082 (2014).
70. S. Lindgreen, AdapterRemoval: Easy cleaning of next-generation sequencing reads. *BMC Res Notes* **5**, 1–7 (2012).
71. H. Li, Aligning sequence reads, clone sequences and assembly contigs with BWA-MEM. *arXiv preprint arXiv* 1–3 (2013).
72. C. Lindqvist, *et al.*, Complete mitochondrial genome of a Pleistocene jawbone unveils the origin of polar bear. *PNAS* **107**, 5053–5057 (2010).
73. M. S. Wang, *et al.*, A polar bear paleogenome reveals extensive ancient gene flow from polar bears into brown bears. *Nat Ecol Evol* **6**, 936–944 (2022).
74. H. Li, *et al.*, The sequence alignment/map format and SAMtools. *Bioinformatics* **25**, 2078–2079 (2009).
75. P. Danecek, *et al.*, The variant call format and VCFtools. *Bioinformatics* **27**, 2156–2158 (2011).
76. I. S. Feinauer, *et al.*, Heterochronous mitogenomes shed light on the Holocene history of the Scandinavian brown bear. *Scientific Reports 2024 14:1* **14**, 1–11 (2024).
77. P. Anijalg, *et al.*, Large-scale migrations of brown bears in Eurasia and to North America during the Late Pleistocene. *J Biogeogr* **45**, 394–405 (2018).
78. C. Bon, *et al.*, Deciphering the complete mitochondrial genome and phylogeny of the extinct cave bear in the Paleolithic painted cave of Chauvet. *Proc Natl Acad Sci U S A* **105**, 17447–17452 (2008).
79. J. Krause, *et al.*, Mitochondrial genomes reveal an explosive radiation of extinct and extant bears near the Miocene-Pliocene boundary. *BMC Evol Biol* **8**, 1–12 (2008).
80. Ó. Ingólfsson, Ø. Wiig, Late Pleistocene fossil find in Svalbard: The oldest remains of a polar bear (Ursus maritimus Phipps, 1744) ever discovered. *Polar Res* **28**, 455–462 (2009).
81. D. E. Granger, N. A. Lifton, J. K. Willenbring, A cosmic trip: 25 years of cosmogenic nuclides in geology. *GSA Bulletin* **125**, 1379–1402 (2013).
82. P. Häuselmann, D. E. Granger, Dating of caves by cosmogenic nuclides: method, possibilities, and the Siebenhengste example. *Acta carsologica* **34**, 45–50 (2005).
83. P. Häuselmann, D. E. Granger, P. Y. Jeannin, S. E. Lauritzen, Abrupt glacial valley incision at 0.8 Ma dated from cave deposits in Switzerland. *Geology* **35**, 143–146 (2007).
84. T. Wagner, *et al.*, Young uplift in the non-glaciated parts of the Eastern Alps. *Science Letters* **295**, 159–169 (2010).
85. C. P. Kohl, K. Nishiizumi, Chemical isolation of quartz for measurement of in-situ-produced cosmogenic nuclides. *Geochim Cosmochim Acta* **56**, 3583–3587 (1992).

86. S. A. Binnie, *et al.*, Preliminary results of CoQtz-N: A quartz reference material for terrestrial in-situ cosmogenic 10Be and 26Al measurements. *Nucl Instrum Methods Phys Res B* **456**, 203–212 (2019).
87. D. Child, G. Elliott, C. Mifsud, A. M. Smith, D. Fink, Sample processing for earth science studies at ANTARES. *Nuclear Instruments and Methods in Physics Research B* **172**, 856–860 (2000).
88. J. Heinemeier, J. Olsen, M. Klein, D. Mous, The new extended HVE 1 MV multi-element AMS system for low background installed at the Aarhus AMS Dating Centre. *Nuclear Instruments and Methods in Physics Research B* **361**, 143–148 (2015).
89. K. Nishiizumi, *et al.*, Absolute calibration of 10Be AMS standards. *Nuclear Instruments and Methods in Physics Research B* **258**, 403–413 (2007).
90. A. Nadachowski, *Late Quaternary rodents of Poland with special reference to morphotype dentition analysis of voles* (Państwowe Wydawn. Naukowe, 1982).
91. J. Niethammer, F. Krapp, *Handbuch der Säugetiere Europas, Band 2: Rodentia II* (Aula Verlag, 1978).
92. Y. Fernández-Jalvo, P. Andrews, *Atlas of taphonomic identifications: 1001+ images of fossil and recent mammal bone modification* (Springer, 2016).
93. A. T. Gondek, S. Boessenkool, B. Star, A stainless-steel mortar, pestle and sleeve design for the efficient fragmentation of ancient bone. *Biotechniques* **64**, 266–269 (2018).
94. C. Giguet-Covex, *et al.*, Long livestock farming history and human landscape shaping revealed by lake sediment DNA. *Nat Commun* **5** (2014).
95. L. S. , Epp, *et al.*, New environmental metabarcodes for analysing soil DNA: potential for studying past and present ecosystems. *Mol Ecol* **21**, 1821–1833 (2012).
96. S. Boessenkool, *et al.*, Blocking human contaminant DNA during PCR allows amplification of rare mammal species from sedimentary ancient DNA. *Mol Ecol* **21**, 1806–1815 (2012).
97. P. Taberlet, A. Bonin, L. Zinger, E. Coissac, *Environmental DNA: For biodiversity research and monitoring* (Oxford University Press, 2018).
98. F. Boyer, *et al.*, obitools: A unix-inspired software package for DNA metabarcoding. *Mol Ecol Resour* **16**, 176–182 (2016).
99. T. Brown, *et al.*, Paleoeconomy more than demography determined prehistoric human impact in Arctic Norway. *PNAS Nexus* **1** (2022).
100. P. W. Brown, L. H. Fredrickson, “White-winged Scoter (Melanitta deglandi)” in *Birds of the World*, P. G. Rodewald, Ed. (Cornell Lab of Ornithology, 2020).
101. J. J. Hatch, *et al.*, “Great Cormorant (Phalacrocorax carbo)” in *Birds of the World*, S. M. Billerman, Ed. (Cornell Lab of Ornithology, 2020).
102. J. Stockmarr, Tables with spores used in absolute pollen analysis. *Pollen et Spores* **13**, 615–621 (1971).
103. K. Faegri, J. Iversen, P. E. Kaland, K. Krzywinski, *Textbook of pollen analysis.*, 4th Ed. (John Wiley & Sons, 1989).
104. I. G. Alsos, *et al.*, Last Glacial Maximum environmental conditions at Andøya, northern Norway; evidence for a northern ice-edge ecological “hotspot.” *Quat Sci Rev* **239**, 106364 (2020).
105. P. Taberlet, *et al.*, Power and limitations of the chloroplast trn L (UAA) intron for plant DNA barcoding. *Nucleic Acids Res* **35**, e14 (2007).
106. I. G. Alsos, *et al.*, Postglacial species arrival and diversity buildup of northern ecosystems took millennia. *Sci Adv* **8**, 7434 (2022).
107. S. Garcés-Pastor, *et al.*, High resolution ancient sedimentary DNA shows that alpine plant diversity is associated with human land use and climate change. *Nat Commun* **13**, 1–16 (2022).
108. J. H. Sønstebø, *et al.*, Using next-generation sequencing for molecular reconstruction of past Arctic vegetation and climate. *Mol Ecol Resour* **10**, 1009–1018 (2010).
109. E. Willerslev, *et al.*, Fifty thousand years of Arctic vegetation and megafaunal diet. *Nature* **506**, 47–51 (2014).
110. E. M. Soininen, *et al.*, Highly overlapping winter diet in two sympatric lemming species revealed by DNA metabarcoding. *PLoS One* **10** (2015).
111. Y. Lammers, *et al.*, Multiplexing PCR allows the identification of within-species genetic diversity in ancient eDNA. *Mol Ecol Resour* **24**, e13926 (2024).
112. D. P. Rijal, M. Falahati-Anbaran, T. Alm, I. G. Alsos, Microsatellite Markers for Heracleum persicum (Apiaceae) and Allied Taxa: Application of Next-Generation Sequencing to Develop Genetic Resources for Invasive Species Management. *Plant Mol Biol Report* **33**, 1381–1390 (2015).
113. S. E. Crump, *et al.*, Ancient plant DNA reveals High Arctic greening during the Last Interglacial. *PNAS* **118**, p.e2019069118 (2021).
114. A. T. Ter Schure, *et al.*, Sedimentary ancient DNA metabarcoding as a tool for assessing prehistoric plant use at the Upper Paleolithic cave site Aghitu-3, Armenia. *J Hum Evol* **172**, 103258 (2022).
